# Burden of illness in tuberous sclerosis complex-associated epilepsy: a systematic literature review of epidemiology, health-related quality of life, costs and resource use

**DOI:** 10.1186/s13023-025-03975-y

**Published:** 2025-11-25

**Authors:** Alexandra Furber, Alison Martin, Andrea Bertuzzi, Fern Wesson, Miranda Harrison, Sally Bowditch, Jamshaed Siddiqui

**Affiliations:** 1 Reviews Department, Crystallise Ltd, Colchester, Essex, UK; 2 Jazz Pharmaceuticals UK Ltd, 80 Charlotte Street, London, W1T 4DF England

**Keywords:** Tuberous sclerosis complex, TSC-associated epilepsy, Burden of illness, Epidemiology, Patient-reported outcomes, Resource use, Family, Caregiver, Healthcare costs

## Abstract

**Background:**

Tuberous sclerosis complex (TSC) is a rare genetic disorder resulting in hamartomas in multiple organs, causing varied manifestations with a substantial burden of illness (BOI) for patients and caregivers. A significant component of the BOI is the high prevalence of TSC-associated epilepsy. The objective of this systematic literature review is to provide an overview of the BOI in TSC-associated epilepsy, a focus not reported in the recent review by Zöllner et al. (2020).

**Methods:**

Following a search of major databases and congress sites to April 2023, published studies covering epidemiology, quality of life (QOL) of patients and their caregivers, direct and indirect costs, resource use and treatment patterns in children and/or adults with TSC were included. Studies on efficacy and safety, and non-neurological TSC manifestations, were excluded. Relevant studies were manually reviewed, double screened and summarised/synthesised. No statistical analyses or bias assessments were conducted.

**Results:**

Relevant articles (n = 241) included 182 reporting global epidemiology data, revealing a wide range of TSC incidence per 100,000 live births (0.153–17.24) and prevalence per 100,000 general population (0.6–12.7). TSC-associated seizures were reported in a mean of 64.1% and 79.8% of adults and children, respectively. Patient-reported outcome (PRO) tools indicated that cognitive impairment and neuropsychiatric disorders (e.g. autism spectrum disorder) frequently occur with TSC-associated epilepsy. The reported BOI is substantial, impacting the QOL of patients, caregivers and the wider family. Additionally, TSC-associated seizures negatively impact QOL, elevate indirect and healthcare costs (e.g. £14,335 vs £4,448), resource use (e.g. hospital admissions, physician visits and impact on patients’ and caregivers’ careers) and risk of mortality (7.53% vs 3.68%) compared with the healthy population or patients with TSC without seizures.

**Conclusion:**

This review summarises the BOI caused by the early onset and refractory nature of TSC-associated epilepsy. Limitations include a lack of recent prevalence data (> 2016), standardised PROs, formal statistical analysis, BOI data in adults and impact on wider family QOL. More robust epidemiological data are needed. Nevertheless, this review supports the importance of early identification and effective seizure management to improve the BOI of TSC-associated epilepsy for patients, caregivers and the wider family, and society.

**Supplementary Information:**

The online version contains supplementary material available at 10.1186/s13023-025-03975-y.

## Background

Tuberous sclerosis complex (TSC) is a rare autosomal dominant disorder that is caused by abnormalities in the tumour-suppressor genes, TSC subunit 1 (*TSC1*) and TSC subunit 2 (*TSC2*), and is characterised by the formation of hamartomas in multiple organ systems of the body [1–4]. Manifestations of TSC include those affecting the renal, pulmonary, cardiac, ocular and central nervous systems [1, 2, 4], and many have a substantial impact that varies across individuals and over each patient’s lifetime, resulting in a complex burden of illness (BOI) [1, 2, 5, 6].

Common clinical consequences of TSC include renal manifestations leading to bleeding and kidney failure, and breathing difficulties due to lymphangioleiomyomatosis (LAM; mostly occurs in female patients). Clinical manifestations of TSC can occur at any age throughout a patient’s lifetime [7, 8]. Diagnosis can be complex because of the heterogeneous nature of the disease; some patients are diagnosed from manifestations observed prenatally (e.g. with cardiac rhabdomyomas), while others will be diagnosed postnatally after their first manifestation [4, 9, 10] typically in the first few years of life following confirmation of mutations in *TSC1/TSC2* by genetic testing [1, 2]. Common neurological manifestations of TSC include epilepsy, which can be associated with sudden unexpected death in epilepsy (SUDEP) and status epilepticus, and TSC-associated neuropsychiatric disorders (TAND), which include neuropsychiatric disorders, behavioural problems and intellectual disability [2, 7, 11–13].

Current literature suggests that over 80% of patients with TSC develop epilepsy [1], which is reported as refractory in 32.9–62.5% of patients, where refractory is defined as having continued seizures despite prior treatment with three or more antiseizure medications (ASMs) [14–16]. Seizures experienced by these patients typically develop in the first few years of life and have an associated risk of autism spectrum disorder (ASD) and cognitive impairment, making early recognition and diagnosis of seizures and effective disease management essential in reducing the potential BOI of these developmental and neurological outcomes [2, 17–19]. Further, there remains an unmet need for interventions to effectively reduce seizures and thus improve the quality of life (QOL) of both patients and caregivers [1, 3]. Currently, only two therapies are approved in the USA and the EU for TSC-associated seizures as opposed to epileptic seizures in general: everolimus and cannabidiol [20, 21].

A previous systematic literature review (SLR) of the BOI experienced by patients with TSC was conducted by Zöllner et al. (2020) based on studies published up to October 2019 [1]. While that review focused on healthcare resource utilisation, QOL, direct costs and general manifestations of TSC [1], the present SLR aims to include more recent data on the BOI experienced by patients with TSC, with a more specific focus on TSC-associated epilepsy, a substantial component of the overall disease burden in TSC [22]. We report an overview of the evidence on the epidemiology, QOL of patients and their caregivers, the treatment patterns observed in these patients, and the direct and indirect costs and resource use related to TSC-associated epilepsy.

## Methods

We conducted an SLR following the Preferred Reporting Items for Systematic Reviews and Meta-Analyses (PRISMA) guidelines [23]. The SLR was not registered; however, the protocol is available at request.

### Outcomes

Articles of interest included information on the following outcomes in patients with TSC, and predominantly those with TSC-associated epilepsy: epidemiology, QOL, economic evaluations, costs and resource use, and impact on work, productivity, education and learning.

### Eligibility criteria

While the SLR collected information according to the full inclusion and exclusion criteria (Additional file 1: Table S1), this publication focuses specifically on reporting on TSC-associated epilepsy. Therefore, eligible studies included those in adults or children (most commonly defined by ages of ≥ 18 and < 18 years, respectively, with the cut-off for adolescents typically between 11 and 16 years), and reported TSC-associated seizures, apart from those on cost and QOL, which could report general TSC with or without epilepsy.

### Literature search

The major databases searched were Medline via EMBASE, EMBASE via ProQuest, Heoro.com and Cochrane library; all databases and other sources such as congress websites are included in Additional file 1: Table S2 and S3. The last search was performed on 26 April 2023; no date limit was applied. An overview of the search strategy is provided in Additional file 1: Table S2; an additional epidemiology search was also run according to Additional file 1: Table S3, which included studies on the epidemiology of TSC regardless of seizure occurrence.

### Data collection, synthesis and quality assessment

Details of the data collection and synthesis process are listed in Additional file 1. Briefly, articles were screened independently by two researchers and any discrepancies were resolved with the project leader; data were extracted by one researcher into an Excel template and checked by a second researcher. During the extraction, where applicable, simple data conversions were conducted to facilitate comparisons; for example, converting absolute numbers to proportions (or vice versa). Additionally, to provide an overview of the prevalence of TSC-associated epilepsy over time, prevalence data were plotted against the last year of data collection or date of publication. Lastly, as QOL data were collected from publications reporting data on mixed populations with TSC, both with and without epilepsy, reporting of the number of studies in each category exceeds the total number of studies identified in the SLR because some studies were counted in both categories.

No formal statistical analyses were conducted; therefore, no estimate was made of publication bias or to statistically explore heterogeneity in outcomes across studies. Sensitivity analyses were not conducted and certainty in the body of evidence for each outcome was not assessed. Risk of bias was determined only for clinical trials reporting efficacy and safety, using the Cochrane risk-of-bias 2 tool. Epidemiological data were calculated by entering all relevant data into a spreadsheet as 1/X live births or general population and using Excel functions to calculate simple mean and median values across all relevant studies. Other epidemiology data (proportions with epilepsy, other TSC manifestations), mortality rates and treatment patterns were reported as percentages. Health-related QOL was reported as mean scores. Direct costs were reported as mean or total costs in the source currency. Indirect costs were reported as percentages and mean or total costs in the source currency.

## Results

### Literature search and general findings

**Fig. 1:**
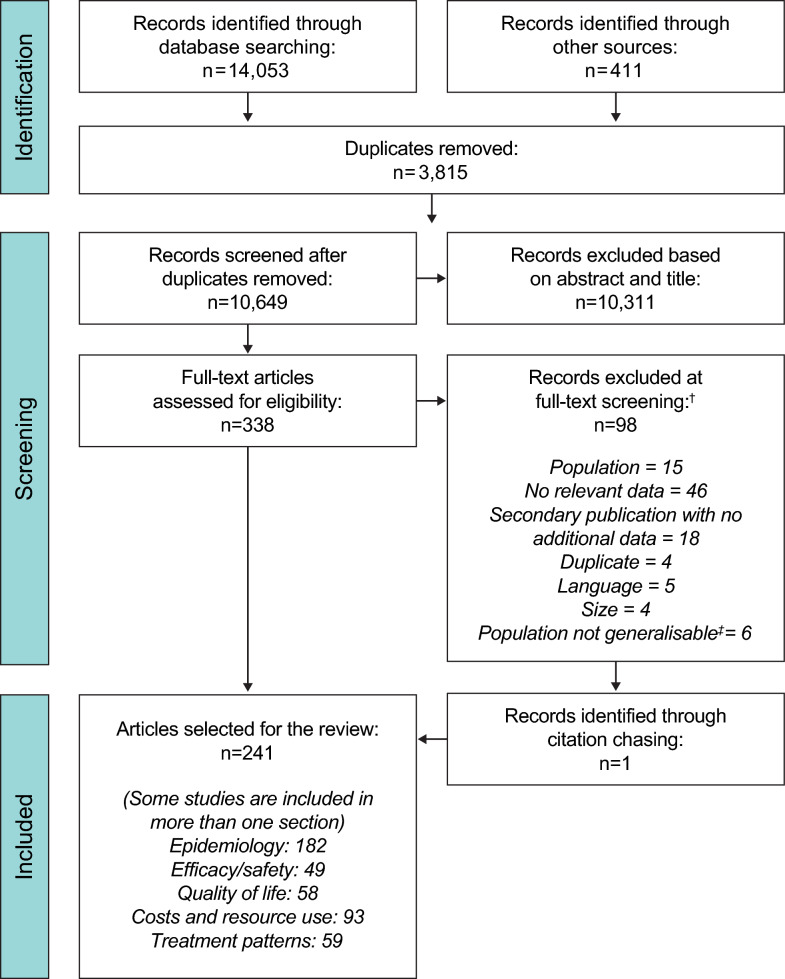
PRISMA diagram. ^†^Includes records that were removed following completion of the SLR and review of key information to refocus the SLR on TSC-associated epilepsy. ^‡^Studies were not generalisable and, therefore, not appropriate for inclusion in the epilepsy risk analysis because they either included a population at higher risk of epilepsy than the general population with TSC, or the population consisted entirely of patients with TSC-associated epilepsy. PRISMA: Preferred Reporting Items for Systematic Reviews and Meta-Analyses; SLR: systematic literature review; TSC: tuberous sclerosis complex

All studies were screened for eligibility (Fig. 1) and the initial search returned 14,053 articles from database searching and 411 articles from other sources. After removal of duplicates, 10,649 articles were reviewed based on the title and abstract, with 10,311 subsequently excluded; the remaining 338 articles underwent full-text review against the inclusion/exclusion criteria, with a further 98 excluded (see Additional file 1: Table S4 and Fig. 1), resulting in 241 unique articles included in the review. Of these, 182 (76%) articles were identified that reported on the epidemiology of TSC; data were extracted from these based on the outcomes listed in Additional file 1.

### Bias assessment

**Fig. 2:**
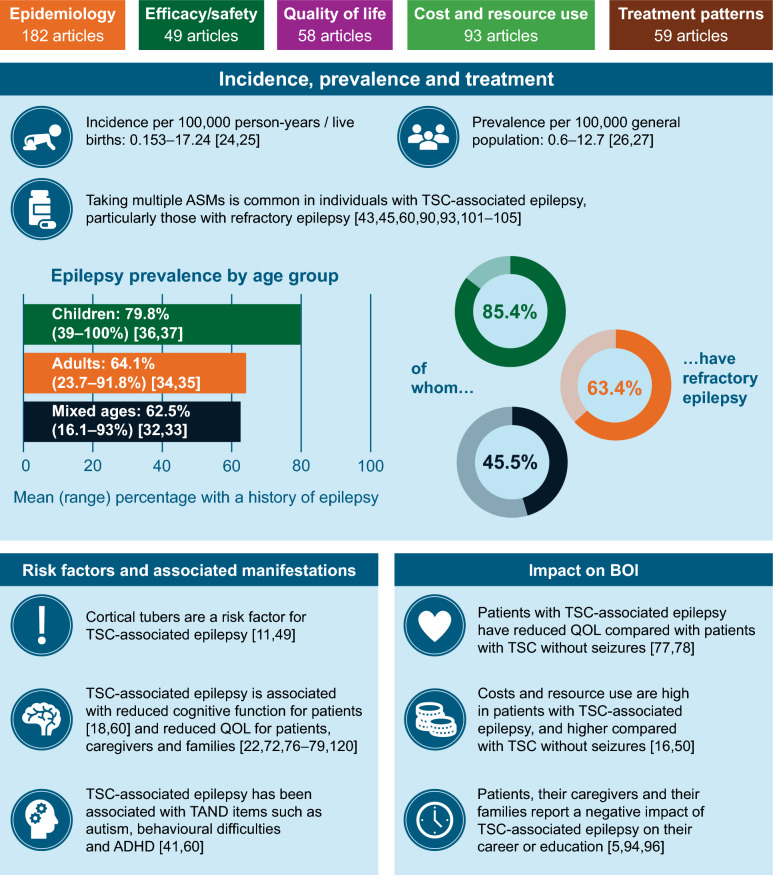
Summary of key findings on the BOI of TSC-associated epilepsy. ADHD: attention deficit hyperactivity disorder; ASM: antiseizure medication; BOI: burden of illness; QOL: quality of life; TAND: tuberous sclerosis complex-associated neuropsychiatric disorders; TSC: tuberous sclerosis complex

Using the Cochrane risk-of-bias 2 tool, 1/5 articles reporting randomised clinical trials and 11/18 articles reporting non-randomised clinical trials had some overall risk of bias. Reasons for risk of bias included participants’, caregivers’ and assessors’ awareness of the assigned intervention, uncertainty over allocation sequence procedures and uncertainty over the measurement potentially differing between intervention groups. Figure 2 shows an overview of the BOI of TSC and TSC-associated epilepsy based on the findings of the review.

### Epidemiology

#### Incidence and prevalence of TSC

**Fig. 3:**
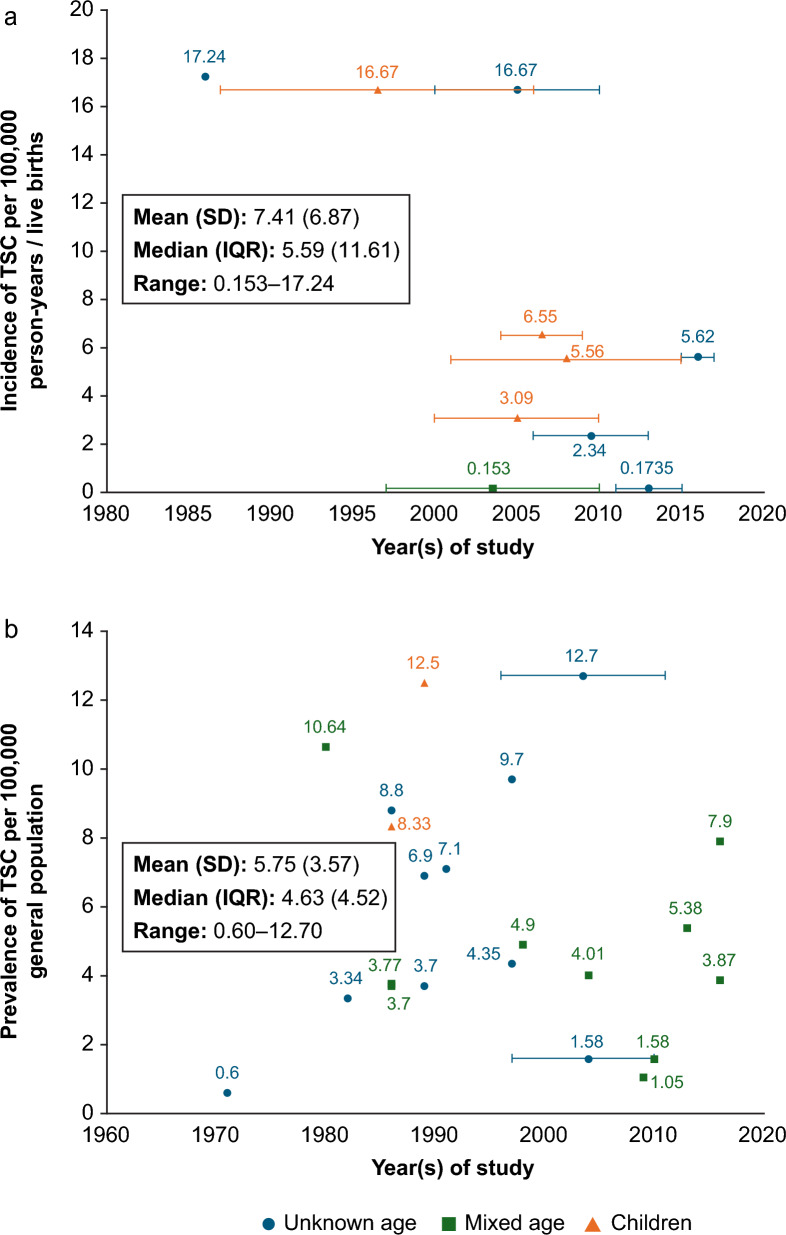
Incidence (a; n = 10 studies) and prevalence (b; n = 17 studies) of TSC. References: (a) Incidence [24, 25, 28, 31, 37, 126–130]; (b) Prevalence [16, 25–27, 29, 30, 33, 52, 55, 60, 131–137]. Ranges shown indicate the years of the study. Year of the study was unknown for the incidence value of 12.5 reported in Japan and was assumed to be the year prior to the publication (i.e. 1989 for the 1990 study cited in Hunt et al. 1993) [33]. Green squares are mixed-age populations and orange triangles are paediatric populations (age ≤ 18 years). Blue circles represent populations of unknown age. IQR: interquartile range (difference between quartile 1 and quartile 3); SD: standard deviation; TSC: tuberous sclerosis complex

Thirty-one of 182 epidemiology articles reported incidence and/or prevalence of TSC in a range of populations; of these 31 articles reporting suitable data, 14 used unclear criteria to make a TSC diagnosis. Based on the remaining 17 articles, the range of incidence of TSC per 100,000 live births across the globe since 1986 was 0.153 to 17.24 [24, 25], and the prevalence of TSC per 100,000 general population since 1971 was 0.6 to 12.7 (Fig. 3a and b) [26, 27]; the lowest values of each range were in Asian countries [25, 26, 28–30] and large incidence values were reported in earlier studies [24, 31].

There are signs that the prevalence of TSC remains relatively stable; however, two articles, reporting prevalence at different timepoints, showed signs of increasing prevalence of TSC over time [25, 26]. A 40-fold increase in prevalence of TSC was reported in Taiwan between 1997 and 2010 [25]. Similarly, another study reported increases in prevalence over time in the UK (0.67/100,000 in 1956 to 3.7/100,000 in 1989) and Japan (3.2/100,000 in 1981 to 9.7/100,000 in 1997), but a decrease in the USA (10.6/100,000 in 1985 to 7.1/100,000 in 1991); however, these results should be interpreted in the context that varying criteria were used (e.g. some timepoints used Vogt’s triad to define TSC, while others used Gomez’s criteria) and the results they presented were average prevalences [26].

#### Prevalence of TSC-associated epilepsy

We identified 63 papers that contained data on the prevalence of epilepsy within the population being evaluated (Additional file 2: Table S1). We calculated the mean percentage prevalence of a mixed-age population, adults or children with TSC-associated epilepsy to be 62.5% (range 16.1% [32] to 93% [33]), 64.1% (range 23.7% [34] to 91.8% [35]) or 79.8% (range 39% [36] to 100% [37]), respectively.

Of those with TSC-associated epilepsy, a mean of 45.5% of mixed-age patients (range 19% [38] to 67.8% [39]), 63.4% of adults (range 0% [40] to 66.1% [41]) and 85.4% of children [42] had refractory epilepsy. The median percentage of patients with TSC who had infantile spasms was 47.0% (range 1.7% to 74.0%) [33, 43]. Among the 24 articles on prognosis found by restricting data to a general population with TSC who were not receiving one specific type of intervention, seizure freedom was achieved in 28.6% of patients overall in nine articles reporting this outcome in adults or mixed-age populations, ranging from 12% [44] to 62.5% [45].

**Fig. 4:**
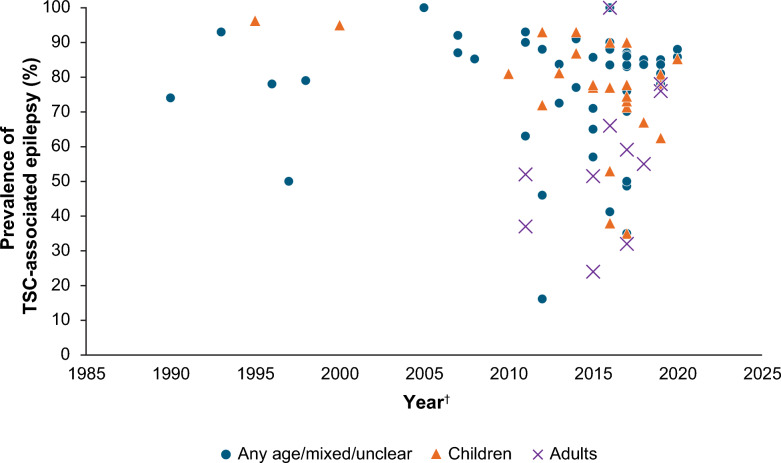
Prevalence of TSC-associated epilepsy among TSC studies (n = 63 studies). ^†^Last year of data collection. Where not specified, this is the year of publication. TSC: tuberous sclerosis complex

The proportion of patients diagnosed with TSC who have epilepsy did not show a clear trend over time across studies with different data collection periods (Fig. 4). In the articles we found, risk factors for development of TSC-associated epilepsy included mutation type and other TSC manifestations. *TSC2* mutations were more prevalent than *TSC1* mutations in people with TSC-associated epilepsy [19, 35, 46]. One study specifically reported the proportions of patients with epilepsy as being 86% for patients with *TSC2* mutations, but only 35% for patients with *TSC1* mutations [47]. Patients with *TSC2* mutations had more severe seizures, were diagnosed 9–11 years earlier and were significantly more likely to have intellectual disabilities than those with *TSC1* mutations [9, 11, 48].

Tubers in the brain can act as epileptic foci; therefore, manifestations of TSC are important in determining the risk of TSC-associated epilepsy. Regression analysis suggested that the strongest risk factor for predicting epilepsy in TSC was tubers (odds ratio [OR] 5.1, 95% confidence interval [CI] 2.5–10.9, compared with patients without tubers) [49]. Additionally, multivariate analysis by Jeong and Wong (2016) suggested that along with cardiac rhabdomyomas (OR 1.9, *P* = 0.04), cortical tuber hamartomas (OR 3.4, *P* = 0.002) independently predicted TSC-associated epilepsy [11].

#### Other TSC manifestations in patients with TSC-associated epilepsy

A total of 103 articles were identified that reported epidemiological data on other relevant manifestations. Manifestations common in patients with TSC-associated epilepsy, according to our median calculations across all 103 articles, were speech/language problems (median 69.0%; range 15.8% [50] to 89.0% [51]), cognitive problems (median 60.0%; range 8.0% [52] to 90.0% [53]), attention deficit hyperactivity disorder (ADHD) (median 11.4%; range 4.3% [50] to 62.0% [54]), ASD (median 22.0%; range 4.9% [55] to 70.0% [56]), behavioural disorders (median 34.0%; range 6.0% [57] to 86.0% [58]) and sleep disorders (median 18.9%; range 5.7% [59] to 86.0% [58]). Taking speech and language problems as an example of the variation between studies, 15.8% of patients with TSC-associated epilepsy experienced this manifestation in one study [50] but, in the first analysis of the EPISTOP study, 89% of children with TSC did not have a normal language developmental quotient [51].

When comparing manifestation rates between patients with TSC with/without epilepsy, psychiatric disorders were more common overall in those with epilepsy (51.9–88%) than without (22.9–74%) [41, 60], and patients with TSC-associated epilepsy were more likely to have ASD, behavioural difficulties, schizophrenia spectrum disorders and ADHD. Evidence was contradictory on whether TSC-associated epilepsy is associated with a lower [41] or higher rate of psychiatric disorders, including anxiety and depression [39].

Several articles reported an association between intellectual disability and learning disorders, and risk of seizures in patients with TSC—mostly with epilepsy/seizures as the dependent variable (outcome) [55, 61–63]. Focusing on neurological manifestations, seizures were more common and more severe in patients with TSC and ASD compared with those without ASD [19, 53, 64, 65], and cognitive or developmental disorders were more common in patients with TSC-associated epilepsy (37%) than patients with TSC without seizures (7%) [60].

Several studies described the impact of seizures in early childhood. Infantile spasms were associated with cognitive impairment [17, 66], which was also found to correlate with higher seizure frequency [18], increased prevalence of refractory epilepsy [67] and severity of epilepsy [66]. Furthermore, early seizure onset (before 12 months of age) was associated with cognitive impairment and ASD [17–19]. Similarly, another study found that, for patients with TSC-associated epilepsy, worse adaptive functioning was associated with lower age at seizure onset [68]. The same study also found that an increased number of ASMs, which can be considered as a proxy for epilepsy control, was associated with cognitive decline [68]. Lastly, a study showed that early diagnosis and intervention with sirolimus and vigabatrin for children with TSC helped to reduce the occurrence of epilepsy, development of refractory epilepsy and, in turn, prevented adverse developmental outcomes, including cognitive, language and motor deficits [69].

#### Mortality

TSC-associated epilepsy was linked with a shorter life expectancy than TSC without seizures: Strzelczyk et al. (2021) reported 10-year mortality rates of 7.53% and 3.68% in patients with TSC with and without epilepsy, respectively [60]. We found six studies reporting mortality over time and only one reported mortality (7.8% over 8.82 years in Sweden) in patients with TSC-associated epilepsy specifically [16]. A mortality rate of 8.6% was reported over 13 years in France for patients with TSC, 70% of whom had epilepsy [57]. Interestingly, this study found similar survival rates 5 years after diagnosis of TSC in patients with epilepsy (93%) and those without epilepsy (94%) [57].

The main non-epilepsy-related causes of death for patients with TSC were renal complications (27.5–50.0%), subependymal giant cell astrocytoma (SEGA; 6.3%), LAM (10.0–12.5%), other brain tumours and other malignancies (6.3–25.0%) [12, 16, 70]. Epilepsy-related causes of death were seizures (10.0%, in patients with TSC-associated epilepsy specifically) [16], status epilepticus (up to 32.5% in patients with TSC and severe mental handicaps) [70] and SUDEP (25.0%) [12].

### Health-related QOL

#### TAND checklist in children and adults

**Fig. 5:**
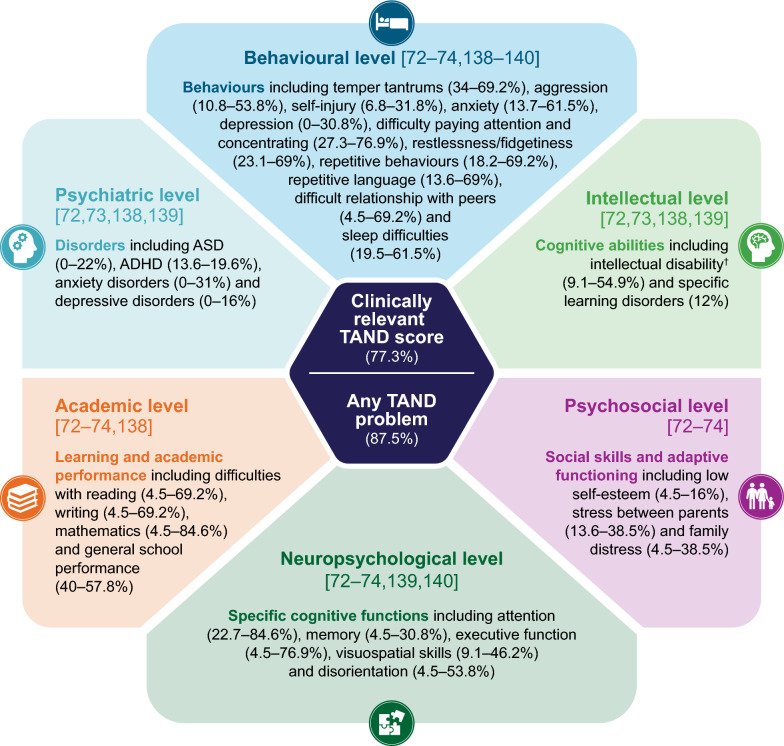
Prevalence of TAND in children or adults with TSC (n = 8 studies). ^†^Includes mild, moderate, severe and profound intellectual disability. References are detailed within the figure. Of the eight studies identified, two had no relevant data for the figure [48, 71]. Of the eight studies identified, two had no relevant data for the figure [48, 71]. ADHD: attention deficit hyperactivity disorder; ASD: autism spectrum disorder; TAND: tuberous sclerosis complex-associated neuropsychiatric disorders; TSC: tuberous sclerosis complex

We identified eight studies that assessed patients using the TAND checklist using tools such as the Social Responsiveness Scale (SRS-2) [71] to assess TAND and TSC-associated epilepsy. Findings from these studies illustrated that clinically relevant TAND scores were reported for most children with TSC (77.3% of whom had epilepsy) [72] and could impact the wider family, as evidenced by one study showing a correlation between social impairment associated with ASD and family distress [71]. Two studies determined the impact of TSC-associated epilepsy on TAND, finding variable evidence of worse outcomes compared with patients with TSC without seizures [73, 74]. Among these, repetitive behaviours, poor neuropsychological skills, difficulty paying attention, ASD and multitasking were worse or more prevalent TAND items in patients with more severe TSC-associated epilepsy [73, 74]. TAND items that were significantly more prevalent in patients with active TSC-associated epilepsy versus those with seizure freedom or no history of seizures included absent or delayed onset of language, repetitive speech and repetitive behaviours (behavioural level); low levels in spelling and mathematics (scholastic category); and dual-tasking, visuo-spatial skills and executive skills (neuropsychological category) [74]. However, one of the studies (n = 42) also found that rates of anxiety and depression were numerically higher in patients with TSC without seizures (53.8% and 7.7%, respectively) compared with those with TSC-associated epilepsy (38.5% and 0%) [74] (Fig. 5).

#### Patient-reported outcomes (PROs) and associated QOL in children with TSC with or without epilepsy

We identified 19 articles that reported PROs in children with TSC with (n = 8), with/without (n = 8) or without (n = 3) epilepsy (Table 1). Two studies reported PROs with domains related to intellect and cognition in children with or without epilepsy, and four other studies reported PROs used to assess the effect of TSC on ASD in children with TSC, most (73.1–95.2%) of whom had epilepsy.

**Table 1:** PRO outcomes and associated QOL for children with TSC with (n = 8), with/without (n = 8) or without (n = 3) epilepsy (n = 19 studies)

Study and patient population	PRO tool	PRO scores	Determinants of poorer scores
Amin 2019 [76] • Children (aged ≤ 18 years) with TSC and epilepsy	PedsQL	Mean (SD) total scores: parent proxy = 43 (21); child self-report = 67 (21)	• Parent-proxy reporting vs child self-reporting
Fong 2019 [77] • Children (aged 2–18 years) with TSC (97% had history of seizures)	PedsQL 4.0	Mean total scores = 53.4–63.2 Median physical health summary score = 55.6–75 Mean psychosocial health summary score = 52.6–59.8	• < 2 vs ≥ 2 years at onset • Generalised vs focal seizures (similar scores for ≤ 1 vs > 1 seizure per month)
Ding 2021 [78] • Children (aged 2–18 years) with TSC (85% had epilepsy)	PedsQL 4.0	Epilepsy, mean (SD), vs HCs:*** Total score = 65.0 (19.7) Physical health summary score = 77.6 (22.9) Psychosocial health summary score = 58.0 (21.3) *HCs, mean (SD):* Total score = 83.6 (14.3) Physical score = 87.2 (16.9) Psychosocial score = 82.8 (15.9)	*Multiple linear regression associations:* • *TSC2* mutation vs no mutation* • Epilepsy vs no epilepsy* • Age < 2 vs > 2 years at seizure onset** • Epilepsy > 2 vs < 2 years** • > 2 vs < 2 ASMs** • High vs not high seizure frequency** • Mild/moderate ID vs normal IQ*** • Severe/profound ID vs normal IQ*** • TAND vs no TAND***
Willems 2021 [79] • Children (aged 4–17 years) with TSC (epilepsy was the first symptom for 47%)	KINDL	*Epilepsy, mean (SD), vs HCs:* Total HRQOL score = 67.9 (12.7)* Physical score = 79.9 (13.9)* Emotional score = 69.5 (12)* Self-esteem score = 65.8 (18)* Family score = 79.2 (12.2) Friend score = 64 (19.3)* Education score = 70.1 (16.8)* *HCs, mean (SD):* Total HRQOL score = 76.3 (10.1) Physical score = 76.5 (17.3) Emotional score = 80.8 (12.8) Self-esteem score = 68.8 (14.2) Family score = 77.7 (14.3) Friend score = 78 (13.4) Education score = 76 (16)	• Older age (e.g. 14–17 vs < 14 years) • No epilepsy vs epilepsy • Affected vs no affected siblings • > 10 vs < 10 years of disease* • None vs psychiatric manifestations* • LAEP ≥ 35 vs < 35*
Asano 2001 [115] • Children (aged ≤ 16 years) with TSC and epilepsy	GARS	Mean total scores = 62–104.4	• Autism vs mental disability and normal IQ • Mental disability vs normal IQ
Yates 2011 [116] • Children (aged 0–16 years) with TSC (62% had epilepsy)	VABS	*Adaptive level, % of patients:* Moderate high = 2 Adequate = 25 Moderate low = 18 Impaired = 36	• Impaired vs normal ability range
Schoenberger 2020 [117] • Children (aged 3–36 months) with TSC enrolled in TACERN (78% developed seizures by 36 months)	VABS-II	*Mean (SD) adaptive behaviour composite:* 6 months old = 90 (14) 36 months old = 81.5 (16.2)	• Higher seizure frequency • Lower vs higher age at seizure onset
Wu 2019 [118] • Infants (aged ≤ 7 months, seizure-naïve) with TSC	VABS-II	*Mean scores:*^*†*^ 6 months old = 94.8–100.35 24 months old = 76.7–101.75	• Pre-seizure EA with seizures vs pre-seizure EA without seizures* / never had seizures* at 24 months
Capal 2017 [18] • Children (aged 0–36 months) with TSC	VABS-II	*Mean (SD) scores:* 6 months old: IS = 93.6 (15); no IS = 91.4 (14.5) 24 months old: IS = 86.3 (17.8); no IS = 100.7 (7.6)	• IS vs no IS at 24 months***
Bebin 2016 [119] • Children (aged > 7 months) (mean age at enrolment 82.4 days) with TSC	VABS	*Mean scores:* 6 months old = 86–100.5 24 months old = 76.5–105.2	• Pre-seizure EA with seizures vs pre-seizure EA without seizures / no pre-seizure EA / EA without seizures / normal EEG without seizures / pre-treatment
Asano 2001 [115] • Children (aged ≤ 16 years) with TSC and epilepsy	VABS (OABC)	Mean total scores = 50–78.3	• Autism vs mental disability and normal IQ • Mental disability vs normal IQ
Stomberg 2021 [120] • Children (aged < 18 years) with TSC-associated epilepsy	VABS-II	*Mean (SD) general developmental level:* No surgery = 58 (20.3) Surgery = 67.8 (22.1) No surgery, seizures = 54.4	• No surgery vs surgery* • No surgery, seizures vs: no surgery, seizure-free / surgery, seizures** / surgery, seizure-free***
Yates 2011 [116] • Children (aged 0–16 years) with TSC (62% had epilepsy)	MSEL	*Descriptive category, % of patients:* Above average / very high = 3 Average = 11 Below average = 20 Impaired = 65	• Impaired vs normal ability range
Schoenberger 2020 [117] • Children (aged 3–36 months) with TSC enrolled in TACERN (78% developed seizures by 36 months)	MSEL	*Mean (SD) composite score:* 6 months old = 92.3 (17.9) 36 months old = 81.4 (23.5)	• Higher seizure frequency • Lower vs higher age at seizure onset
Wu 2019 [118] • Infants (aged ≤ 7 months, seizure-naïve) with TSC	MSEL	*Mean scores:*^*†*^ 6 months old = 81.1–100.2 24 months old = 57.3–102.5	• Pre-seizure EA with seizures vs pre-seizure EA without seizures** / never had seizures** at 24 months
Capal 2017 [18] • Children (aged 0–36 months) with TSC	MSEL	*Mean (SD) scores:* 6 months old: IS = 91.2 (16.5); no IS = 95.3 (19.8) 24 months old: IS = 67 (15.8); no IS = 94.7 (20.7)	• IS vs no IS at 24 months***
Bebin 2016 [119] • Children (aged > 7 months) (mean age at enrolment 82.4 days) with TSC	MSEL	*Mean scores:* 6 months old = 75.3–103.6 24 months old = 57.3–102.5	• Pre-seizure EA with seizures vs pre-seizure EA without seizures / no pre-seizure EA / EA without seizures / normal EEG without seizures / pre-treatment
Capal 2017 [18] • Children (aged 0–36 months) with TSC	PLS-5	*Mean (SD) scores:* 6 months old: IS = 94.5 (16.2); no IS = 99.8 (18.4) 24 months old: IS = 78.6 (15.9) no IS = 99.4 (14.1)	• IS vs no IS at 24 months***
Granader 2010 [121] • Children (aged 4–18 years) with TSC (95.2% had epilepsy)	SRS, SCQ	Mean (SD) SRS total score = 69.7 (17.4) Mean (SD) SCQ total score = 13.1 (7.5)	• NA
Capal 2017 [18] • Children (aged 0–36 months) with TSC	AOSI, ADOS	Mean (SD) AOSI total score: IS = 10.7 (6.9), no IS = 5.5 (3.5) Mean (SD) ADOS score: IS = 11 (8.4); no IS = 5.8 (6.6)	• AOSI: IS vs no IS at 12 months*** • ADOS: IS vs no IS at 24 months*
Patel 2017 [65] • Children with TSC (71.4% had epilepsy)	ADOS	Mean scores = 11, 5.8	• Early seizure onset (< 24 months) vs seizure-free infants
Krueger 2013 [122] • Children (aged 2–21 years) with TSC with epilepsy	NCBRF, QOLCE	*Median change from baseline at 16 weeks of EVE (5 mg/m*^*2*^*/day):* NCBRF positive domain (total) = 1.5 NCBRF negative domain (total) = − 28.2*** Overall QOLCE = 1***	• No intervention vs EVE
Nabbout 2017 [123] • Children (aged 2–11 years) with TSC and epilepsy (PORS)	QOLCE	*Mean change in overall score from baseline at 18 weeks:* EVE (3–7 ng/mL) = 3.1 EVE (9–15 ng/mL) = 4.0 Placebo = 1.7	• Placebo vs EVE
de Vries 2018 [75] • Children (aged 2–11 years) with TSC and epilepsy (PORS)	QOLCE, QOLIE-AD-48	*Mean (SD) QOLCE scores at baseline:* WNV: < 70 = 48.1 (13.8); ≥ 70 = 55.9 (11.4) *Mean (SD) QOLIE-AD-48 scores at baseline:* WNV: < 70 = 55.8 (16.9); ≥ 70 = 66.5 (14.3) *Mean changes at Week 12 of EVE:* QOLCE = 1.7 (non-responders); 5.8 (responders) QOLIE-AD-48 = 2.6 (non-responders), 8.2 (responders)	• Total QOLCE score: WNV < 70 vs ≥ 70** • Total QOLIE-AD-48 score: WNV < 70 vs ≥ 70 • Change in QOLCE: non-responders to EVE vs responders* • Change in QOLIE-AD-48: non-responders to EVE vs responders
Ebrahimi-Fakhari 2019 [72] • Children (aged < 18 years) with TSC (77% had epilepsy)	PSI	*Mean (SD) score; clinically relevant total, %:* Total stress score = 58.7 (9.9); 54.5 Child stress score = 59.2 (9.3); 59.1 Parental stress score = 57.3 (11.2); 50	• Child stress: developmental delay* • Parental stress: TAND externalising score,* TAND total score,* CBCL total score,** number of ASMs*
Roth 2011 [124] • Children with TSC and epilepsy post-surgery for refractory seizures (aged 8 months to 17 years)	10-question questionnaire (scale: − 10 [worst] to 10 [best])	*Mean (SD) score:* % of patients with improvement / no improvement = 64.5 (13.7) / 27.1 (12.2) Seizures = 7.85 (3.54) Language = 4.54 (4.52) Social life = 5.87 (3.92) School performance = 5.92 (3.65) ADL independency = 4.55 (3.87) Family QOL = 6.84 (4.45) Time on epilepsy therapy = 6.64 (4.16)	• No surgery vs surgery
Thiele 2021 [106] • Children (aged 2–18 years) with TSC and epilepsy	QOLCE	*Change from baseline (95% CI):* Placebo = 2.4 (− 1.6, 6.4) CBD (25 mg/kg/day) = 2.9 (− 1.3, 7) CBD (50 mg/kg/day) = 2.2 (− 1.8, 6.3)	• Placebo vs CBD (25 mg/kg/day) • CBD (50 mg/kg/day) vs placebo
Stomberg 2021 [120] • Children (aged < 18 years) with TSC-associated epilepsy	DISABKIDS	*Mean (SD) QOL:* No surgery = 62.9 (16.4) Surgery = 64 (15.2) No surgery, seizures = 35.3	• No surgery vs surgery • Similar scores in subgroups by seizures
Stomberg 2021 [120] • Children (aged < 18 years) with TSC-associated epilepsy	SDQ-D	*Mean (SD) social adaptation:* No surgery = 16.1 (5.2) Surgery = 15.7 (5.7) No surgery, seizures = 21.3	• No surgery vs surgery • Similar scores in subgroups by seizures
Stomberg 2021 [120] • Children (aged < 18 years) with TSC-associated epilepsy	GEOS subscale	*Mean (SD) concerns about seizures:* No surgery = 28.6 (15.8) Surgery = 20.4 (12.5) No surgery, seizures = 46.1	• No surgery vs surgery* • No surgery, seizures vs: no surgery, seizure-free* / surgery, seizures / surgery, seizure-free***
Stomberg 2021 [120] • Children (aged < 18 years) with TSC-associated epilepsy	IOFS	*Mean (SD) scores:* No surgery = 26.9 (9.2) Surgery = 26.4 (8.9) No surgery, seizures = 40.3	• Surgery, seizures vs no surgery, seizures*

^*^P < 0.05; **P < 0.01; ***P < 0.001

^†^Data extracted from figure using webplotdigitizer: https://apps.automeris.io/wpd/

ADL: activities of daily living; ADOS: Autism Diagnostic Observation Scale; AOSI: Autism Observation Scale for Infants; ASM: antiseizure medication; CBCL: Child Behaviours Check List; CBD: cannabidiol; CI: confidence interval; DISABKIDS: quality of life assessment in children with chronic health conditions and disabilities; EA: epileptiform activity; EEG: electroencephalography; EVE: everolimus; GARS: Gilliam Autism Rating Scale; GEOS: Glasgow Epilepsy Outcome Scale; HC: healthy control (e.g. general population); HRQOL: health-related quality of life; ID: intellectual disability; IOFS: Impact-on-Family Scale; IQ: intelligence quotient; IS: infantile spasm; LAEP: Liverpool Adverse Event Profile; MSEL: Mullen Scales of Early Learning; NA: not applicable; NCBRF: Nisonger Child Behavior Rating Form; OABC: Overall Adaptive Behaviour Composite; PedsQL: Paediatric Quality of Life Inventory; PedsQL 4.0: PedsQL Version 4.0; PLS-5: Pre-school Language Scale, Fifth Edition; PORS: partial-onset refractory seizures; PRO: patient-reported outcome; PSI: Parental Stress Index; QOL: quality of life; QOLCE: Quality of Life for Children with Epilepsy; QOLIE-AD-48: 48-item Quality of Life in Epilepsy Inventory for Adolescents; SCQ: Social Communication Questionnaire; SD: standard deviation; SDQ-D: Strengths and Difficulties Questionnaire (German version); SRS: Social Rating Scale; TACERN: TSC Autism Center of Excellence Network; TAND: tuberous sclerosis complex-associated neuropsychiatric disorders; TSC: tuberous sclerosis complex; *TSC2*: TSC subunit 2; VABS: Vineland Adaptive Behaviour Scale; VABS-II: VABS, Second Edition; WNV: Wechsler Non-Verbal

From the remaining studies that used a variety of PRO tools investigating patient and caregiver QOL (Quality of Life for Children with Epilepsy [QOLCE]; Paediatric Quality of Life Inventory Version 4.0 [PedsQL 4.0]; PedsQL; Parental Stress Index [PSI] and KINDL), it was reported that patients with TSC-associated epilepsy, their caregivers and families have high levels of stress and low QOL. Low QOL in patients with refractory TSC-associated epilepsy was associated with issues with physical restrictions, language and attention/concentration in childhood epilepsy, according to a post-hoc analysis of the EXIST-3 phase 3 trial of everolimus [75]. Additionally, another study showed that QOL can be worse in children with TSC because of epilepsy-related outcomes [76–78]. QOL impacted by TSC-associated epilepsy also led to elevated stress for the child and parents, as well as reduced QOL for the patient’s siblings [72, 79].

#### PROs and associated QOL in mixed-age populations with TSC with or with/without epilepsy

We identified 14 studies that reported PROs in a mixed-age population (adults, both adults and children, or age undefined) with TSC with (n = 11) or with/without (n = 3) epilepsy (Additional file 2: Table S2). While some studies reported PROs assessing social interaction, sleep quality and intellectual level [80–82], several reported PROs assessing QOL. These PROs included the Quality of Life in Epilepsy Inventory (QOLIE) and similar measures focused on problems (31-item Quality of Life in Epilepsy Inventory–Problems [QOLIE-31-P]) for adults, and the 48-item Quality of Life in Epilepsy Inventory for Adolescents (QOLIE-AD-48) [75, 80]. While there were studies reporting PROs in mixed-age populations for TSC-associated epilepsy alone [75, 80, 83], those in adults were mixed populations that included those with and without epilepsy [40, 84, 85].

Similar to findings in children, epilepsy was a key determinant of worse QOL in mixed-age populations, based on eight of the studies [56, 75, 80, 82, 83, 85–87]. For example, in two studies that looked at transition from paediatric to adult healthcare, most described their QOL as fair (38.0%) or mediocre (34.0%) [83]; ‘attitudes towards epilepsy’, and ‘seizure worry’ and ‘social function’ are particular concerns for adolescents and adults, respectively [75].

Again, similar to studies reporting PROs in children, there was evidence based on seven studies that effective seizure management improves patient QOL in mixed-age populations. For example, in two studies, patients in epilepsy remission reported a higher QOL than patients with refractory epilepsy [85], and nearly all family members of patients with TSC-associated epilepsy felt that seizure management improved the patient’s QOL [22]. Similar findings were reported in patients undergoing epilepsy surgery, where significant improvements in QOLIE-31 scores, as well as in Wechsler Intelligence Scale (Chinese Revision) (WIS-CR) scores for those with a low preoperative intelligence quotient (IQ), were observed in patients who achieved seizure freedom [80].

#### QOL in caregivers of patients with TSC with or without epilepsy

A total of four studies were identified that assessed the QOL of caregivers of patients with TSC with (n = 2), with/without (n = 1) or without (n = 1) epilepsy (Table 2). Only one study was found that investigated how seizure frequency impacted the QOL of caregivers of patients with TSC and used the Hospital Anxiety and Depression Scale (HADS) and PedsQL family impact module. The study found that caregivers of patients with more versus fewer seizures (> 12 vs 1–12 in the previous week) had higher anxiety and lower QOL [88]. An additional study interviewed caregivers and families for insight into how seizure management could impact QOL. Parents of children with TSC-associated epilepsy reported that epilepsy is a constant feature in their lives, patients need to be under constant supervision and that engaging in normal family activities was inhibited. However, medications effective in reducing seizure frequency and severity were felt to improve QOL even if the patient still had seizures [22].

**Table 2:** QOL in caregivers of patients with TSC with (n = 2), with/without (n = 1) or without (n = 1) epilepsy (n = 4 studies)^†^

Study and patient population	PRO tool	Scores	Determinants of poorer scores
Skrobanski 2023 [88] • Caregivers (aged ≥ 18 years) of children and adults (mean age 20 years) with TSC and epilepsy	HADS, PedsQL FIM	*HADS, mean (SD) score:* Anxiety summary score = 11.2 (4.8) Depression summary score = 7.9 (4.4) *PedsQL FIM, mean (SD) score:* Total scale score = 43.3 (19.1) Family functioning summary score = 46.2 (23) Parent HRQOL summary score = 45.4 (20.3) *Chronic health conditions in caregivers:* Sleep problems = 28% Stress = 23% Anxiety = 18% TSC = 1 caregiver	*HADS:* • > 12 vs 1–12 seizures in previous week (anxiety, depression) • Younger vs older patient age (anxiety*) *PedsQL FIM:* • > 12 vs no seizures in previous week (all scores*) • TAND vs no TAND (total,* family functioning,* parent HRQOL**)
Waltereit 2021 [125] • Caregivers (aged 22–58 years) of patients with TSC	Questionnaire	*Response, % of caregivers:* Believes QOL would improve with psychotherapy = 70 Is unsure whether QOL would improve with psychotherapy = 20 Does not think QOL would improve with psychotherapy = 10	• NA
Willems 2021 [79] • Caregivers of children and adults (aged 0.7–21.8 years) with TSC (47% had seizure as first TSC symptom)	BDI-II	Mean (SD) = 13.4 (10.3) *Caregivers, %:* Score above pathological cut-off = 45.7 Mild symptoms of depression = 26.1 Moderate symptoms of depression = 11.4 Severe symptoms of depression = 8.2	• Lower patient HRQOL* • More drug-related patient AEs* *Predictors of depression:* • Female vs male caregiver* • > 1 family member with TSC* • Child attending special facility* • Child with increased need for care* • Child with TAND*
McDonald 2019 [22] • Caregivers of patients (ages 5–38 years reported) with TSC and epilepsy	Free-text questionnaire	*Overarching statement:* ‘TSC rules our life’ *‘Our normal’ theme*: ‘constant supervision’, ‘robbed us of family life’, ‘setbacks with seizures’, ‘distress of trialling different medications’ *‘Burnout’ theme*: ‘invalidation contributing to burnout’, ‘no respite’, ‘who advocates for the carer?’ *‘Seizure management has given us our lives back’ theme:* ‘predictability is positive’, ‘connected to emotions’, ‘cautious hope’	• NA

^*^P < 0.05; **P < 0.01

^†^The number of studies for patients ‘with’ epilepsy or ‘without’ epilepsy exceeds the total number of studies because studies in which some, but not all, patients had epilepsy were counted in both categories

AE: adverse event; BDI-II: Beck Depression Inventory-II; HADS: Hospital Anxiety and Depression Scale; HRQOL: health-related quality of life; NA: not applicable; PedsQL FIM: Paediatric Quality of Life Inventory Family Impact Module; PRO: patient-reported outcome; QOL: quality of life; SD: standard deviation; TAND: tuberous sclerosis complex-associated neuropsychiatric disorders; TSC: tuberous sclerosis complex

### Costs, resource use and treatment patterns

**Fig. 6:**
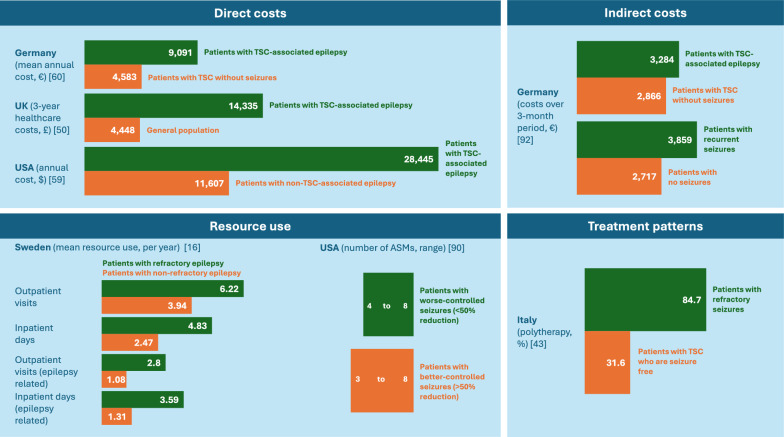
Summary of direct costs, indirect costs, resource use and treatment patterns: comparisons between patients with TSC-associated epilepsy and comparator groups. ASM: antiseizure medication; TSC: tuberous sclerosis complex

This review found 93 articles on costs and resource use, and 59 on treatment patterns in TSC; these were grouped by type of data (direct costs and resource use, indirect costs and treatment patterns) and by geographical location. Figure 6 summarises the comparative cost and resource use results.

#### Direct costs and resource use

Healthcare costs for patients with TSC-associated epilepsy were substantial (Additional file 2: Table 3). For example, in the UK, average 3-year healthcare costs were over three times higher for patients with TSC-associated epilepsy than for the general population (£14,335 vs £4,448), mainly because of inpatient admissions [50]. In Germany, total costs were higher for patients with TSC-associated epilepsy (€9,091) than for patients with TSC without seizures (€4,583) [60]. Based on US health insurance claims data, the total costs for TSC-associated seizures were $5,927–$9,174 per patient per year [89], although separately reported data also demonstrate epilepsy-related medical charges were significantly higher in the USA for patients with TSC-associated epilepsy ($28,445) than for those with non-TSC-associated epilepsy ($11,607) [59].

Contributing to high healthcare costs, adults and children with TSC-associated epilepsy incurred frequent hospital admissions and outpatient appointments. In the UK, the number of elective hospital admissions for patients with TSC-associated epilepsy was 2.0 per patient over 3 years with an average length of stay of 4.3 days, with shorter stays for children compared with adults [50]. Another study reported that patients in the USA with TSC-associated epilepsy and ≥ 1 medically treated seizure over 2 years attended emergency departments 1.3–1.7 times, physician visits 8.4–13.3 times and hospital outpatient visits 4.7–5.3 times per year [89]. Patients in Sweden with TSC-associated epilepsy spent an average of 3.25 days per year in hospital, of which 2.06 days were related directly to epilepsy [16].

Seizure control may affect healthcare resource use. For example, patients in Sweden with refractory seizures used more inpatient and outpatient resources compared with patients with better-controlled seizures [16], and those in the USA with worse-controlled seizures required more ASMs than patients who were seizure free or who had better-controlled seizures [90]. Seizure type and additional symptoms or manifestations also differentially affect resource use. In Sweden, resource use was reported to be higher for patients with TSC-associated epilepsy who had infantile spasms than those who experienced focal or non-focal seizures [16]. Patients in the USA with concomitant neurological conditions, West syndrome or infantile spasms, or a developmental disorder in addition to TSC-associated epilepsy, required more resources than patients without these additional conditions [91]. A UK study also demonstrated most patients (83.7%) with TSC-associated epilepsy had at least one other manifestation, with additional manifestations substantially increasing 3-year costs over those for epilepsy alone (£5,053): one manifestation (+ £8,333); two manifestations (+ £12,035); three manifestations (+ 17,876); four manifestations (+ £24,901) [50].

#### Indirect costs

Indirect costs have been demonstrated to be higher in patients with TSC-associated epilepsy than those with TSC without seizures, and in those with recurrent seizures compared with patients who did not have seizures [92]. A retrospective survey in France and a retrospective chart review in the Netherlands demonstrated the negative impact of TSC-associated epilepsy on patients’ ability to live independently and receive pay above the income threshold, even in stable employment [83, 93]. Internationally, approximately half of adult patients with TSC (60% of whom had epilepsy) and caregivers, and a quarter of other family members, reported a negative impact of TSC on their career or education [94]. Importantly, the indirect financial burden associated with TSC-related epilepsy substantially impacts caregivers, as highlighted in most of the 15 identified articles (Additional file 2**: **Table S4). In the UK, 56% of caregivers of patients with TSC (95% with a history of seizures) reported receiving no financial support, 45% had to give up work completely and 51% reduced their working hours because of the burden of care, while 23% of partners of caregivers reduced their working hours and 9% gave up work completely [95]. Data on caregivers of paediatric patients from a 5-year follow-up of the international TuberOus SClerosis registry to increase disease Awareness (TOSCA), in which 85% of adult and paediatric patients had epilepsy, showed that 34.1% of caregivers were unemployed and 56.8% of caregivers’ professional careers had been impacted [5]. The same study found that unemployment was greater for adult patients (58.2%) than caregivers (34.1%); however, a similar proportion of adult patients and caregivers reported an impact of TSC on their career (50.9% vs 56.8%) [5]. Another study found that caregivers have a higher productivity loss (65 years of potential employment lost) compared with patients with TSC-associated epilepsy (47 years for childhood-onset and 30 years for adult-onset epilepsy) [96].

#### Treatment patterns

Additional File 2: Table S5 shows treatment patterns for subpopulations of patients with TSC-associated epilepsy from 59 studies. The most commonly used drug for TSC-associated epilepsy was vigabatrin in Europe followed by levetiracetam, lamotrigine and oxcarbazepine [16, 43, 72, 97–100], and valproate in the USA followed by lamotrigine, levetiracetam and vigabatrin [19, 68, 91].

As only 20% to 38% of patients receiving ASMs took one ASM [101–103], it can be deduced that patients in Europe frequently take multiple ASMs, reflecting the refractory nature of TSC-associated epilepsy: the proportion of patients receiving two ASMs ranged from 15 to 45%, and 10% to 32% received three ASMs [60, 101–103]. This was also reflected in a study specific to the UK, with 31.6% receiving two ASMs, 22.0% receiving three ASMs and 6.2% receiving four or more ASMs [50].

Several studies reported greater polypharmacy (where the number of ASMs qualifying as ‘polypharmacy’ was not defined) in patients with refractory epilepsy (up to 11.7 ASMs) compared with patients with a low seizure frequency or no seizures (range 1.6 to 4.5 ASMs) [45, 90, 93, 104, 105]. For example, one study found that most patients who were seizure free could be managed with monotherapy (56.1%), with 31.6% receiving polytherapy; however, patients with refractory TSC-associated epilepsy were more likely to receive polytherapy (84.7% vs 15.3% on monotherapy) [43].

In line with indications for ASMs, we found that vigabatrin use depends on seizure type, according to the findings of two studies that compared the treatment patterns in patients with infantile spasms and patients with other seizure types. The first study reported that vigabatrin was used as a first-line ASM in 96.0% of patients with TSC and infantile spasms, but was only used in 30.0% of patients with TSC and other seizure types [99]. In the second study, which assessed historical treatment patterns in patients with TSC-associated epilepsy in the TOSCA registry up to 2015, use of vigabatrin increased over time to 91.2% in the period 2005–2010 for patients with infantile spasms, at which point 18.2% had refractory epilepsy; for patients with focal seizures, vigabatrin use increased to 76.9% in the period 2005–2010, when 42.0% had refractory epilepsy [100]. The study also reported similar rates of polypharmacy for patients with infantile spasms (22.5%) and those with focal seizures (19.1%) [100].

Everolimus use was reported by 9.0–32.5% of mixed-age patients [57, 105]. As may be expected, everolimus usage is higher in patients receiving ASM polytherapy compared with monotherapy [105]. Despite this, everolimus is used infrequently with cannabidiol [59], though cannabidiol is used in 9.0–10.0% of patients with TSC-associated epilepsy who had previously received everolimus [106].

## Conclusions

This SLR found that the burden of TSC-associated epilepsy was profound—impacting patients, caregivers, other family members, healthcare service providers and wider society. The unexpected lower prevalence of TSC relative to incidence [24–27] likely reflects the lack of studies reporting both measures and differences in the study populations reporting each measure. Furthermore, because these studies provide estimates of TSC prevalence in different populations at different times, it is difficult to make direct comparisons or generalise findings across studies.

We calculated a mean prevalence of TSC-associated epilepsy of 79.8% and 64.1% in children and adult populations, respectively, with wide ranges reported for refractory epilepsy [38–41] and seizure freedom [44, 45]. It is unclear whether the prevalence of TSC-associated epilepsy has changed in children and adults over time; while the diagnosis and incidence of TSC-associated epilepsy may have changed, it is also possible the respective decrease and increase in prevalence reported reflects methodological differences between studies.

Patients with TSC-associated epilepsy had higher mortality than those with TSC without seizures [60], demonstrating that epilepsy is an additional cause of death along with non-epilepsy-related causes [12, 16, 70]. Symptoms such as speech/language and cognitive problems were common in patients with TSC-associated epilepsy. Prevalence of many behavioural, scholastic and neuropsychological TAND categories was significantly higher in patients with active TSC-associated epilepsy versus those with seizure freedom or no history of seizures [74]. As mentioned, seizures experienced in patients with TSC typically develop in the first years of life and have an associated risk of ASD and cognitive impairment if uncontrolled during infancy [2, 17–19]. While the behavioural features of TAND can be attributed to co-occurring ASD and TSC-associated epilepsy [74], the impact on scholastic and neuropsychological categories are likely to reflect the damaging impact of infantile spasms and early seizure onset on cognition [2, 17–19], and support guidance regarding early recognition and treatment of both seizures and TAND (references either not captured within the inclusion criteria of this SLR or published since the cut-off date of this SLR) [2, 107, 108].

Seizure management could reduce the burden of frequent seizures on daily life for patients, caregivers, siblings and the wider family, thus improving QOL [22, 56, 80]. However, there was inconsistency between studies, and it was difficult to compare studies that used different PROs for the same measures. This suggests that standardised measures would be beneficial in assessing the BOI of TSC-associated epilepsy.

There was a high economic burden and unmet need related to patients with TSC-associated epilepsy, who incurred substantial healthcare costs with frequent outpatient and primary care visits and hospital admissions. Patients with TSC-associated epilepsy had higher healthcare resource use than patients with TSC without seizures [16] or with non-TSC-associated epilepsy [59]. Underlying reasons for these differences likely stem from the higher inpatient and outpatient admissions in patients with refractory seizures [16] and the higher epilepsy-related medical charges and ASM costs in patients with TSC-associated versus non-TSC-associated epilepsy [59]. Also indicative of the anticipated impact of refractory epilepsy, patients with TSC-associated epilepsy, along with their caregivers, reported impaired attendance and productivity at school or work [5, 94, 95]. Together, these findings suggest that earlier diagnosis and more effective treatments are needed to mitigate the costly impact of refractory epilepsy [2, 16, 18, 47, 93, 105].

Similar to the findings with TAND, there was evidence that higher seizure frequencies were associated with worse cognitive function [18] and greater caregiver burden [88], so initiating treatment early to reduce seizure frequency might prevent some intellectual impairment and improve caregivers’ QOL. This was demonstrated in the EPISTOP study (not captured within the inclusion criteria of this SLR) [109], in which patients receiving preventative vigabatrin treatment were less likely to have neurodevelopmental delay by 2 years of age and had improved intellectual ability over patients receiving conventional treatment after the onset of seizures (both results not significant). In contrast, the PREVeNT study (published since the cut-off date of this SLR) found that, although vigabatrin initiation at first epileptiform electroencephalogram (EEG) in infants with TSC was associated with later onset time and lower incidence of infantile spasms versus placebo, patients did not demonstrate improved cognition, behaviour or incidence of focal seizures at 2 years of age [110]. However, preventing seizures may not result in children reaching their full intellectual potential, as the brain lesions that act as epileptic foci may also contribute to poor cognitive function (from a study not captured within the inclusion criteria of this SLR) [111]. It is not clear whether cognitive impairment occurs as a result of the seizures, or if these are both caused independently by the presence of brain lesions. Evidence was mixed regarding the subsequent impact of TSC-associated epilepsy on depression and anxiety [39, 41, 74]; surprisingly, higher depression has been associated with the absence of epilepsy [74] or lower cognitive impairment [48], which may suggest that these measures are difficult to determine in these patient cohorts and/or other TSC manifestations may confer a greater impact on mood—for example, sources of pain such as renal complications and LAM (references either not captured within the inclusion criteria of this SLR or published since the cut-off date of this SLR) [112, 113].

Some similarities and points of difference were identified when comparing the findings from the present SLR with that of Zöllner et al. (2020), which can be expected given the focus here on TSC-associated epilepsy and the later data cut resulting in inclusion of additional and more recent articles (241 vs 33 in Zöllner et al.) [1]. The present review reported lower mean prevalence of epilepsy (62.5% in mixed-age populations, 64.1% in adults and 79.8% in children vs 83.5–88.4% in mixed-age populations), but wider ranges for prevalence of TAND, including intellectual impairment (8.0–90.0% vs 53.6–65.0%) and ASD (4.9–70.0% vs 25.0–61.0%), potentially because of the discrepancies in the numbers of articles identified [1].

Both the Zöllner et al. (2020) review and the present SLR identified that many PRO tools are used to assess QOL in both patients and caregivers, and that data on the impact of TSC-associated epilepsy on caregivers’ QOL are sparse; Zöllner et al. suggested that standardised questionnaires should be encouraged in favour of using several different PRO tools to assess BOI [1]. Indeed, a post-hoc analysis of the most common PROs used to assess QOL across patient and caregiver populations in Tables 1, 2 and Additional file 2: Table S2 found questionnaires/surveys/interviews to be one of the most common methods to obtain PROs (n = 5), and Vineland Adaptive Behavior Scales (VABS) (n = 6), Mullen Scales of Early Learning (MSEL) (n = 5) and QOLCE (n = 5) to be the most common PRO tools. Future studies could benefit from aligning on these common PROs to assess QOL. Both reviews also identified higher direct costs and resource use in patients with TSC compared with the healthy population [1]. Additionally, we highlight herein that patients with TSC-associated epilepsy incurred higher direct costs and resource use compared with patients with TSC without seizures. Patients with TSC-associated epilepsy also have high indirect costs, such as caregiver productivity loss, compared with individuals who do not have TSC-associated epilepsy. A survey on the use of PROs for patients with epilepsy published since the cut-off date of this SLR noted a consensus on the positive impact that PROs can have on clinical outcomes, healthcare resource utilisation and general patient satisfaction, in addition to indicating the value of an intervention; all respondents felt that PROs would play a bigger role over time [114].

There are several limitations of this SLR. Some of these are based on the lack of information we found on particular aspects of the disease. Firstly, there are a lack of PRO data in adults and mixed-age populations assessing QOL in the absence of an intervention additional to the patient’s current treatment. Only four of the studies identified reported prevalence data after 2010, and the most recent prevalence data were from 2016; proportions of children and adults with TSC-associated epilepsy cannot be compared because adult patients were likely diagnosed before the introduction of genetic testing for TSC. As also identified by Zöllner et al. (2020), there is no standardised assessment of BOI, and there is a myriad of different QOL measures [1]. There is also a lack of detailed information on how TSC-associated epilepsy impacts families, even though it is an autosomal dominant disorder that can affect more than one family member simultaneously [61, 115]. There were few randomised controlled trials (RCTs) on ASMs in patients with TSC-associated epilepsy, so there are limited robust data on which to base clinical decisions about treatments. In terms of the search strategy itself, articles included in the epidemiology search had to be in English; this may mean that some countries and regions were underrepresented. The Cochrane risk-of-bias 2 tool found that > 50% of the non-RCT efficacy studies had some overall risk of bias; however, this is not surprising given that non-RCTs are inherently at greater risk of bias than RCTs. As no formal statistical analyses were conducted, we were not able to estimate publication bias, conduct sensitivity analyses or assess outcome evidence certainty. Lastly, this review will not have captured any studies published in the time elapsed since the last search (26 April 2023).

This review provides an overview of the BOI of TSC-associated epilepsy, including the impact on QOL and cost and resource use. Considering the already high burden of disease in the TSC population, the burden of TSC-associated epilepsy is substantial compared with the general population. The epidemiological findings provide context for the impact of the BOI and the need for continued innovation to derive novel biomarkers for epileptogenesis to support earlier diagnosis, develop improved and targeted treatments or better understand combinatorial treatment strategies to engage different targets in the epileptogenic network, and for continued adherence to the management recommendations, particularly those seeking to address the damaging impacts of uncontrolled seizures during infancy [2, 107, 108]. The development of standardised measures would be beneficial in demonstrating this impact, and future studies could build on the EPISTOP and PREVeNT studies [109, 110], which suggested improved neurological outcomes for patients who received preventative treatment with vigabatrin. By improving the management of TSC-associated epilepsy, it is likely that the burden experienced by caregivers and families can also be positively impacted.

## Supplementary Information

Additional file1

Additional file2

## Data Availability

The protocol and raw datasets analysed during the SLR are available from the corresponding author at request.

## Abbreviations

**ADHD**: Attention deficit hyperactivity disorder
**ADL**: Activities of daily living
**ADOS**: Autism diagnostic observation scale
**AE**: Adverse event
**AOSI**: Autism observation scale for infants
**ASD**: Autism spectrum disorder
**ASM**: Antiseizure medication
**BDI-II**: Beck Depression Inventory-II
**BOI**: Burden of illness
**CBCL**: Child behaviours check list
**CBD**: Cannabidiol
**CI**: Confidence interval
**DISABKIDS**: Quality of life assessment in children with chronic health conditions and disabilities
**EA**: Epileptiform activity
**EEG**: Electroencephalography
**EVE**: Everolimus
**GARS**: Gilliam autism rating scale
**GEOS**: Glasgow epilepsy outcome scale
**HADS**: Hospital anxiety and depression scale
**HC**: Healthy control (e.g. general population)
**HRQOL**: Health-related quality of life
**ID**: Intellectual disability
**IOFS**: Impact-on-family scale
**IQ**: Intelligence quotient
**IQR**: Interquartile range
**IS**: Infantile spasm
**LAEP**: Liverpool adverse event profile
**LAM**: Lymphangioleiomyomatosis
**MSEL**: Mullen scales of early learning
**NA**: Not applicable
**NCBRF**: Nisonger child behavior rating form
**OABC**: Overall adaptive behaviour composite
**OR**: Odds ratio
**PedsQL**: Paediatric quality of life inventory
**PedsQL 4.0**: Paediatric quality of life inventory version 4.0
**PedsQL FIM**: Paediatric quality of life inventory family impact module
**PLS-5**: Pre-school language scale, fifth edition
**PORS**: Partial-onset refractory seizures
**PRISMA**: Preferred reporting items for systematic reviews and meta-analyses
**PRO**: Patient-reported outcome
**PSI**: Parental stress index
**QOL**: Quality of life
**QOLCE**: Quality of life for children with epilepsy
**QOLIE**: Quality of life in epilepsy inventory
**QOLIE-31**: 31-Item quality of life in epilepsy inventory
**QOLIE-31-P**: 31-Item quality of life in epilepsy inventory–problems
**QOLIE-AD-48**: 48-Item quality of life in epilepsy inventory for adolescents
**RCT**: Randomised controlled trial
**SCQ**: Social communication questionnaire
**SD**: Standard deviation
**SDQ-D**: Strengths and difficulties questionnaire (German version)
**SEGA**: Subependymal giant cell astrocytoma
**SLR**: Systematic literature review
**SRS**: Social rating scale
**SRS-2**: Social responsiveness scale
**SUDEP**: Sudden unexpected death in epilepsy
**TACERN**: TSC autism center of excellence network
**TAND**: Tuberous sclerosis complex-associated neuropsychiatric disorders
**TOSCA**: TuberOus SClerosis registry to increase disease Awareness
**TSC**: Tuberous sclerosis complex
**TSC1**: TSC subunit 1
**TSC2**: TSC subunit 2
**VABS**: Vineland adaptive behaviour scale
**VABS-II**: VABS, second edition
**WIS-CR**: Wechsler intelligence scale (Chinese revision)
**WNV**: Wechsler non-verbal
