# Supplementary material for: Burden of illness in tuberous sclerosis complex-associated epilepsy: a systematic literature review of epidemiology, health-related quality of life, costs and resource use
Source: Orphanet J Rare Dis. 2025 Nov 25;20:609. doi: 10.1186/s13023-025-03975-y (PMC12648938; doi:10.1186/s13023-025-03975-y)
Supplement: Supplementary file 1 — Additional file1 [file 13023_2025_3975_MOESM1_ESM.docx]

# Additional online material for:

**Burden of illness in tuberous sclerosis complex-associated epilepsy: a systematic literature review of epidemiology, health-related quality of life, costs and resource use**

**Data collection and synthesis process**

- All types of data for each of the following outcomes were extracted from included papers into an Excel template by one researcher; a second researcher checked all extracted data for accuracy, relevance and missing data
  - Baseline characteristics: age, sex, race/ethnicity, genetic abnormalities, co-occurring symptoms, seizure types, seizure frequency, previous treatments, developmental level, intelligence level, educational ability, work ability
  - Epidemiology outcomes: incidence/prevalence of tuberous sclerosis complex (TSC) within a wider population, diagnosis of TSC, epilepsy risk (within a population of TSC), features/manifestations of TSC, mortality, prognosis, guideline recommendations
  - Quality of life outcomes: health state utility measurements (e.g. EuroQol 5 Dimensions (EQ-5D), Health Utility Index, 12- or 36-Item Short Form Health Survey, time trade-off), epilepsy-specific quality of life scores (e.g. Quality of Life in Epilepsy Inventory [QOLIE], Quality of Life for Children with Epilepsy [QOLCE]), other patient-reported outcomes (PROs; e.g. TSC-associated neuropsychiatric disorders [TAND], Vineland Adaptive Behaviour Scale, Paediatric Quality of Life Inventory [PedsQL], Epworth Sleepiness Scale, Bayley Scales of Infant Development)
  - Healthcare cost and resource use outcomes: antiseizure medication treatment patterns, number of hospital admissions, length of hospital admissions, number of healthcare consultations, number of emergency department visits, direct costs of medication, direct costs of hospital admissions, other direct costs, indirect costs from reduced parental or patient productivity, and other indirect costs
- Due to the volume and diversity of data, the author group decided to focus the manuscript on outcomes related to TSC-associated epilepsy resulting in some studies not being reported in the manuscript despite meeting the original inclusion criteria that did not report information in this population or did not specify a high percentage of the population with TSC-associated epilepsy
  - This also applied to studies not deemed suitable for the calculation of epilepsy risk (eg data were repeated in two different publications)
- Authors of included papers were not contacted for additional data
- Data from charts were digitised using webplotdigitizer (<https://automeris.io/WebPlotDigitizer/>), but no other automation was used to assist with data extraction
- No formal statistical analyses were conducted on the data
  - Epidemiological data were calculated as simple means and median values by dividing the number of patients with each feature by the total number of patients assessed, across all relevant studies; no assessments of certainty such as 95% confidence intervals were calculated for these outcomes, no assumptions were made about missing data, and only simple data conversions were conducted to facilitate comparisons; for example, converting absolute numbers to proportions (or vice versa)

# Online additional table S1. Full inclusion and exclusion criteria used for abstracts and full text screening

| **Criterion** | **Inclusion criteria** | **Exclusion criteria** |
| --- | --- | --- |
| Population and disease | Eligible studies in adults or children reported seizures associated with TSC, apart from those on cost and QOL, which could report general TSC with or without seizures^†^ | Non-neurological manifestations of TSC were excluded, as were studies with mixed populations where <80% of patients had TSC |
| Intervention | Any or none |  |
| Comparators | Any or none |  |
| Outcomes | Epidemiology, QOL, economic evaluations, cost and resource use, impact on work and productivity, education and learning | Genetic studies, efficacy and safety studies |
| Study type | Primary research, systematic reviews, narrative reviews |  |
| Language | Epidemiology studies: English only; QOL and costs studies: any language |  |
| Study size | >100 patients for epidemiology studies; >5 patients for other topics |  |
| Date | Any |  |

^†^While articles reporting general TSC with or without seizures were included at full text screening, some articles were later removed to focus the SLR on TSC-associated epilepsy (see **Figure 1**)

QOL: quality of life; SLR: systematic literature review; TSC: tuberous sclerosis complex

# Online additional table S2. Search strategies

| **Database** | **#** | **Search terms** | **Number of hits (26 April 2023)** |
| --- | --- | --- | --- |
| **EMBASE** | **#1** | ('tuberous sclerosis'/exp OR 'tuberous sclerosis' OR 'tuberous sclerosis':ab,ti OR tsc:ab,ti) AND [embase]/lim AND [humans]/lim AND [abstracts]/lim | 10,980 |
|  | **#2** | ('epilepsy'/exp OR 'epilepsy' OR 'seizure'/exp OR 'seizure' OR 'generalized epilepsy' OR 'focal epilepsy' OR epilepsy:ab,ti OR epilept*:ab,ti OR seizure*:ab,ti OR convuls*:ab,ti OR fit*:ab,ti OR spasm*:ab,ti OR attack*:ab,ti OR atonic:ab,ti OR myoclonic:ab,ti OR drop:ab,ti OR tonic:ab,ti OR clonic:ab,ti OR partial:ab,ti OR focal:ab,ti OR absence:ab,ti) AND [embase]/lim AND [abstracts]/lim AND [humans]/lim | 1,538,493 |
|  | **#3** | #1 AND #2 | 8,666 |
|  | **#4** | 'quality of life'/exp OR 'quality of life' OR 'quality of life':ab,ti OR qol:ab,ti OR hqol:ab,ti OR hrqol:ab,ti OR hql:ab,ti OR hrql:ab,ti OR utilit*:ab,ti OR 'patient reported':ab,ti OR 'patient-reported':ab,ti OR euroqol:ab,ti OR 'eq 5d':ab,ti OR eq5d:ab,ti OR 'eq vas':ab,ti OR 'health utilit* index':ab,ti OR hui:ab,ti OR hui2:ab,ti OR hui3:ab,ti OR 'hui 2':ab,ti OR 'hui 3':ab,ti OR (('short form':ab,ti OR 'short-form':ab,ti OR shortform:ab,ti OR sf:ab,ti) AND (6:ab,ti OR 12:ab,ti OR 36:ab,ti)) OR 'time trade off':ab,ti OR 'time trade-off':ab,ti OR tto:ab,ti OR 'standard gamble':ab,ti OR 'patient preference':ab,ti OR 'self-reported':ab,ti OR 'outcome assessment':ab,ti | 959,575 |
|  | **#5** | ('economic evaluation'/exp OR 'economic evaluation' OR 'cost of illness'/exp OR 'cost of illness' OR cost*:ab,ti OR budget*:ab,ti OR financ*:ab,ti OR resource*:ab,ti OR 'resource use':ab,ti OR 'length of stay':ab,ti OR admission*:ab,ti OR economic*:ab,ti OR hospitali?ation:ab,ti OR absenteeism:ab,ti OR productivity:ab,ti OR ((value NEAR/1 (money OR monetary)):ab,ti)) | 1,621,512 |
|  | **#6** | #4 OR #5 | 2,399,360 |
|  | **#7** | #1 AND #6 | 983 |
|  | **#8** | #3 OR #7 | 8,856 |
| **Medline and PubMed-not-Medline (via Embase.com)** | **#1** | "tuberous sclerosis"[MeSH] OR "Tuberous sclerosis"[Title/Abstract] OR TSC[Title/Abstract] | 12,587 |
|  | **#2** | epilepsy[MeSH Terms] OR epilepsy OR seizure OR "generalized epilepsy" OR seizure[MeSH Terms] OR "focal epilepsy" OR epilepsy[Title/Abstract] OR epilept*[Title/Abstract] OR seizure*[Title/Abstract] OR convuls*[Title/Abstract] OR fit[Title/Abstract] OR fits[Title/Abstract] OR fitting[Title/Abstract] OR spasm*[Title/Abstract] OR attack*[Title/Abstract] OR atonic[Title/Abstract] OR myoclonic[Title/Abstract] OR drop[Title/Abstract] OR tonic[Title/Abstract] OR clonic[Title/Abstract] OR partial[Title/Abstract] OR focal[Title/Abstract] OR absence[Title/Abstract] | 2,037,383 |
|  | **#3** | #1 AND #2 | 3,302 |
|  | **#4** | "quality of life"[MeSH Terms] OR "quality of life"[Title/Abstract] OR qol[Title/Abstract] OR hqol[Title/Abstract] OR hrqol[Title/Abstract] OR hql[Title/Abstract] OR hrql[Title/Abstract] OR utilit*[Title/Abstract] OR "patient reported"[Title/Abstract] OR "patient-reported"[Title/Abstract] OR euroqol[Title/Abstract] OR eq 5d[Title/Abstract] OR eq5d[Title/Abstract] OR "eq vas"[Title/Abstract] OR "health utilit*"[Title/Abstract] OR hui[Title/Abstract] OR hui2[Title/Abstract] OR hui3[Title/Abstract] OR "time trade off"[Title/Abstract] OR "time trade-off"[Title/Abstract] OR tto[Title/Abstract] OR "standard gamble"[Title/Abstract] OR "patient preference"[Title/Abstract] OR "self-reported"[Title/Abstract] OR "outcome assessment"[Title/Abstract] | 845,390 |
|  | **#5** | ("short form"[Title/Abstract] OR "short-form"[Title/Abstract] OR shortform[Title/Abstract] OR sf[Title/Abstract]) AND (6[Title/Abstract] OR 12[Title/Abstract] OR 36[Title/Abstract]) | 55,967 |
|  | **#6** | #4 OR #5 | 866,537 |
|  | **#7** | "economic evaluation"[Title/Abstract] OR "Cost of Illness"[MeSH] OR "cost of illness"[Title/Abstract] OR cost*[Title/Abstract] OR budget*[Title/Abstract] OR financ*[Title/Abstract] OR resource*[Title/Abstract] OR "length of stay"[Title/Abstract] OR admission*[Title/Abstract] OR economic*[Title/Abstract] OR hospitali?ation*[Title/Abstract] OR absenteeism[Title/Abstract] OR productivity[Title/Abstract] OR (value[Title/Abstract] AND money[Title/Abstract]) OR monetary[Title/Abstract] | 1,900,387 |
|  | **#8** | #6 OR #7 | 2,637,693 |
|  | **#9** | #1 AND #8 | 492 |
|  | **#10** | #3 OR #9 | 3,591 |
|  | **#11** | #10 Filters applied: Abstract, Humans | 2,607 |
| **Cochrane library** | **#1** | MeSH descriptor: [Tuberous Sclerosis] explode all trees | 131 |
|  | **#2** | tuberous sclerosis OR TSC | 772 |
|  | **#3** | #1 OR #2 | 772 |
| **Heoro.com** | **#1** | Disease: Tuberous Sclerosis | 21 |
| **NICE** | **#1** | Tuberous sclerosis | 3 |
| **SMC** | **#1** | Tuberous Sclerosis | 3 |
| **PBAC** | **#1** | Tuberous Sclerosis | 0 |
| **CADTH** | **#1** | Tuberous sclerosis | 2 |
| **NCPE** | **#1** | Tuberous Sclerosis | 0 |
| **ISPOR** | **#1** | Search site: Disease/disorder: neurological disorders  Keywords: "tuberous sclerosis"  Topic: all  Conference: all | 26 |
| **AAN** | **#1** | Tuberous sclerosis | 7 |
| **AES** | **#1** | Tuberous sclerosis | 131 |
| **WCN** | **#1** | Downloaded abstract book, hand searched | 26 |
| **AWMSG** | **#1** | Tuberous Sclerosis | 2 |
| **ClinicalTrials.gov** | **#1** | Tuberous sclerosis | 98 |
| **York CRD** | **#1** | Tuberous sclerosis OR TSC | 7 |
| **International Epilepsy Congress** | **#1** | Downloaded booklets, hand searched | 23 |
| **European Congress on Epilepsy** | **#1** | Downloaded booklets, hand searched | 28 |
| **ScHARRHUD** | **#1** | Unavailable | 0 |
| **EuroQol** | **#1** | Tuberous sclerosis | 0 |
| **ANSM** | **#1** | Tuberous | 1 |
| **International TSC Research Conference** | **#1** | Downloaded booklets, hand searched | 33 |

AAN: American Academy of Neurology; AES: American Epilepsy Society; ANSM: Agence nationale de sécurité du médicament et des produits de santé (the French National Agency for the Safety of Medicines and Health Products); AWMSG: All Wales Medicines Strategy Group; CADTH: Canadian Agency for Drugs and Technologies in Health; CRD: Centre for Reviews and Dissemination; EMBASE: Excerpta Medica dataBASE; EuroQol: European Quality of Life Database; ISPOR: International Society for Pharmacoeconomics and Outcomes Research (conference abstracts 2015–2018); NCPE: National Centre for Pharmacoeconomics, Ireland; NICE: National Institute for Health and Care Excellence; PBAC: Pharmaceutical Benefits Advisory Committee, Australia; ScHARRHUD: Sheffield Centre for Health and Related Research Health Utilities Database; SMC: Scottish Medicines Consortium; TSC: tuberous sclerosis complex; WCN: World Congress of Neurology (2017 and 2019 congresses); York CRD: University of York Centre for Reviews and Dissemination (NHS Economic Evaluation Database 1968 to April 2015 and HTA search)

# Online additional table S3. Additional epidemiology search strategy

| **Database** | **#** | **Search terms** | **Number of hits (26 April 2023)** |
| --- | --- | --- | --- |
| **PubMed** | **#1** | "tuberous sclerosis"[MeSH] OR "Tuberous sclerosis"[Title/Abstract] OR TSC[Title/Abstract] | 12,587 |
|  | **#2** | Incidence[MeSH] OR incidence[Title/Abstract] OR Prevalence[MeSH] OR prevalence[Title/Abstract] OR Diagnosis[MeSH] OR diagnosis[Title/Abstract] OR diagnoses[Title/Abstract] | 11,325,750 |
|  | **#3** | #1 AND #2 | 6,001 |
|  | **#4** | database*[Title/Abstract] OR Registries[MeSH] OR register*[Title/Abstract] OR registr*[Title/Abstract] OR national[Title/Abstract] OR international[Title/Abstract] OR "Cohort Studies"[MeSH] OR "cohort analysis"[Title/Abstract:~2] OR "cohort analyses"[Title/Abstract:~2] OR "cohorts analysis"[Title/Abstract:~2] OR "cohorts analyses"[Title/Abstract:~2] | 4,089,822 |
|  | **#5** | #3 AND #4 | 1,158 |
|  | **#6** | #5 Filters applied: Abstract, Humans | 1,074 |
| **EMBASE** | **#1** | ('tuberous sclerosis'/exp OR 'tuberous sclerosis':ab,ti OR tsc:ab,ti) AND [humans]/lim AND [abstracts]/lim | 12,076 |
|  | **#2** | ('incidence'/exp OR 'incidence':ab,ti OR 'prevalence'/exp OR 'prevalence':ab,ti OR 'diagnosis'/exp OR 'diagnosis':ab,ti) AND ([humans]/lim AND [abstracts]/lim) | 7,565,389 |
|  | **#3** | #1 AND #2 | 6,914 |
|  | **#4** | (database:ab,ti OR 'register'/exp OR 'register':ab,ti OR registry:ab,ti OR 'disease registry'/exp OR 'disease registry':ab,ti OR national:ab,ti OR international:ab,ti OR 'cohort analysis'/exp OR 'cohort analysis':ab,ti) AND ([humans]/lim AND [abstracts]/lim) | 2,571,490 |
|  | **#5** | #3 AND #4 | 744 |

EMBASE: Excerpta Medica dataBASE; TSC: tuberous sclerosis complex

# Online additional table S4. Studies excluded after full text screening and entries excluded because of repeated data, lack of suitability for calculation of epilepsy risk, or to focus on TSC-associated epilepsy

| **Citation** | **Reason for exclusion** | | | | | | |
| --- | --- | --- | --- | --- | --- | --- | --- |
|  | Duplicate | Language | No relevant data | Population | Population not generalisable^†^ | Secondary publication with no additional data | Size |
| **Studies excluded after full text screening** |  |  |  |  |  |  |  |
| Agata 1999 [1] |  | x |  |  |  |  |  |
| Aicardi 1996 [2] |  |  |  | x |  |  |  |
| Alsowat 2021 [3] |  |  |  | x |  |  |  |
| Amin 2018 [4] |  |  | x |  |  |  |  |
| Amin 2015 [5] |  |  | x |  |  |  |  |
| Aungaroon 2022 [6] |  |  | x |  |  |  |  |
| Barzegar 2021 [7] |  |  |  | x |  |  |  |
| Caban 2016 [8] |  |  | x |  |  |  |  |
| Callaghan 2018 [9] |  |  | x |  |  |  |  |
| Cantarín-Extremera 2021 [10] |  | x |  |  |  |  |  |
| Capal 2017 [11] |  |  | x |  |  |  |  |
| Cardozo 2019 [12] |  |  | x |  |  |  |  |
| Chin 2020 [13] |  |  | x |  |  |  |  |
| Chiron 1997 [14] |  |  |  |  | x |  |  |
| Chung 2017a [15] |  |  |  |  | x |  |  |
| ClinicalTrials.gov 2014 [16] |  |  | x |  |  |  |  |
| ClinicalTrials.gov 2018a [17] |  |  | x |  |  |  |  |
| ClinicalTrials.gov 2018b [18] |  |  | x |  |  |  |  |
| ClinicalTrials.gov 2019 [19] |  |  | x |  |  |  |  |
| ClinicalTrials.gov 2022 [20] |  |  | x |  |  |  |  |
| ClinicalTrials.gov 2023a [21] | x |  |  |  |  |  |  |
| ClinicalTrials.gov 2023b [22] |  |  | x |  |  |  |  |
| Cock 2021 [23] |  |  |  |  |  | x |  |
| Cockerell 2021 [24] |  |  | x |  |  |  |  |
| Coevoets 2009 [25] |  |  | x |  |  |  |  |
| Combes 2018 [26] |  |  | x |  |  |  |  |
| Curatolo 2018 [27] |  |  |  |  | x |  |  |
| D’Onofrio 2020 [28] |  |  |  |  |  |  | x |
| De Ridder 2020 [29] |  |  |  |  |  |  | x |
| De Ridder 2021 [30] |  |  |  | x |  |  |  |
| de Vries 2020a [31] |  |  |  |  | x |  |  |
| Dorofeeva 2005 [32] |  | x |  |  |  |  |  |
| Ebrahimi-Fakhari 2020 [33] |  |  | x |  |  |  |  |
| Farges 2022 [34] |  |  | x |  |  |  |  |
| Fleury 1980 [35] |  |  | x |  |  |  |  |
| Franco 2021 [36] |  |  | x |  |  |  |  |
| Franz 2015 [37] |  |  |  |  |  | x |  |
| Franz 2017 [38] | x |  |  |  |  |  |  |
| García-Martín 2021 [39] |  |  |  | x |  |  |  |
| Georgieva 2023 [40] |  |  |  | x |  |  |  |
| Giacaman 2017 [41] |  |  |  |  |  | x |  |
| Golub 2021 [42] |  |  | x |  |  |  |  |
| Gosnell 2021 [43] |  |  |  | x |  |  |  |
| Grigorieva 2018 [44] |  |  | x |  |  |  |  |
| Gupta 2020 [45] |  |  |  |  |  | x |  |
| Hallett 2011 [46] |  |  | x |  |  |  |  |
| Hamer 2018 [47] |  |  |  |  | x |  |  |
| Hertzberg 2012 [48] |  |  | x |  |  |  |  |
| Hirata 2020 [49] |  |  |  | x |  |  |  |
| Hulshof 2021 [50] |  |  |  | x |  |  |  |
| Jeong 2016 [51] |  |  |  |  |  | x |  |
| Joshi 2022 [52] |  |  | x |  |  |  |  |
| Jóźwiak 2017 [53] |  |  | x |  |  |  |  |
| Jozwiak 2019 [54] |  |  | x |  |  |  |  |
| Jozwiak 1998 [55] |  |  |  |  |  | x |  |
| Jóźwiak 2000 [56] |  |  |  |  |  | x |  |
| Kafle 2014 [57] |  |  |  | x |  |  |  |
| Kingswood 2015 [58] |  |  |  |  |  | x |  |
| Kotulska 2021 [59] | x |  |  |  |  |  |  |
| Kwan 2021 [60] |  |  |  |  |  | x |  |
| La Briola 2013 [61] |  | x |  |  |  |  |  |
| Landazuri 2020 [62] |  |  |  | x |  |  |  |
| Lo 2022 [63] |  |  |  |  |  | x |  |
| Lukas 2021 [64] |  |  |  | x |  |  |  |
| Manea 2017 [65] |  |  |  |  | x |  |  |
| Moavero 2022 [66] |  |  | x |  |  |  |  |
| Nabavi Nouri 2022 [67] |  |  | x |  |  |  |  |
| Neal 2021 [68] |  |  |  | x |  |  |  |
| Nickels 2017 [69] |  |  | x |  |  |  |  |
| Pack 2019 [70] |  |  | x |  |  |  |  |
| Pfirmann 2020 [71] |  |  |  | x |  |  |  |
| Raucci 1994 [72] |  | x |  |  |  |  |  |
| Riikonen 2001 [73] |  |  |  | x |  |  |  |
| Roach 1999 [74] |  |  |  |  |  | x |  |
| Rovira 2014 [75] |  |  | x |  |  |  |  |
| Sahebkar 2020 [76] |  |  |  |  |  | x |  |
| Samueli 2018a [77] |  |  |  |  |  |  | x |
| Samueli 2018b [78] |  |  | x |  |  |  |  |
| Sanchez-Carpintero 2021 [79] |  |  |  |  |  | x |  |
| Sharma 2021 [80] |  |  | x |  |  |  |  |
| Słowińska 2019 [81] |  |  |  |  |  | x |  |
| Sparagana 2011 [82] |  |  | x |  |  |  |  |
| Sparagana 2016 [83] |  |  | x |  |  |  |  |
| Story 2019 [84] |  |  | x |  |  |  |  |
| Strzelczyk 2020 [85] |  |  |  |  |  | x |  |
| Strzelczyk 2021 [86] |  |  | x |  |  |  |  |
| Sugiyama 2009 [87] |  |  | x |  |  |  |  |
| Svarrer 2019 [88] |  |  |  |  |  |  | x |
| Tohyama 2016 [89] |  |  |  |  |  | x |  |
| Tuft 2015 [90] |  |  | x |  |  |  |  |
| Tye 2023 [91] |  |  | x |  |  |  |  |
| Wheless 2021 [92] |  |  |  |  |  | x |  |
| World Health Organization 2011 [93] |  |  | x |  |  |  |  |
| World Health Organization 2020 [94] |  |  | x |  |  |  |  |
| World Health Organization 2023 [95] | x |  |  |  |  |  |  |
| Yapici 2018 [96] |  |  |  |  |  | x |  |
| Zak 2017 [97] |  |  | x |  |  |  |  |
| Zöllner 2021 [98] |  |  | x |  |  |  |  |

^†^Studies were not generalisable and were therefore not appropriate for inclusion in the epilepsy risk analysis because they either included a population at higher risk of epilepsy than the general population with TSC, or the population consisted entirely of patients with TSC-associated epilepsy

TSC: tuberous sclerosis complex

# References

1. Agata T. Epidemiology of tuberous sclerosis in Japan. Gann Monographs on Cancer Research. 1999. p. 27–35.

2. Aicardi J, Mumford JP, Dumas C, Wood S. Vigabatrin as initial therapy for infantile spasms: a European retrospective survey. Sabril IS Investigator and Peer Review Groups. Epilepsia. 1996;37:638–42.

3. Alsowat D, Whitney R, Hewson S, Jain P, Chan V, Kabir N, et al. The phenotypic spectrum of tuberous sclerosis complex: a Canadian cohort. Child Neurol Open. 2021;8:2329048X211012817.

4. Amin S, Kingswood C, O’Callaghan F. G304 Delphi consensus process for the UK guidelines for management and surveillance of tuberous sclerosis complex. Arch Dis Child. 2018;103:A124–A124.

5. Amin S, Calder N, Merrifield J, O’Callaghan F. PP15.2 – 2888: Causes of death in individuals with tuberous sclerosis complex. Eur J Paediatr Neurol. 2015;19:S90.

6. Aungaroon G, Franz D. Stiripentol efficacy and tolerability for drug-resistant epilepsy treatment in tuberous sclerosis complex. Epilepsia. 2022;63(S2):1–278.

7. Barzegar M, Poorshiri B, Yousefi L, Raeisi S, Bakhtiary H, Eftekhari Milani A, et al. The clinical and paraclinical manifestations of tuberous sclerosis complex in children. Acta Neurol Belg. 2022;122:385–90.

8. Caban C, Khan N, Hasbani D, Crino PB. Genetics of tuberous sclerosis complex: implications for clinical practice. Appl Clin Genet. 2016;10:1–8.

9. Callaghan M, Donnelly D, Morrison P. A complete population survey of epilepsy in tuberous sclerosis patients in Northern Ireland. Ulster Med J. 2018;87(1):54–64.

10. Cantarín-Extremera V, Bernardino-Cuesta B, Martín-Villaescusa C, Melero-Llorente J, Hernández-Martín A, Aparicio-López C, et al. [Tuberous sclerosis complex: analysis of areas of involvement, treatment progress and translation to routine clinical practice in a cohort of paediatric patients]. Rev Neurol. 2021;73:141–50.

11. Capal JK, Horn PS, Murray DS, Byars AW, Bing NM, Kent B, et al. Utility of the autism observation scale for infants in early identification of autism in tuberous sclerosis complex. Pediatr Neurol. 2017;75:80–6.

12. Cardozo L, Schwind M, Carvalho D, Dufner-Almeida L, Alegria T, Nanhoe S, et al. Neuropsychological profile of tuberous sclero-sis complex patients in Brazil. 2019 International TSC Research Conference [Internet]. 2019 International TSC Research Conference Program Book; 2019. Available from: https://online.fliphtml5.com/tosk/ghir/

13. Chin R, Mingorance A, Newell I, Ruban-Fell B, Evans J, Vyas K, et al. Treatment guidelines for five rare neurodevelopmental disorders: a targeted literature review. Eur J Neurol. 2020;27(S1):441.

14. Chiron C, Dumas C, Jambaqué I, Mumford J, Dulac O. Randomized trial comparing vigabatrin and hydrocortisone in infantile spasms due to tuberous sclerosis. Epilepsy Res. 1997;26:389–95.

15. Chung C, Lawson J, Sarkozy V, Wargon O, Kennedy S, Mowat D. The experience of initiating a multidisciplinary tuberous sclerosis complex clinic in NSW. Twin Res Hum Genet. 2017;20:446–80.

16. ClinicalTrials.gov. NCT02098759. Long-term, prospective study evaluating clinical and molecular biomarkers of epileptogenesis in a genetic model of epilepsy - tuberous sclerosis complex (EPISTOP). Updated. 04 April 2014 [Internet]. 2014 [cited 2024 Sep 27]. Available from: https://clinicaltrials.gov/study/NCT02098759

17. ClinicalTrials.gov. NCT01954693. A study of everolimus in the treatment of neurocognitive problems in tuberous sclerosis (TRON). Updated. 30 January 2018 [Internet]. 2018 [cited 2024 Sep 27]. Available from: https://clinicaltrials.gov/study/NCT01954693

18. ClinicalTrials.gov. NCT01289912. Trial of RAD001 and neurocognition in tuberous sclerosis complex (TSC) (TSC). Updated. 25 January 2018 [Internet]. 2018 [cited 2024 Sep 27]. Available from: https://clinicaltrials.gov/study/NCT01289912

19. ClinicalTrials.gov. NCT01767779. Potential EEG biomarkers and antiepileptogenic strategies for epilepsy in TSC. Updated. 11 April 2019 [Internet]. 2019 [cited 2024 Sep 27]. Available from: https://clinicaltrials.gov/study/NCT01767779

20. ClinicalTrials.gov. NCT02325505. Characterization of patients with tuberous sclerosis complex, lymphangioleiomyomatosis and angiomyolipoma. Updated. 04 August 2022 [Internet]. 2022 [cited 2024 Sep 27]. Available from: https://clinicaltrials.gov/study/NCT02325505

21. ClinicalTrials.gov. NCT04595513. Stopping TSC Onset and Progression 2: epilepsy Prevention in TSC Infants (STOP2). Updated. 21 March 2024 [Internet]. 2023 [cited 2024 Sep 27]. Available from: https://clinicaltrials.gov/study/NCT04595513

22. ClinicalTrials.gov. NCT04485104. Assessment of adjunctive cannabidiol oral solution (GWP42003-P) in children with tuberous sclerosis complex (TSC), Dravet Syndrome (DS), or Lennox-Gastaut Syndrome (LGS) who experience inadequately-controlled seizures. Updated. 05 April 2023 [Internet]. 2023 [cited 2024 Sep 27]. Available from: https://clinicaltrials.gov/study/NCT04485104

23. Cock H, Wu J, Devinsky O, Joshi C, Miller I, Roberts C, et al. Time to onset of cannabidiol (CBD) treatment effect and resolution of adverse events in the tuberous sclerosis complex Phase 3 randomised controlled trial (GWPCARE6). Dev Med Child Neurol. 2021;63(S1):77.

24. Cockerell I, Christensen J, Høi-Hansen C, Holst L, Frederiksen M, Lund K, et al. Efficacy and safety of everolimus in patients with tuberous sclerosis complex. Epilepsia. 2021;62(S3):158–9.

25. Coevoets R, Arican S, Hoogeveen-Westerveld M, Simons E, van den Ouweland A, Halley D, et al. A reliable cell-based assay for testing unclassified TSC2 gene variants. Eur J Hum Genet. 2009;17:301–10.

26. Combes FP, Baneyx G, Coello N, Zhu P, Sallas W, Yin H, et al. Population pharmacokinetics-pharmacodynamics of oral everolimus in patients with seizures associated with tuberous sclerosis complex. J Pharmacokinet Pharmacodyn. 2018;45:707–19.

27. Curatolo P, Franz DN, Lawson JA, Yapici Z, Ikeda H, Polster T, et al. Adjunctive everolimus for children and adolescents with treatment-refractory seizures associated with tuberous sclerosis complex: post-hoc analysis of the phase 3 EXIST-3 trial. Lancet Child Adolesc Health. 2018;2:495–504.

28. D’Onofrio G, Kuchenbuch M, Chemaly N, Hachon Le Camus C, Napuri S, Ville D, et al. Slow titration of cannabidiol add-on treatment in patients with drug resistant epilepsy provides a better safety profile. Eur J Neurol. 2020;27(S1):151.

29. De Ridder J, Lavanga M, Verhelle B, Vervisch J, Lemmens K, Kotulska K, et al. Prediction of neurodevelopment in infants with tuberous sclerosis complex using early EEG characteristics. Front Neurol. 2020;11:582891.

30. De Ridder J, Verhelle B, Vervisch J, Lemmens K, Kotulska K, Moavero R, et al. Early epileptiform EEG activity in infants with tuberous sclerosis complex predicts epilepsy and neurodevelopmental outcomes. Epilepsia. 2021;62:1208–19.

31. de Vries PJ, Belousova E, Benedik MP, Carter T, Cottin V, Curatolo P, et al. Natural clusters of tuberous sclerosis complex (TSC)-associated neuropsychiatric disorders (TAND): new findings from the TOSCA TAND research project. J Neurodev Disord. 2020;12:24.

32. Dorofeeva MI, Ermakov AI, Belousova ED. [The efficacy of topiramate (topamax) in the treatment of resistant epilepsy in children]. Zh Nevrol Psikhiatr Im S S Korsakova. 2005;105:21–3.

33. Ebrahimi-Fakhari D, Agricola KD, Tudor C, Krueger D, Franz DN. Cannabidiol elevates mechanistic target of rapamycin inhibitor levels in patients with tuberous sclerosis complex. Pediatr Neurol. 2020;105:59–61.

34. Farges D, Sigg N, Ville D, Martin L. Use of mTOR inhibitors (rapalogs) for the treatment of skin changes in tuberous sclerosis complex. Arch Pediatr. 2022;29:5S20–4.

35. Fleury P, de Groot WP, Delleman JW, Verbeeten B, Frankenmolen-Witkiezwicz IM. Tuberous sclerosis: the incidence of sporadic cases versus familial cases. Brain Dev. 1980;2:107–17.

36. Franco V, Bialer M, Perucca E. Cannabidiol in the treatment of epilepsy: current evidence and perspectives for further research. Neuropharmacology. 2021;185:108442.

37. Franz DN, Agricola K, Mays M, Tudor C, Care MM, Holland-Bouley K, et al. Everolimus for subependymal giant cell astrocytoma: 5-year final analysis. Ann Neurol. 2015;78:929–38.

38. Franz DN, Lawson JA, Yapici Z, Ikeda H, Polster T, Nabbout R, et al. Sustained seizure reduction with adjunctive everolimus for treatment-refractory seizures associated with tuberous sclerosis complex (TSC): long-term results from the phase 3 EXIST-3 study (S207). Ann Neurol. 2017;82(Supplement 21):S66.

39. García-Martín D, Martínez-Córdoba N, Andrea-Cubides P, Espinosa-García ET, Araujo-Polaina AF. Tuberous sclerosis: clinical and imaging findings in a series of Colombian pediatric patients. Rev Mex Pediatr. 2021;88:10–7.

40. Georgieva D, Langley J, Hartkopf K, Hawk L, Margolis A, Struck A, et al. Real-world, long-term evaluation of the tolerability and therapy retention of Epidiolex^®^ (cannabidiol) in patients with refractory epilepsy. Epilepsy Behav. 2023;141:109159.

41. Giacaman A, Corral-Magaña O, Salinas J, Escudero-Góngora M, Boix-Vilanova J, Rosell J, et al. Clinical and genetic findings in 27 patients with tuberous sclerosis complex. Pediatr Dermatol. 2017;34(S2):S14.

42. Golub V, Reddy DS. Cannabidiol therapy for refractory epilepsy and seizure disorders. Adv Exp Med Biol. 2021;1264:93–110.

43. Gosnell ES, Krueger D, Ruck P, Buff-Lindner AH, Horn PS, Griffith M. Oral manifestations and quality of life in children with tuberous sclerosis complex: a descriptive study. Pediatr Dent. 2021;43:140–4.

44. Grigorieva A, Dorofeeva M, Gorchkhanova Z, Belousova E. Preventive epilepsy therapy in patients with tuberous sclerosis complex (TSC). Epilepsia. 2018;59(S3):S291.

45. Gupta A, de Bruyn G, Tousseyn S, Krishnan B, Lagae L, Agarwal N, et al. Epilepsy and neurodevelopmental comorbidities in tuberous sclerosis complex: a natural history study. Pediatr Neurol. 2020;106:10–6.

46. Hallett L, Foster T, Valentim J, Blieden M, Liu Z. PND19 Humanistic and economic burden in tuberous sclerosis complex with neurological manifestations: systematic review. Value Health. 2011;14:A205.

47. Hamer HM, Pfäfflin M, Baier H, Bösebeck F, Franz M, Holtkamp M, et al. Characteristics and healthcare situation of adult patients with tuberous sclerosis complex in German epilepsy centers. Epilepsy Behav. 2018;82:64–7.

48. Hertzberg C, Wilken B, Nathrath M, Gnekow A, Penzien J, Janßen G, et al. An opel-label, multi-center, expanded access study of RAD001 (everolimus) in patients with TSC SEGA. Neuropediatrics. 2012;43:FV12_03.

49. Hirata Y, Hamano S, Ikemoto S, Kikuchi K, Koichihara 小一原, Matsuura T, et al. Effects of vigabatrin on patients with epileptic spasms and focal seizures. Journal of the Japan Epilepsy Society. 2020;38:139–46.

50. Hulshof HM, Benova B, Krsek P, Kyncl M, Lequin MH, Belohlavkova A, et al. The epileptogenic zone in children with tuberous sclerosis complex is characterized by prominent features of focal cortical dysplasia. Epilepsia Open. 2021;6:663–71.

51. Jeong A, Wong M. Systemic disease manifestations associated with epilepsy in tuberous sclerosis complex. Epilepsia. 2016;57:1443–9.

52. Joshi C. What are the chances that epilepsy surgery will allow my child to come off meds? Assessing pediatric epilepsy surgery outcomes: data on ASM freedom. Epilepsy Curr. 2022;22:339–41.

53. Jóźwiak S. EPISTOP project - data from current status. 2017 International Research Conference on TSC and LAM [Internet]. 2017. Available from: https://online.fliphtml5.com/tosk/caws/

54. Jozwiak S, Aronica E, Curatolo P, Jansen A, Jansen F, Kotulska K, et al. Prevention of epilepsy and its comorbidities in tuberous sclerosis complex. Epilepsia. 2019;60(S2):12 (Abstract P014).

55. Jozwiak S, Goodman M, Lamm SH. Poor mental development in patients with tuberous sclerosis complex: clinical risk factors. Arch Neurol. 1998;55:379–84.

56. Jóźwiak S, Kasprzyk-Obara J, Domańska-Pakieła D. Phacomatoses: structural substare of epilepsy. Neurol Neurochir Pol. 2000;34(Suppl 1):243–51.

57. Kafle DR, Oli KK. Clinical profile of patients with recurrent seizure in tertiary care hospital in Nepal. Kathmandu Univ Med J (KUMJ). 2014;12:202–6.

58. Kingswood C, Dew R, Gray E. Management of tuberous sclerosis complex in England: insights into real-world clinical practice. Value Health. 2015;18(3):A249 (Abstract PHS13).

59. Kotulska K, Kwiatkowski DJ, Curatolo P, Weschke B, Riney K, Jansen F, et al. Prevention of epilepsy in infants with tuberous sclerosis complex in the EPISTOP trial. Ann Neurol. 2021;89:304–14.

60. Kwan P, Thiele E, Bebin E, Filloux F, Jansen F, Loftus R, et al. Long-term safety and efficacy of add-on cannabidiol for treatment of seizures associated with tuberous sclerosis complex in an open-label extension. Epilepsia. 2021;62:145–6.

61. La Briola F, Vignoli A, Turner K, Chiesa V, Zambrelli E, Piazzini A, et al. Epilessia nella sclerosi tuberosa : analisi di una casistica = Epilepsy in Tuberous Sclerosis : analysis of case studies. Bollettino – Lega Italiana contro l’Epilessia. 2013;145:17–9.

62. Landazuri P, Shih J, Leuthardt E, Ben-Haim S, Neimat J, Tovar-Spinoza Z, et al. A prospective multicenter study of laser ablation for drug resistant epilepsy - One year outcomes. Epilepsy Res. 2020;167:106473.

63. Lo SH, Marshall J, Skrobanski H, Lloyd A. Patient and caregiver health state utilities in tuberous sclerosis complex. Pharmacoecon Open. 2022;6:105–21.

64. Lukas V, Neal L, Mccain S, Silvia M, Boggs J, Stem T, et al. MP61-10 Catastrophic events related to tuberous sclerosis complex are unlikely in those undergoing routine surveillance. Journal of Urology. 2021;206(Suppl 3):e1086–7.

65. Manea I, Lupescu I-C, Lupescu IG, Solomon EA, Dulamea A. Neurological and clinical findings in patients with tuberous sclerosis before and after treatment with everolimus. Eur J Neurol. 2017;24(Suppl. 1):436 (Abstract EP3164).

66. Moavero R, Voci A, Romigi A, Bisulli F, Luisi C, Vigevano F, et al. Sleep disorders in adults with tuberous sclerosis complex: a questionnaire-based study. Sleep Med. 2022;100:S38–9.

67. Nabavi Nouri M, Zak M, Whitney R, Haile D. P.087 The landscape of paediatric tuberous sclerosis complex (TSC) neurological care in canada: results from a national survey. Can J Neurol Sci. 2022;49:S30–1.

68. Neal L, McCain S, Boggs J, Stem T, Miles M, Strowd R. Catastrophic events related to tuberous sclerosis complex (TSC) are unlikely in a heterogeneous adult cohort of TSC patients (2164). Neurology. 2021;96:2164.

69. Nickels K. Cannabidiol in patients with intractable epilepsy due to TSC: a possible medication but not a miracle. Epilepsy Curr. 2017;17:91–2.

70. Pack S, Moss A. Patterns in the noise: using chaos theory to compare the deterministic behavior of seizures caused by tuberous sclerosis complex to those of the wider epilepsy community using Seizure Tracker data. 2019 International TSC Research Conference [Internet]. 2019. Available from: https://online.fliphtml5.com/tosk/ghir/#p=72

71. Pfirmann P, Combe C, Rigothier C. Description of a multidisciplinary model of care in a French cohort of tuberous sclerosis complex adult patients: PO1522. J Am Soc Nephrol. 2020;31:487.

72. Raucci U, Spalice A, Basile L, Guardalà C, Nasta L, Terenzi S, et al. [New drugs in the treatment of childhood epilepsy: vigabatrin (study of 61 subjects)]. Pediatr Med Chir. 1994;16:575–8.

73. Riikonen R. Epidemiological data of West syndrome in Finland. Brain Dev. 2001;23:539–41.

74. Roach E, DiMario F, Kandt R, Northrup H. Tuberous Sclerosis Consensus Conference: recommendations for diagnostic evaluation. National Tuberous Sclerosis Association. J Child Neurol. 1999;14:401–7.

75. Rovira À, Ruiz-Falcó M, García-Esparza E, López-Laso E, Macaya A, Málaga I, et al. Recommendations for the radiological diagnosis and follow-up of neuropathological abnormalities associated with tuberous sclerosis complex. J Neurooncol. 2014;118:205–23.

76. Sahebkar F, Thiele E, Bebin E, Bhathal H, Jansen F, Kotulska K, et al. Cannabidiol (CBD) treatment in patients with seizures associated with tuberous sclerosis complex (TSC): a randomised, double-blind, placebo-controlled phase 3 trial (GWPCARE6). Dev Med Child Neurol. 2020;62:4–14.

77. Samueli S, Dressler A, Gröppel G, Scholl T, Feucht M. Everolimus in infants with tuberous sclerosis complex-related West syndrome: First results from a single-center prospective observational study. Epilepsia. 2018;59:e142–6.

78. Samueli S, Dressler A, Gröppel G, Kasprian G, Laccone F, Scholl T, et al. Experience with everolimus in infants and toddlers with TSC‐related epilepsies (Abstract P651). Epilepsia; 2018 [cited 2024 Sep 30]. p. S296. Available from: https://onlinelibrary.wiley.com/doi/epdf/10.1111/epi.14612

79. Sanchez-Carpintero R, Cock H, Wu JY, Devinsky O, Joshi C, Miller I, et al. Time to onset of cannabidiol treatment effect and resolution of adverse events in tuberous sclerosis complex randomised controlled trial (GWPCARE6). Epilepsia. 2021;62:3-364 (Abstract 141).

80. Sharma S, Kaushik J, Srivastava K, Goswami J, Sahu J, Vinayan K, et al. Association of Child Neurology (AOCN) - Indian Epilepsy Society (IES) Consensus Guidelines for the Diagnosis and Management of West Syndrome. Indian Pediatr. 2021;58:54–66.

81. Słowińska M, Golec W, Jóźwiak S. Prevention of epilepsy in humans - truth or myth? The experience from Sturge-Weber syndrome and tuberous sclerosis complex. Neurol Neurochir Pol. 2019;53:190–3.

82. Sparagana S, Belousova E, Jozwiak S, Korf B, Frost M, Kuperman R, et al. Everolimus treatment of subependymal giant cell astrocytomas (SEGAs) associated with tuberous sclerosis complex (TSC): the EXIST-1 trial. Neuro Oncol. 2011;13:iii41–68.

83. Sparagana S, Swallow E, Song J, King S, Peeples M, Signorovitch J, et al. Epilepsy treatment patterns among patients with tuberous sclerosis complex (P5.161). Neurology. 2016;86:P5.161.

84. Story T, Reaven N, Funk S. The direct cost burden of tuberous sclerosis complex in US commercial and Medicaid populations (Abstract 4). 2019 International TSC Research Conference [Internet]. 2019. Available from: https://online.fliphtml5.com/tosk/ghir/#p=72

85. Strzelczyk A, Schubert-Bast S, Zöllner JP, Simon A, Wyatt G, Holland R, et al. PND50 Epidemiology, healthcare resource use, and mortality in patients with tuberous sclerosis complex: a population-based study on German health insurance DATA. Value Health. 2020;23:S631–2.

86. Strzelczyk A, Schubert-Bast S, Zöllner JP, Simon A, Wyatt G, Holland R, et al. P 55. Epidemiology, healthcare resource use, and mortality in patients with tuberous sclerosis complex: a population-based study on German health insurance data. Clin Neurophysiol. 2021;132:e25–6.

87. Sugiyama I, Imai K, Yamaguchi Y, Ochi A, Akizuki Y, Go C, et al. Localization of epileptic foci in children with intractable epilepsy secondary to multiple cortical tubers by using synthetic aperture magnetometry kurtosis. J Neurosurg Pediatr. 2009;4:515–22.

88. Svarrer EM, Fischer CM, Frederiksen MG, Born AP, Hoei-Hansen CE. Everolimus as adjunctive treatment in tuberous sclerosis complex-associated epilepsy in children. Dan Med J. 2019;66:A5582.

89. Tohyama J. TuberOus SClerosis registry to increAse disease awareness(TOSCA):2nd interim analysis results. No To Hattatsu. 2016;48:S272.

90. Tuft M, Olsen T. Tuberous sclerosis complex (TSC): A Norwegian interdisciplinary guide. Epilepsia. 2015;56(S1):91 (Abstract P0349).

91. Tye C, McEwen FS, Liang H, Woodhouse E, Underwood L, Shephard E, et al. Epilepsy severity mediates association between mutation type and ADHD symptoms in tuberous sclerosis complex. Epilepsia. 2023;64:e30–5.

92. Wheless J, Bebin EM, Filloux F, Kwan P, Jansen FE, Loftus R, et al. Long-term safety and efficacy of add-on cannabidiol (CBD) for treatment of seizures associated with tuberous sclerosis complex (TSC) in an open-label extension (OLE) trial (GWPCARE6). Neurology. 2021;96(Suppl. 15):1127 (Abstract).

93. World Health Organization. EUCTR2010-019519-39-NL. Efficacy of RAD001/everolimus in Autism and NeuroPsychological deficits in children with tuberous sclerosis complex (RAPIT-trial) [Internet]. 2011 [cited 2024 Nov 6]. Available from: https://trialsearch.who.int/Trial2.aspx?TrialID=EUCTR2010-019519-39-NL

94. World Health Organization. ChiCTR2000031984. Sirolimus as adjuctive therapy in patients with tuberous sclerosis complex and epilepsy [Internet]. 2020 [cited 2024 Nov 6]. Available from: https://trialsearch.who.int/Trial2.aspx?TrialID=ChiCTR2000031984

95. World Health Organization. EUCTR2020-003231-19-PL. Randomized, placebo-controlled, double-blind and double-dummy clinical trial comparing the safety, tolerability, and efficacy of vigabatrin and rapamycin in a preventive treatment of infants with Tuberous Sclerosis Complex (ViRap) - ViRap. Updated. 26 April 2023 [Internet]. 2023 [cited 2024 Sep 27]. Available from: https://trialsearch.who.int/Trial2.aspx?TrialID=EUCTR2020-003231-19-PL

96. Yapici Z, Fan P-C, Lawson J, Belousova E, Gazri H, Ridolfi A, et al. Long‐term safety, rollover study of adjunctive everolimus in patients with tuberous sclerosis complex (TSC)‐associated treatment‐refractory seizures who continue to benefit from everolimus treatment after completion of EXIST‐3. Epilepsia. 2018;59(S3):S1–376.

97. Zak M, Chan V, McCoy B. Mind the gap: initiation of a comprehensive tuberous sclerosis complex care clinic in Toronto, Canada: a gap analysis to determine level of surveillance and care delivery prior to the initial TSC clinic visit. American Epilepsy Society Annual Meeting 2017 [Internet]. 2017 [cited 2024 Oct 1]. Available from: https://aesnet.org/abstractslisting/mind-the-gap--initiation-of-a-comprehensive-tuberous-sclerosis-complex-care-clinic-in-toronto--canada--a-gap-analysis-to-determine-level-of-surveillance-and-care-delivery-prior-to-the-initial-tsc-clinic-visit

98. Zöllner J, Grau J, Schubert-Bast S, Kurlemann G, Hertzberg C, Wiemer-Kruel A, et al. Direct and indirect costs and cost drivers of tuberous sclerosis complex in children, adolescents, and caregivers: a multicenter cohort study. Epilepsia. 2021;62:304–5.
