# Supplementary material for: Burden of illness in tuberous sclerosis complex-associated epilepsy: a systematic literature review of epidemiology, health-related quality of life, costs and resource use
Source: Orphanet J Rare Dis. 2025 Nov 25;20:609. doi: 10.1186/s13023-025-03975-y (PMC12648938; doi:10.1186/s13023-025-03975-y)
Supplement: Supplementary file 2 — Additional file2 [file 13023_2025_3975_MOESM2_ESM.docx]

# Additional online material for:

**Burden of illness in tuberous sclerosis complex-associated epilepsy: a systematic literature review of epidemiology, health-related quality of life, costs and resource use**

# Online additional table S1. Prevalence of TSC-associated epilepsy (n=63 studies)

| **Study** | **Location (country/ region)** | **Study patient population (number of patients / age / disease group)** | | **Date (year)^†^** | | **Prevalence of TSC-associated  epilepsy** | | |
| --- | --- | --- | --- | --- | --- | --- | --- | --- |
| ***Any age with TSC^‡^*** | | | | | | | |  |
| Fleury 1990 [1] | Netherlands | | 118 patients with TSC | | 1990 | | 74% had epilepsy | |
| Hunt 1993 [2] | UK | | 300 patients with TSC | | 1993 | | 93% had at least one seizure  63% had current seizures | |
| Webb 1996 [3] | UK | | 131 patients with TSC | | 1996 | | 78% had epilepsy | |
| Joinson 2003 [4] | UK | | 108 patients with TSC | | 1998 | | 79% had epilepsy | |
| Winterkorn 2007 [5] | USA | | 107 patients with TSC | | 2002–2006 | | 87.9% (across IQ/DQ ≥70 and IQ/DQ <70) | |
| Muzykewicz 2007 [6] | USA | | 241 patients with TSC | | 2007 | | 87% had epilepsy | |
| Chu-Shore 2010 [7] | USA | | 291 patients with TSC | | 2008 | | 85.2% had at least one seizure  54.1% developed multiple seizure types | |
| Numis 2011 [8] | USA | | 103 patients with TSC | | 2002–2009 | | TSC with ASD: 98% had epilepsy  TSC without ASD: 82% had epilepsy  88.3% (across patients both with and without ASD) | |
| Hallett 2011 [9] | International | | 119 publications in a SLR reporting on patients with TSC | | 2011 | | 66–93% had epilepsy | |
| Wataya-Kaneda 2013 [10] | Japan | | 166 patients with TSC | | 2011 | | 63% had epilepsy | |
| Pashos 2012 [11] | USA | | 380 patients with TSC | | 2012 | | 46% had epilepsy | |
| Sun 2012 [12] | USA | | 1,249 patients with TSC | | 2012 | | 16.1% had epilepsy | |
| van Eeghen 2012 [13] | USA | | 66 patients with TSC | | 2012 | | 88% had epilepsy | |
| Vignoli 2013 [14] | Italy | | 160 patients with TSC | | 2013 | | 72.5% had epilepsy  4.4% had one isolated seizure | |
| Wilbur 2017 [15] | Canada | | 81 patients with TSC | | 2014 | | 91% had epilepsy (of whom 39% had refractory epilepsy) | |
| Crawford 2015 [16] | UK | | 341 patients with TSC | | 2015 | | 71% had epilepsy | |
| Vignoli 2015 [17] | Italy | | 42 patients with TSC | | 2015 | | 85.7% had epilepsy | |
| Wilson 2016 [18] | USA | | 5,655 patients with TSC | | 2016 | | 41.2% had epilepsy | |
| Strzelczyk 2021b [19] | Germany | | 256 patients with TSC | | 2016 | | 36% had epilepsy | |
| Chung 2017b [20] | Australia | | 70 patients with TSC | | 2017 | | 87% developed seizures | |
| Jeong 2017 [21] | USA | | 1,965 patients with TSC | | 2017 | | 86.4% had epilepsy | |
| Orazem Mrak 2017 [22] | Slovenia | | 18 patients with TSC | | 2017 | | 83% had epilepsy | |
| Overwater 2017 [23] | Netherlands | | 102 patients with TSC | | 2017 | | 86% had epilepsy | |
| Welin 2017 [24] | Sweden | | 551 patients with TSC | | 2017 | | 70.1% had epilepsy | |
| Song 2018 [25] | USA, Belgium | | 1,328 patients with TSC | | 2018 | | 83.6% had epilepsy | |
| Farach 2020 [26] | USA | | 333 patients with TSC | | 2013–2019 | | 78.4% had epilepsy | |
| Nabbout 2019 [27] | International | | 2,216 patients with TSC | | 2019 | | 83.6% had epilepsy | |
| Erdal 2020 [28] | Istanbul | | 113 patients with TSC | | 2020 | | 85.8% had epilepsy 75% had onset epilepsy in first year of life | |
| Grau 2021 [29] | Germany | | 184 patients with TSC | | 2021 | | 91.8% had epilepsy  47.8% had recurrent seizures | |
| Nabbout 2021 [30] | International | | 1,879 patients with TSC and epilepsy | | 2021 | | Patients diagnosed with infantile spasms or focal seizures over time:  1940–1950: 45.0%  >1950–1960: 44.3%  >1960–1970: 66.3%  >1970–1980: 67.8%  >1980–1990: 79.7%  >1990–1995: 88.4%  >1995–2000: 88.4%  >2000–2005: 91.0%  >2005–2010: 93.3%  >2010–2015: 91.8% | |
| Peng 2021 [31] | Taiwan | | 471 patients with TSC | | 2021 | | 76.6% had epilepsy | |
| ***Children with TSC*** | | | | | | | |  |
| Rok 2005 [32] | Poland | | 4 children (aged <18 years) with a *TSC2* mutation | | 2005 | | 100% had epilepsy | |
| de Vries 2007 [33] | UK | | 258 children (aged <18 years) with TSC | | 2007 | | 92.2% had epilepsy | |
| Hong 2016 [34] | Taiwan | | 237 children (aged ≤18 years) with TSC | | 2010 | | 81% had epilepsy | |
| Demuth 2013 [35] | UK | | 244 patients with TSC^§^ | | 2012 | | 72% had epilepsy by age 4 years | |
| O’Connor 2012 [36] | Australia | | 31 children with TSC^§^ | | 2012 | | 93% had epilepsy | |
| Graffigna 2013 [37] | Italy | | 48 children (mean age 12 years) with TSC and SEGA | | 2013 | | 81.2% had epilepsy | |
| Amin 2019 [38] | UK | | 35 children with TSC^§^ | | 2014 | | 82.9% had epilepsy | |
| Kotulska 2014 [39] | Poland | | 421 children with TSC^§^ | | 2014 | | 5.7% had epilepsy as neonates  86.9% had epilepsy after a mean of 45 months of age | |
| Gumbelevičiene 2014 [40] | Lithuania | | 44 children (aged 2 weeks to 16 years) with TSC | | 2014 | | 93% had epilepsy | |
| Rentz 2015a [41] | USA | | 179 children (aged ≤18 years) with TSC | | 2015 | | 76.5% had epilepsy | |
| Wheless 2016 [42] | USA | | 2,163 patients with TSC | | 2016 | | 38% of children aged 0–4 had epilepsy after diagnosis of TSC | |
| Vorgia 2016 [43] | Greece | | Children with TSC^§^ | | 2016 | | 100% had epilepsy | |
| da Câmara 2016 [44] | Portugal | | 21 children with TSC^§^ | | 2016 | | 90% had epilepsy | |
| Kingswood 2016 [45] | UK | | 94 children (aged <18 years) with TSC | | 2016 | | 76.6% children had epilepsy | |
| Giacaman 2017 [46] | Spain | | 31 children with TSC^§^ | | 2017 | | 39% required ≥1 ASM  (35% presented with epilepsy as the first symptom of TSC) | |
| Capal 2017 [47] | USA | | 130 children (aged <36 months; mean age 5.2 months) with TSC | | 2017 | | 73.1% had epilepsy after 24 months | |
| Davis 2017 [48] | USA | | 130 children (aged ≤36 months) with TSC | | 2017 | | 76% had epilepsy  73% developed epilepsy within the first year of life  41% had 2 seizure types; 3% had 3 seizure types | |
| Jansen 2017 [49] | International | | 66 children with TSC^§^ | | 2017 | | 40.9% had epilepsy | |
| Patel 2017 [50] | International | | 49 studies from an SLR reporting on children with TSC^§^ | | 2017 | | 71.4% children had epilepsy | |
| Shepherd 2017 [51] | UK | | 104 children (aged <18 years) with TSC | | 2017 | | 77.8% had epilepsy | |
| de Groen 2019 [52] | USA | | 208 children with TSC^§^ | | 2019 | | 80.8% had epilepsy | |
| Schoenberger 2019 [53] | USA | | 137 children (aged 3–36 months) with TSC | | 2019 | | 78.1% had epilepsy by 36 months old | |
| Wu 2019 [54] | USA | | 32 infants (aged ≤7 months) with TSC | | 2019 | | 62.5% developed epilepsy over 24 months | |
| Hulshof 2020 [55] | Netherlands | | 34 children with TSC^§^ | | 2020 | | 85.3% had epilepsy | |
| Ding 2021 [56] | China | | 124 children (aged ≤18 years) with TSC | | 2021 | | 84.7% had epilepsy | |
| Ihnen 2021 [57] | USA | | 156 children (aged ≤36 months) with TSC | | 2021 | | 79% had epilepsy | |
| ***Adults with TSC*** | | | | | | | |  |
| Hong 2016 [34] | Taiwan | | 234 adults (aged >18 years) | | 2010 | | 72.2% had epilepsy | |
| Staley 2011 [58] | USA | | 30 adults (aged ≥21 years) with TSC | | 2011 | | 3% adults with no previous seizures had new onset of seizures in adulthood  13% adults had a history of seizures | |
| Tierney 2011 [59] | UK | | 21 adults (aged ≥18 years) with TSC and IQ >80 | | 2011 | | 52.4% adults had epilepsy  Additional 14.3% had seizures only in childhood | |
| Amin 2019 [38] | UK | | 56 adults (median age 34 years) with TSC | | 2014 | | 76.8% had epilepsy | |
| Rentz 2015a [41] | USA | | 497 adults (aged ≥19 years) with TSC | | 2015 | | 23.7% had epilepsy | |
| Vergeer 2019 [60] | Netherlands | | 363 adults with TSC^§^ | | 2015 | | 77.1% had epilepsy | |
| Kingswood 2016 [45] | UK | | 240 adults (aged ≥18 years) with TSC | | 2016 | | 66.3% had epilepsy | |
| Jansen 2017 [49] | International | | 45 adults with TSC from 6 European countries^§^ | | 2017 | | 60% had epilepsy | |
| Patel 2017 [50] | International | | 49 studies from an SLR reporting on adults with TSC^§^ | | 2017 | | 32% had epilepsy | |
| Shepherd 2017 [51] | UK | | 182 adults (aged ≥18 years) with TSC | | 2017 | | ~70% had epilepsy | |
| Li 2019 [61] | China | | 312 adults with TSC^§^ | | 2019 | | 78% had epilepsy | |
| Vignoli 2021 [62] | Italy | | 257 adults (aged >18 years) with TSC | | 2021 | | 71.2% had epilepsy  1.6% had a single seizure | |
| Zöllner 2021b [63] | Germany | | 192 adults (aged ≥18 years) with TSC | | 2021 | | 72.9% had epilepsy  39.1% had recurrent seizures | |

^†^Dates are displayed as the last year of data collection. Where not specified, this is the year of publication. ^‡^Mixed-age population including children and adult populations. ^§^Population age not defined

ASD: autism spectrum disorder; ASM: antiseizure medication; DQ: developmental quotient; IQ: intelligence quotient; NIS: National Inpatient Sample; SEGA: subependymal giant cell astrocytoma; SLR: systematic literature review; TSC: tuberous sclerosis complex

# Online additional table S2. PRO tool outcomes and associated QOL for mixed-age populations with TSC with (n=11) or with/without (n=3) epilepsy (n=14 studies)

| **Study and patient population** | **PRO tool** | **PRO scores** | **Determinants of poorer scores** |
| --- | --- | --- | --- |
| Bar 2019 [64]   - Adults (aged 18–55 years) with TSC and epilepsy | Adapted epilepsy survey for patients with DS | QOL mediocre = 34%  QOL bad = 18%  QOL fair = 38%  QOL good = 7%  QOL excellent = 3% | - NA |
| Liang 2010 [65]   - Children and adults  (aged 6–23 years) with TSC and epilepsy | WIS-CR | *Mean scores at baseline; change at 2 years post-surgery:*  FSIQ = 57.83–77.46; 3.45–9.00  VIQ = 61.75–79.15; 2.60–6.53  PIQ = 54.75–77.00; 2.69–13.75  *Change at 2 years, medicine/surgery:* FSIQ; VIQ; PIQ = −3.25/5.60; −4.13/4.96; −3.38/6.80 | *Baseline scores:*   - CCT and resection vs resection - Low vs normal IQ   *Change at 2 years post-surgery:*   - Seizures vs seizure-free - Resection vs CCT and resection (FSIQ*; PIQ**) - Normal vs low IQ (PIQ*)   *Medicine vs surgery*   - Medicine vs surgery** |
| Liang 2017 [66]   - Children and adults  (aged 5–28 years) with TSC‑associated seizures | WIS-CR | *Mean scores at baseline; change at 5 years post-surgery:*  FSIQ = 57.85–76.71; 2.90–10.07  VIQ = 59.47–77.94; 2.05–6.90  PIQ = 57.00–77.00; 3.95–13.57  *Mean scores at baseline; change at 5 years, medicine/surgery:*  FSIQ = 62.93; –6.33 / 64.14; 7.12  VIQ = 64.87; –6.67 / 65.63; 4.90  PIQ = 62.80; –6.53 / 63.80; 9.61 | *Baseline scores:*   - CCT and resection vs resection - IQ <70 vs ≥70 - IS vs no IS - Medicine vs surgery   *Change at 5 years post-surgery:*   - Seizures vs seizure-free** - Age at surgery >10 vs ≤10 years   *Medicine vs surgery at 5 years*   - Medicine vs surgery** |
| Pearsson 2022 [67]   - Patients with TSC who had epilepsy surgery^†^ | Telephone interview | *Surgery rating, caregivers (n):*  Beneficial = 11  Neutral = 2  Harmful = 0 | - NA |
| Zaroff 2005 [68]   - Patients (aged 2–21 years) with TSC and refractory epilepsy who had epilepsy surgery | WISC-III | Pre-op/post-op IQ score = 87/84 | - NA |
| Liang 2010 [65]   - Children and adults  (aged 6–23 years) with TSC and epilepsy | QOLIE-31 | *Mean scores at baseline; change at 2 years post-surgery:*  Overall QOL = 40.85–47.77; 2.20–13.63  *Change at 2 years, medicine/surgery:*  Mean overall QOL = −4.13/4.96 | *Baseline scores:*   - CCT and resection vs resection - Low vs normal IQ   *Change at 2 years post-surgery:*   - Non-seizure-free vs seizure-free* - Resection vs CCT and resection** - Normal IQ vs low IQ   *Medicine vs surgery*   - Medicine vs surgery** |
| Liang 2017 [66]   - Children and adults  (aged 5–28 years) with TSC‑associated seizures | QOLIE-31 | *Mean score at baseline; change at 5 years post-surgery:*  Overall QOL = 43.18–52.59; 4.57–12.80  *Mean scores at baseline; change at 5 years:*  Medicine = 43.47; –6.93  Surgery = 47.76; 9.41 | *Baseline scores:*   - Medicine vs surgery - CCT and resection vs resection - IQ <70 vs ≥70 - IS vs no IS - Seizures for >10 vs ≤10 years   *Change at 5 years post-surgery:*   - Seizures vs no seizures**   *Medicine vs surgery at 5 years*   - Medicine vs surgery** |
| Liu 2020 [69]   - Children and adults  (aged 0.5–47 years) with TSC who had epilepsy surgery | QOLIE-31/QOLCE | *Mean total score; change at 1 year:*  All patients: 53.99; 8.32  Subgroups: 49.26–62.09; 1.57–10.88 | *Baseline scores:*   - Age at onset ≤12 vs >12 years** - IQ <70 vs ≥70** - CCT and resection vs resection** - Generalised spasm vs seizures   *Change at 1 year post-surgery:*   - Postoperative seizures vs seizure-free** - >12 vs ≤12 months at seizure onset* - >10 vs ≤10 years of seizures - IQ ≥70 vs <70** - Generalised seizures vs focal seizures and generalised spasms |
| de Vries 2018 [70]   - Adults (aged ≥18 years) with TSC and refractory partial-onset seizures | QOLIE-31-P | Mean (SD) total score change at week 12: non-responders = −0.6 (15.3); responders = 15.2 (15.7) | - Non-responders vs responders to EVE treatment* |
| Zöllner 2021a [71]   - Adults (aged 18–61 years) with TSC (77% had epilepsy) | QOLIE-31 VAS | Mean (SD) overall score: seizures = 56.7 (19.4); no seizures = 68.8 (20.5) | - Active epilepsy vs seizure freedom** |
| Moavero 2022 [72]   - Adults (aged 18.7–71.7 years) with TSC (82% had history of epilepsy) | PSQI, ISI, ESS | *PSQI:*  Mean (SD) sleep quality score = 6.1 (4.5)  Median score: 6, 3  *ISI:*  Mean (SD) score = 7.1 (6.2)  Median score: 7, 3  *ESS:*  Mean (SD) total score = 5.2 (4.9)  Median score: 4, 3.5 | *PSQI:*  *Median score:*   - TAND vs no TAND**   *Proportion of patients with score >5:*   - Epilepsy vs no epilepsy* - Seizures vs seizure-free** - TAND vs no TAND   *Risk factors of having score >5:*   - Active seizures** - Active epilepsy* - TAND comorbidity*   *ISI:*  *Median score:*   - TAND vs no TAND**   *Proportion of patients with score ≥8:*   - Epilepsy vs no epilepsy - Seizures vs seizure-free*** - TAND vs no TAND*   *Risk factors of having score ≥8:*   - Active seizures** - Active epilepsy** - TAND comorbidity*   *ESS:*  *Median score:*   - TAND vs no TAND   *Proportion of patients with score >10:*   - Epilepsy vs no epilepsy - Seizures vs seizure-free - TAND vs no TAND |
| Tierney 2011 [59]   - Adults (aged 22–56 years) with TSC and IQ >80 (67% had epilepsy) and healthy controls (aged 17–55 years) | ADSA | *Mean (SD) total T score:*  Patients = 53.61 (11.31)  Healthy controls: 43.11 (9.83) | - TSC vs no TSC** |
| Zamponi 2010 [73]   - Children and adults  (aged 2–35 years) with TSC and multifocal or diffuse seizures | VAS 0–100, VABS | Mean QOL VAS score: baseline; change at 1 year = 51; 67  *Change at 1 year in VABS, %, P-value vs baseline:*  Total score = 12**  Communication and socialisation = 12*  Daily life abilities = 5* | - VAS: Baseline vs 1 year of VNS treatment** - VABS: Baseline vs 1 year of VNS treatment |
| Zaroff 2005 [68]   - Patients (aged 2–21 years) with TSC and refractory epilepsy who had epilepsy surgery | VABS | Mean DQ/IQ (SD), pre-surgery; post-surgery = 55 (20.3); 49 (16.6)  *Score range (pre-surgery; post-surgery):*  Communication = 25–74; 23–61  Daily living skills = 40–84; 34–58  Socialisation = 19–85; 41–63  Motor skills = 39–83; 30–53  Standard score = 25–73; 35–51 | - Post-surgery vs pre-surgery (small overall difference) |
| Kadish 2020 [74]   - Children and adults  (aged 1–31 years) with TSC and refractory epilepsy | VABS | *Median scores (baseline; post-EVE treatment):*  Communication = 74; 65  Mean DQ = 69.7; 38.7*  Communication DQ = 59.4; 40.3*  Social skills = 41; 26.3*  Motor skills = 54.7; 22.9*  DA = 29; 37 | - Post-EVE treatment vs baseline (non-DA scores) - Baseline vs post-EVE treatment (DA scores) |
| Vignoli 2015 [17]   - Children and adults  (aged 4–44 years) with TSC and epilepsy | SCQ | Mean scores: 4.2–16.4 | - Epilepsy vs no epilepsy* - Seizure onset <12 vs ≥12 months* - IS vs no IS* - IQ <70 vs ≥70* - Sleep disorders vs none* - TAND vs no TAND* |
| Kadish 2020 [74]   - Children and adults  (aged 1–31 years) with TSC and refractory epilepsy | BSID | *Median scores (baseline; post-EVE treatment):*  Mean DQ = 15.1; 11.0*  Expressive language = 9; 6.7  Fine motor skills = 14.3; 12.4*  Gross motor skills = 25; 17.1*  Mean DA = 6.0; 9.5*  Cognition DA = 4.3; 10*  Fine motor DA = 8; 9  Gross motor DA = 12; 18 | - Post-EVE treatment vs baseline (non-DA scores) - Baseline vs post-EVE treatment (DA scores) |
| Thiele 2021 [75]   - Children and adults (aged 1–57 years) with TSC and refractory epilepsy | PGIC | *Change from baseline, % of patients – placebo; CBD 25 mg/kg/day; CBD 50 mg/kg/day:*  Very much improved = 1; 8; 7  Much improved = 12; 26; 25  Slightly improved = 22; 25; 29  No change = 59; 33; 30  Slightly worse = 5; 4; 6  Much worse = 0; 3; 3  Very much worse = 0; 0; 0 | - Placebo vs CBD 25 mg/kg/day: odds ratio CBD vs placebo = 2.47 (95% CI 1.35, 4.52) - Placebo vs CBD 50 mg/kg/day: odds ratio CBD vs placebo = 2.33 (95% CI 1.27, 4.27) |

*P<0.05; **P<0.01; ***P<0.001. ^†^Population age not defined

ADSA: Attention-Deficit Scales for Adults; BSID: Bayley Scales of Infant Development; CBD: cannabidiol; CCT: corpus callosotomy; CI: confidence interval; DA: development age; DQ: developmental quotient; DS: Dravet syndrome; ESS: Epworth Sleepiness Scale; EVE: everolimus; FSIQ: full-scale intelligence quotient; IQ: intelligence quotient; IS, infantile spasm; ISI: Insomnia Severity Index; NA: not applicable; PGIC: Patient Global Impression of Change; PIQ: performance intelligence quotient; PRO: patient-reported outcome; PSQI: Pittsburgh Sleep Quality Index; QOL: quality of life; QOLCE: Quality of Life for Children with Epilepsy; QOLIE-31: 31-item Quality of Life in Epilepsy Inventory; QOLIE-31-P: 31-item Quality of Life in Epilepsy Inventory–Problems; SCQ: Social Communication Questionnaire; SD: standard deviation; TAND: tuberous sclerosis complex-associated neuropsychiatric disorders; TSC: tuberous sclerosis complex; VABS: Vineland Adaptive Behaviour Scale; VAS: visual analogue scale; VIQ: verbal intelligence quotient; VNS: vagus nerve stimulation; WISC-III: Wechsler Intelligence Scale for Children - Third Edition; WIS-CR: Wechsler Intelligence Scale (Chinese Revision)

# Online additional table S3. Direct costs and resource use in TSC with (n=12), with/without (n=17) and without (n=3) epilepsy and comparator populations (n=32 studies)

| **Study** | **Country** | **Currency and year** | **Study patient population – subgroup population** | **N** | **Direct costs or resource use** |
| --- | --- | --- | --- | --- | --- |
| Bauer 2016 [76] | USA | NA | Adults with TSC (mean age at last follow-up 35 years) – seizure-free | 10 | Mean number of drugs taken at last follow-up = 2.1 Mean number of drugs taken throughout follow-up (146.8 months) = 4.5 |
|  |  |  | Adults with TSC (mean age at last follow-up 35 years) – refractory epilepsy | 6 | Mean number of drugs taken at last follow-up = 3.8 Mean number of drugs taken throughout follow-up (147.5 months) = 11.7 |
| Betts 2020 [77] | USA | USD, 2017–2019 | Children and adults with TSC (mean age at index date 25.3 years) – TSC-associated epilepsy | 2,028 | *Annual incidence of all-cause HRU, mean (SD), IRR vs non-TSC-associated epilepsy:*  Outpatient visits = 5.64 (9.62), IRR = 1.03 (95% CI 0.94, 1.12; P=0.528)  Inpatient visits = 0.47 (1.31), IRR 0.72 (95% CI 0.63, 0.82; P<0.001)  ER visits = 0.95 (2.64), IRR 0.58 (95% CI 0.51, 0.66; P<0.001)  Clinic visits = 2.07 (15.57), IRR 1.16 (95% CI 0.82, 1.63; P=0.409)  Office visits = 6.82 (14.19), IRR 1.04 (95% CI 0.94, 1.14; P=0.452)  Other/unknown visits = 13.64 (45.99), IRR 1.16 (95% CI 0.99, 1.36; P=0.072)  *Annual incidence of epilepsy-related HRU, mean (SD), IRR vs non-TSC-associated epilepsy:*  Outpatient visits = 3.09 (6.22), IRR 1.96 (95% CI 1.77, 2.16; P<0.001)  Inpatient visits = 0.33 (1.00), IRR 1.27 (95% CI 1.10, 1.47; P=0.001)  ER visits = 0.46 (1.18), IRR 0.80 (95% CI 0.70, 0.90; P<0.001)  Clinic visits = 0.10 (0.67), IRR 1.12 (95% CI 0.76, 1.65; P=0.570)  Office visits = 2.67 (4.50), IRR 1.76 (95% CI 1.61, 1.92; P<0.001)  Other/unknown visits = 6.49 (29.84), IRR 1.70 (95% CI 1.34, 2.15; P<0.001)  *Annual all-cause cost/charges, mean (SD), cost difference vs non-TSC-associated epilepsy:*  Prescription drug costs = $18,836 (51,555), cost difference = $14,179 (95% CI 11,805, 16,531; P<0.001)  All medical services = $56,033 (524,254), cost difference = $4,293 (95% CI −12,862, 30,751, P=0.707)  Outpatient visits = $13,455 (38,274), cost difference = $1,897 (95% CI 188, 3949; P=0.031)  Inpatient visits = $2,106 (8,923), cost difference = $−637 (95% CI −1242, −50; P=0.047)  ER visits = $1,535 (4,907), cost difference = $−1,260 (95% CI −1,527, −1,008; P<0.001)  Clinic visits = $11,124 (110,854), cost difference = $4,499 (95% CI −49, 9,748; P=0.033)  Office visits = $3,805 (23,132), cost difference = $−1,476 (95% CI −3072, 187; P=0.063)  Other/unknown visits = $24,008 (504,841), cost difference = $1,270 (95% CI −13,113, 26,383; P=0.909)  *Annual epilepsy-related costs/charges, mean (SD), cost difference vs non-TSC-associated epilepsy:*  All epilepsy-related prescriptions = $17,017 (51,250), cost difference = $14,639 (95% CI 12,355, 16,944; P<0.001)  ASMs = $12,866 (42,211), cost difference = $10,543 (95% CI 8,670, 12,479; P<0.001)  Rescue medications = $123 (599), cost difference = $69 (95% CI 43, 98; P<0.001)  EVE = $4,028 (29,193), cost difference = $4,028 (95% CI 2,918, 5,358; P<0.001)  All epilepsy-related medical services = $28,455 (479,910), cost difference = $16,838 (95% CI 4,375, 39,611; P=0.019)  Outpatient visits = $8,532 (21,492), cost difference = $4,572 (95% CI 3,619, 5,608; P<0.001)  Inpatient visits = $1,282 (6,562), cost difference = $572 (95% CI 270, 886; P<0.001)  ER visits = $741 (2,364), cost difference = $−217 (95% CI −336, −102; P<0.001)  Clinic visits = $63 (1,062), cost difference = $26 (95% CI −17, 83; P=0.232)  Office visits = $1,433 (5,570), cost difference = $559 (95% CI 313, 823; P<0.001)  Other/unknown visits = $16,393 (478,180), cost difference = $11,327 (95% CI −952, 33,591; P=0.079) |
|  |  |  | Children and adults with TSC (mean age at index date 25.3 years) – non-TSC-associated epilepsy | 10,140 | *Mean (SD) annual incidence of all-cause HRU:*  Outpatient visits = 5.49 (10.68)  Inpatient visits = 0.65 (1.95)  ER visits = 1.62 (3.58)  Clinic visits = 1.79 (13.45)  Office visits = 6.57 (11.99)  Other/unknown visits = 11.76 (41.25)  *Mean (SD) annual incidence of epilepsy-related HRU:*  Outpatient visits = 1.58 (3.51)  Inpatient visits = 0.26 (0.83)  ER visits = 0.57 (1.46)  Clinic visits = 0.09 (1.06)  Office visits = 1.52 (3.95)  Other/unknown visits = 3.82 (24.05)  *Mean (SD) annual all-cause cost/charges:*  Prescription drug costs = $4,657 (14,716)  All medical services = $51,740 (209,481)  Outpatient visits = $11,558 (34,668)  Inpatient visits = $2,743 (25,755)  ER visits = $2,795 (8,271)  Clinic visits = $6,626 (78,835)  Office visits = $5,281 (59,969)  Other/unknown visits = $22,738 (174,142)  *Annual epilepsy-related costs/charges, mean (SD):*  All epilepsy-related prescriptions = $2,378 (10,487)  ASMs = $2,324 (10,414)  Rescue medications = $54 (361)  EVE = $0 (0)  All epilepsy-related medical services = $11,607 (83,033)  Outpatient visits = $3,961 (14,874)  Inpatient visits = $710 (5,508)  ER visits = $958 (2,881)  Clinic visits = $38 (834)  Office visits = $874 (4,035)  Other/unknown visits = $5,066 (80,936) |
| Hsieh 2013 [78] | USA | NA, 2002–2012 | Children and adults (aged 3–35 years) with TSC and epilepsy – IS persisting past age 2 | 6 | Number of drugs used for epileptic spasms, range = 1–3 |
|  |  |  | Children and adults (aged 3–35 years) with TSC and epilepsy – IS recurring after age 2 | 6 | Number of drugs used for epileptic spasms, range = 1–4 |
|  |  |  | Children and adults (aged 3–35 years) with TSC and epilepsy – IS with no history of IS | 4 | Number of drugs used for epileptic spasms, range = 1–3 |
| Husain 2000 [79] | USA | NA, 1987–1995 | Children with TSC and West Syndrome^‡^ – <50% reduction in seizures | 7 | Number of drugs prescribed, range = 4–8 |
|  |  |  | Children with TSC and West Syndrome^‡^ – >50% reduction in seizures | 10 | Number of drugs prescribed, range = 3–8 |
| Lennert 2013 [80] | USA | NA, 1973–2010 | Children and adults (mean age at diagnosis 3.1 years, range 0–50 years) with TSC and epilepsy | 95 | *Costs in first few years following diagnosis:*  Hospitalisation: 83.2% of patients, 0.5 PPY, 6.22 days  Surgery: 30.5%  Intensive care unit stay: 22.1%, 0.06 PPY, 3.26 days  ≥3 testing procedures: 90.5%  Rehabilitation services: 43.2% |
| Reaven 2019 [81] | USA | USD (2017 rates; 2010–2015 data) | Children and adults (aged 0–63 years) with TSC and epilepsy | 2,766 | ≥1 medically treated seizure event: 13.7–28.0%  Events during 2-year post index: 1.9–2.2  Cost per event: $5,335–9,672  All-cause costs PPPY: $42,997–48,330 |
| Rentz 2015a [41] | USA | NA, 2012 | Adults (mean age 38 years) and children (mean age 7.1 years) with TSC (24% of adults had epilepsy; 77% of children had epilepsy) | 676 | Physician visits in last year PPPY: 22.0  Procedures/tests in last year PPPY: 9.3  ER visits in last year PPPY: 2.5  Hospital admissions in last year PPPY: 2.5 |
| Rentz 2015b [82] | USA | NA, 2012 | Children and adults (aged <18 and ≥18 years) with TSC (adults mean age 42.3 years; 52% had epilepsy; children mean age 6.9 years; 78% had epilepsy) | 99 adults  176 children | Visited physician in last year: 98.0% adults; 98.9% children  Procedures or tests in last year: 90.9% adults; 97.7% children  Mean (SD) number of procedures or tests per year: 6.0 (8.0) both age groups  Number of physician visits per year: 16.0 (19.0) both age groups  Hospitalisation in last year: 35.4% adults; 37.5% children |
| Skalicky 2015 [83] | USA | NA, 2012 | Children and adults (aged <18 and ≥18 years) with TSC and SEGA (mean age 25.5 years; 65% had epilepsy) | 69 adults  47 children | Physician visit in past year: 100% adults; 95.7% children  Procedures or tests in past year: 97.1% adults; 95.7% children  Hospitalisation in last year: 47.8% adults; 34.0% children  ER visit in last year: 42.0% adults; 34.0% children  Days in hospital: 5.5 adults; 5.2 children |
| Skalicky 2018 [84] | USA | USD, 2012 | Children and adults (aged <18 and ≥18 years) with TSC (adults mean age 38 years; children mean age 7.1 years) | 401 adults  275 children | Annual ER visits: $500 adults vs $400 children (P<0.0001)  Monthly medical tests and procedures: $1,200 adults vs $300 children (P<0.0001)  Monthly alternative treatments: $680 adults vs $100 children (P=0.0008)  Monthly medications: $600 adults vs $150 children (P<0.0001)  Lifetime cost from surgeries: $4,000 adults vs $1,000 children (P=0.0004)  Monthly doctor’s visits: $500 adults vs $400 children  Monthly medical expenses related to hospital stays: $1,650 adults vs $1,200 children  Monthly non-medical expenses related to hospital stays: $1,000 adults vs $475 children |
| Stockl 2019 [85] | USA | NA | Patients with TSC-associated epilepsy who had acute hospitalisation^‡^ | 928 | Epilepsy-related hospitalisation = 26.0% |
| Sun 2015 [86] | USA | USD, 2000–2011 | Patients (aged ≤35 years) with TSC and surgical resection of SEGA (mean age 11.6; 91% had epilepsy) | 47 | Mean medical costs PPY: $8,543 pre-surgery and $85,397 post-surgery  Post-surgery inpatient care: $67,792  Post-surgery outpatient care: $8,024  Post-surgery medication cost: $1,038 |
| Deverell 2022 [87] | Australia | AUD, 2016 | Children with TSC (median [range] 7 [1–12] years for 15 children with rare diseases; 10 with TSC) | 10 | OOP expense <$5,000 for child’s lifetime, cost per year of life = <$5,000  OOP expense $5,001–10,000 for child’s lifetime, cost per year of life = $1,250–2,500  OOP expense $10,001–15,000 for child’s lifetime, cost per year of life = $1,818–2,727  OOP expense $20,001–25,000 for child’s lifetime, cost per year of life = $2,857–3,571  OOP expense $25,001–50,000 for child’s lifetime, cost per year of life = $1,190–2,381  OOP expense $75,001–100,000 for child’s lifetime, cost per year of life = $9,375–12,500  *Total OOP expenses over 6 months:*  Visits to specialist = $394  Visits to GP = $0  Visits to allied health professionals = $993  *Prospective OOP expenses over 6 months:*  Health service visits = $1,387  Hospital admissions = $0  Prescription medication = $1,042  non-prescription medication = $662  Health-related consumables = $1,189  Equipment = $0  Travel-related costs = $196  Special programmes = $1,143  Total costs = $5,289  *Projected OOP expenses over 1 year:*  Health service visits = $2,774  Hospital admissions = $0  Prescription medication = $2,084  Non-prescription medication = $1,324  Health-related consumables = $2,378  Equipment = $0  Travel-related costs = $392  Special programmes = $2,286  Total costs = $10,578 |
| Chu 2020 [88] | China (Hong Kong) | USD, 2016 | Children and adults (aged 5 months to 90 years) with TSC (71% had epilepsy) | 304 | *Mean costs:*  Hospital admission costs (PPPY) = $5,819  ICU stay cost (PPY) = $352  Cost of A&E visits (PPY) = $116  Cost of outpatient visits (PPY) = $1,414  A&E service charge per attendance = $158  ICU service charge per day = $3139  Outpatient service charge per attendance = $153  Inpatient service charge per day = $621  Total A&E costs in 10 years = $353,200  Total ICU costs over 10 years = $1,030,450  Total outpatient costs over 10 years = $4,297,910  Total inpatient costs over 10 years = $17,688,656 |
| Jansen 2017 [49] | Europe (6 countries) | NA | Children and adults with TSC (49% had epilepsy)^‡^ | 45 adults  66 children | Extra care assistance at home: 28.8% children  No health insurance: 42.2% adults; 25.8% children  Social services: <23%  TSC specialist: 77.8% adults; 54.5% children  Adult care access: 31.0%  No TSC-related visits in last year: 40.5% |
| Jansen 2020 [89] | Europe (7 countries) | NA | Children, adolescents, and adults (aged <11 years, 11–<18, and ≥18 years) with TSC (aged 3–72 years; 47% had epilepsy) | 55 adults  88 children and adolescents | Public/private insurance: 52.9% adolescents and 42.1% adults  TSC specialist: 70.6% adolescents and 73.7% adults  TSC clinic access: 76.3% adults  ≥3 physicians: 39.5% adults |
| Fagnani 2022 [90] | France | Euro, 2018 | Children and adults (aged 9–>70 years) with TSC (67% had epilepsy) | *2936* | *Mean annual healthcare costs:*  Total for TSC = €9,790  Inpatient costs = €3,710  Outpatient costs = €6,080  Medication costs = €3,575  Total for TSC with epilepsy = €11,413  Inpatient costs for TSC with epilepsy = €4,208  Outpatient costs for TSC with epilepsy = €7,205  Medication costs for TSC with epilepsy = €4,518  Total for TSC without epilepsy = €6,382  Inpatient costs for TSC without epilepsy = €2,665  Outpatient costs for TSC without epilepsy = €3,717  Medication costs for TSC without epilepsy = €1,595  Proportion of medication costs attributed to EVE for patients with TSC and epilepsy = 70%  Proportion of medication costs attributed to ASMs for patients with TSC and epilepsy = 12% |
| Grau 2021 [29] | Germany | Euro, 2019 | Children and adults with TSC  (aged 0.7–22 years; 92% had epilepsy) | *184* | *Mean costs over 3 months:*  Drug treatment = €2,658  Hospitalisation = €1,027  mTOR inhibitors = €2,309  ASMs = €260  Over-the-counter drugs and supplements = €11  Emergency medication = €5  Ancillary treatment = €407  Cost of nursing care = €1,163  Family member: informal care = €20.80  Family care: supervision = €27.60  Rehabilitation = €27  Emergency service use = €23  Specific diets = €22  Transport = €9  Co-payments for therapies = €125  Other co-payments = €15  Total direct costs: mTOR inhibitors = €2,310.30^§^  Total direct costs: hospitalisations = €1,031^§^  Total direct costs: ancillary therapies = €410.50^§^  Total direct costs: outpatient treatment = €348.40^§^  Total direct costs: ASMs = €262.50^§^  Total direct costs: diagnostics = €162.30^§^  Total direct costs: auxiliary material = €138.40^§^  Total direct costs: other drugs = €90.70^§^  Total direct costs: other costs = €85.90^§^  Total direct costs = €4,949  *Mean costs over 1 year:*  Total direct costs = €19,796  Medication = €10,632  mTOR inhibitors = €9,236  ASMs = €1,040  Other prescription drugs = €288  Over-the-counter drugs and supplements = €44  Emergency medication = €20  Hospitalisation = €4,108  Ancillary therapies = €1,628  Cost of nursing care = €4,652  Outpatient treatment = €1,384  Diagnostics = €624  Auxiliary material = €552  Rehabilitation = €108  Emergency service use = €92  Specific diets = €88  Transport costs = €36  Co-payments for therapies = €500  Other co-payments = €60  *Mean medication costs over 3 months:*  Medication costs: all patients = €260  Medication costs: no ASMs = €0  Medication costs: monotherapy = €158  Medication costs: 2 ASMs = €283  Medication costs: ≥3 ASMs = €736  OXC = €140  VGB = €267  LTG = €53  VPA = €39  LEV = €105  LCM = €619  CLB = €78  TPM = €128  BRV = €450  ESM = €79  Sulthiame = €64  CBZ = €47  PHT = €20  RFM = €799  ZNS = €243  Paediatric or adolescent patients with at least 1 hospital admission due to TSC = 22.3%  Number of admissions in 3 months = 49  Mean length of hospital stay = 6.04 days  Admission due to seizure = 23 admissions  Admission due to diagnostics = 22 admissions  Admission for other TSC reason = 4 admissions |
| Strzelczyk 2021b [19] | Germany | Euro, 2007–2016 | Children and adults (aged 1–86 years) with TSC (36% had epilepsy) | 100 | *Mean costs (PPY):*  Annual cost of healthcare = €6,139  Total costs = €6,139  Inpatient costs = €1,807  TSC-related costs = €528  Outpatient costs = €749  Medication costs = €2144  ASMs = €161  Services and devices = €942  Special equipment = €121  Other physical therapies = €37  Home nursing care = €31  Physiotherapy = €25  Transport for medical needs = €24  Other costs = €704  Dialysis = €230  Sick pay = €268  Hospitalisation rate = 0.5  LOS = 5.9 days  Hospitalisation rate due to TSC = 0.2  LOS because of TSC = 1.6 days |
|  |  |  | Children and adults with TSC and epilepsy^‡^ | 29 | *Mean costs (PPY):*  Total costs = €9,091  Inpatient costs = €2,356  TSC-related costs = €1,263  Outpatient costs = €1,053  Medication costs = €3,819  ASMs = €465  Services and devices = €1,480  Special equipment = €203  Other physical therapies = €77  Home nursing care = €19  Physiotherapy = €36  Transport for medical needs = €48  Other costs = €1,097  Dialysis = €325  Sick pay = €58  Hospitalisation rate = 0.7  LOS = 8.4 days  Hospitalisation rate due to TSC = 0.4  LOS because of TSC = 4.3 days  Cost of EVE (per prescription) = €6,205 |
|  |  |  | Children and adults with TSC without epilepsy^‡^ | 71 | *Mean costs (PPY):*  Total costs = €4,583  Inpatient costs = €1,517  TSC-related costs = €146  Outpatient costs = €589  Medication costs = €1,261  ASMs = €0  Services and devices = €658  Special equipment = €78  Other physical therapies = €15  Home nursing care = €38  Physiotherapy = €19  Transport for medical needs = €11  Other costs = €496  Dialysis = €179  Sick pay = €379  Hospitalisation rate = 0.4  LOS = 4.6 days  Hospitalisation rate due to TSC = 0.01  LOS because of TSC = 0.2 days  Cost of EVE (per prescription) = €5,238 |
|  |  |  | Children and adults with TSC and epilepsy who were prescribed rescue medication^‡^ | 28 | *Mean costs/rate/LOS (PPY):*  Healthcare = €20,793  Medication = €10,879  Inpatient care = €4,931  Services and devices = €3,296  Outpatient care = €1,687  ASMs = €823  Hospitalisation rate = 1.7  LOS = 18.6 days |
| Zöllner 2021b [63] | Germany | Euro, 2019 | Adults (aged 18–78 years) with TSC (73% had epilepsy) – total population | 192 | *Mean (SD) direct costs over 3 months*  Total direct costs = €6,452 (7,584)  Medication = €4,953 (6,854)  mTOR inhibitors = €4,358 (6,520)  ASMs = €415 (1,962)  Other prescription drugs = €132 (385)  Over-the-counter drugs and supplements = €41 (100)  Emergency medication = €7 (36)  Hospitalisation = €518 (1,691)  Outpatient treatment = €467 (1,156)  Diagnostics = €155 (242)  Ancillary therapies = €125 (307)  Auxiliary material = €49 (253)  Rehabilitation = €40 (410)  Emergency service use = €44 (217)  Specific diets = €9 (97)  Transport costs = €5 (17)  Co-payments for therapies = €39 (177)  Other co-payments = €48 (183)  *Mean direct costs over 1 year:*  Total direct costs = €25,808  Medication = €19,812  mTOR inhibitors = €17,432  ASMs = €1,660  Other prescription drugs = €528  Over-the-counter drugs and supplements = €164  Emergency medication = €28  Hospitalisation = €2,072  Outpatient treatment = €1,868  Diagnostics = €620  Ancillary therapies = €500  Auxiliary material = €196  Rehabilitation = €160  Emergency service use = €176  Specific diets = €36  Transport costs = €20  Co-payments for therapies = €156  Other co-payments = €192  *Mean (SD) prescription costs over 3 months:*  Medication costs = €415 (1,962)  No ASMs = €0  Monotherapy = €222 (400)  2 ASMs = €327 (317)  ≥3 ASMs = €1,965 (4,979)  LTG = €90 (93)  VPA = €62 (21)  OXC = €226 (99)  LEV = €161 (73)  LCM = €894 (616)  TPM = €149 (37)  ZNS = €1087 (745)  CBZ = €35 (24)  PER = €308 (120)  BRV = €508 (175)  Sulthiame = €320 (284)  VGB = €397 (137)  PHT = €26 (7)  PRM = €38 (23)  PB = €62 (47)  RFM = €759 (421)  CLB = €68 (36)  GPN = €78 (101) |
|  |  |  | Adults (aged 18–78 years) with TSC (73% had epilepsy) – subpopulations by sex, age, ASM use, epilepsy status and comorbidities | 192 | *Mean (SD) total costs over 3 months:*  Male = €6,173 (8,156)  Female = €6,714 (7,036)  Aged 18–29 years = €8,067 (8,419)  Aged 30–39 years = €5,849 (6,700)  Aged >40 years = €4,102 (6,154)  ≥2 ASMs = €9,237 (8,639)  0–1 ASMs = €4,587 (6,155)  mTOR inhibitors = €14,473 (5,641)  No mTOR inhibitors = €1,746 (3,548)  Recurrent seizures = €9,082 (8,930)  Seizure-free >1 year or no seizures = €4,729 (5,997)  Has epilepsy = €7,195 (8,106)  Does not have epilepsy = €4,451 (5,545)  Has structural brain disorder = €7,353 (8,041)  Does not have structural brain disorder = €4,607 (6,211)  Has psychiatric disorder = €8,034 (8,083)  Does not have psychiatric disorder = €4,837 (6,703)  Has heart and circulatory manifestations = €8,180 (7,764)  Does not have heart and circulatory manifestations = €4,687 (7,007)  Has kidney and urinary tract manifestations = €7,311 (7,419)  Does not have kidney and urinary tract manifestations = €4,013 (7,591)  Has skin manifestations = €6,704 (7,669)  Does not have skin manifestations = €1,873 (3,666)  Has lung manifestations = €8,894 (7,928)  Does not have lung manifestations = €6,136 (7,505)  Has other manifestations = €7,856 (7,352)  Does not have other manifestations = €5,552 (7,625)  1–3 manifestations = €2,652 (5,369)  4 manifestations = €6,488 (7,796)  5 manifestations = €6,353 (7,158)  6 manifestations = €8,080 (8,022)  7–8 manifestations = €11,977 (7,402)  No disability or ≤60% disability = €4,395 (6,823)  70–100% disability = €7,580 (7,768) |
| Marques 2019 [91] | International | NA/NR | Children and adults with TSC^‡^ enrolled in TOSCA (patients in TOSCA were aged 0–71 years; 85% had epilepsy) | 132 visits  143 hospitalisations | No healthcare visits for TSC = 31.8%  At least 1 healthcare visit for TSC = 52.3%  1 healthcare visit, TSC related = 13.6%  2 healthcare visits, TSC related = 16.7%  ≥3 healthcare visits, TSC related = 22.0%  No hospitalisations = 70.6%  At least 1 hospitalisation = 28.7%  1 hospitalisation = 17.5%  2 hospitalisations = 4.9%  ≥3 hospitalisations = 6.3% |
| Waltereit 2021 [92] | International | NA/NR | Patient survey respondents^‡^  (HCPs, and caregivers/families of patients with TSC) | 259 | Never had a neuropsychiatric assessment = 46.0%  Screened for TAND yearly = 9.9%  Screened for TAND once every 3 years = 3.8%  Screened for TAND once = 34.6%  Screened for TAND twice per year = 3.0%  Screened for TAND every 3 months = 2.7% |
|  |  |  | Neurologists^‡^ | 59 | Screened for TAND never = 1.7%  Screened for TAND rarely = 0%  Screened for TAND sometimes = 25.4%  Screened for TAND once per year = 57.6%  Screened for TAND only when requested = 15.3% |
| Vignoli 2013 [14] | Italy | NA, 2000–2012 | Children and adults with TSC and epilepsy (aged 1–74 years, mean 31.6 years) – seizure-free | 57 | Monotherapy = 56.1%  Polytherapy = 31.6% |
|  |  |  | Children and adults with TSC and epilepsy (aged 1–74 years, mean 24.1 years) – refractory seizures | 59 | Monotherapy = 15.3%  Polytherapy = 84.7% |
| Overwater 2017 [23] | Netherlands | NA/NR | Children with TSC (median age 8.2 years; 86% had epilepsy) | 102 | Median (IQR) ASMs used = 1 (0–3) |
|  |  |  | Children with TSC and epilepsy (median age 8.0 years) | 88 | Median (IQR) ASMs used = 2 (1–4) |
| Vergeer 2019 [60] | Netherlands | NA, 1990–2015 | Adults (median age 42–46 years) with TSC and epilepsy – refractory epilepsy for 100% of follow-up | 49 | Hospital visit = 34.7%  ICU visit = 6.1%  Neurologist visit = 67.3%  EEG = 28.6%  MRI = 12.2%  CT scan = 6.1% |
|  |  |  | Adults (median age 42–46 years) with TSC and epilepsy – refractory epilepsy for 50–99.9% of follow-up | 81 | Hospital visit = 49.4%  ICU visit = 1.2%  Neurologist visit = 95.1%  EEG = 67.9%  MRI = 19.8%  CT scan = 24.7% |
|  |  |  | Adults (median age 42–46 years) with TSC and epilepsy – refractory epilepsy for 0.1–50% of follow-up | 44 | Hospital visit = 36.4%  ICU visit = 2.3%  Neurologist visit = 75.0%  EEG = 61.4%  MRI = 22.7%  CT scan =9.1% |
|  |  |  | Adults (median age 42–46 years) with TSC and epilepsy – refractory epilepsy for 0% of follow-up | 63 | Hospital visit = 14.3%  ICU visit = 3.2%  Neurologist visit = 42.9%  EEG = 31.7%  MRI = 6.3%  CT scan = 6.3% |
| Lesniowska 2019 [93] | Poland | Euro, NR | Patients with TSC and complications, including epilepsy^‡^ | NR | Total cost nationally = €2,100,000–€3,300,000 |
|  |  |  | Patients with TSC and complications, including epilepsy^‡^ | NR | Reduction of TSC costs after implementation of preventive epileptic treatment = €2,700,000 |
| Welin 2017 [24] | Sweden | NA, 2004–2014 | Children and adults with TSC and refractory epilepsy (aged 0–>65 years) | 127 | *Mean visits per year:*  Outpatient visits = 6.22  Inpatient days = 4.83  Outpatient visits with epilepsy = 2.80  Inpatient days with epilepsy = 3.59 |
|  |  |  | Children and adults with TSC and non-refractory epilepsy (aged 0–>65 years) | 259 | *Mean visits per year:*  Outpatient visits = 3.94 per year  Inpatient days = 2.47 per year  Outpatient visits with epilepsy = 1.08 per year  Inpatient days with epilepsy = 1.31 per year |
| Crawford 2015 [16] | UK | NA, 1997–2012 | Children and adults with TSC (71% had epilepsy)^‡^ | 341 | *Mean outpatient visits (min, max) to a neurologist or paediatrician, PPY:*  Children: 2.8 (0.1, 13.4)  Adults: 0.7 (0.1; 7.3)  57% patients with epilepsy had ≥1 hospitalisation due to epilepsy (min. 0.1; max. 2.4 admissions PPY)  73% of hospitalisations presented via the ER |
| Demuth 2014 [94] | UK | NA, 1997–2012 | Children and adults (aged <18 and ≥18 years) with TSC (median age 14.4 years at first health care encounter; 53–62% had neurological manifestations, including epilepsy) | 341 | *Mean (SD) annual rate of health care encounters, PPY:*  Adults: 15.6 (15.3)  Children: 13.7 (14.3)  *Mean (SD) annual rates of encounters by care setting:*  GP: 12.9 (14.2) adults vs 10.1 children (3.1)  Inpatient: 0.6 (1.0) adults vs 1.0 (1.6) children  ER admission: 0.3 (0.7) adults vs 0.4 (0.8) children  Outpatient: 2.8 (3.0) adults vs 3.9 (3.3) children  *Mean (SD) number of surgical procedures and tests:*  Adults: 0.3 (0.4)  Children: 0.2 (0.4)  *MRI or CT scans:*  Adults: 26%  Children: 34% |
| Kingswood 2016 [95] | UK | GBP, 1987–2013 | Adults (aged ≥18 years) with renal TSC with renal manifestation only | 2 | Cost per renal manifestation only = £3,600 |
|  |  |  | Patients with renal TSC with 1 additional manifestation | 9 | Extra cost per additional 1 manifestation = £4,058 |
|  |  |  | Patients with renal TSC with 2 additional manifestations | 16 | Extra cost per additional 2 manifestations = £2,475 |
|  |  |  | Patients with renal TSC with 3 additional manifestations | 32 | Extra cost per additional 3 manifestations = £11,207 |
|  |  |  | Patients with renal TSC with 4 additional manifestations | 20 | Extra cost per additional 4 manifestations = £23,931 |
| Shepherd 2017 [51] | UK | GBP, 1987–2013 | Children and adults (mean age 26.8 years) with TSC with epilepsy |  | Brain surgery over entire history: 12%  Average brain surgery procedures over 3 years: 0.1  Routine electroencephalography and MRI: 30%  Routine EEG over 3 years: 10.9% adults; 46.9% children  Routine MRI over 3 years: 21.1% adults; 58.0% children  GP visits over 3 years: 60.8  (50.5–71.2)  Inpatient admissions over 3 years: 3.4 (2.1–4.7)  Clinical costs over 3 years: £14,335 |
| Karakas 2020 [96] | Unclear | NA, 2013–2018 | Children (aged 1–17 years) with TSC and refractory epilepsy who had sEEG monitoring and MRI guided laser ablation | NR | Hospital length of stay (range) during ablation = 14 days (5–45) |

^†^Comparator population could refer to patients with TSC alone, individuals representing a healthy population, patients with TSC and different types of seizures, or patients with TSC-associated epilepsy and well-controlled seizures. ^‡^Population age not defined. ^§^Data extracted from figure using webplotdigitizer: <https://apps.automeris.io/wpd/>

A&E, accident and emergency; ASM, antiseizure medication; AUD, Australian Dollars; BRV, brivaracetam; CBZ, carbamazepine; CI, confidence interval; CLB, clobazam; CT, computed tomography; EEG, electroencephalography; ER, emergency room; ESM, ethosuximide; EVE, everolimus; GBP, Great Britain Pound; GP, general practitioner; GPN, gabapentin; HCP, healthcare professional; HRU, healthcare resource utilisation; ICU, intensive care unit; IQR, interquartile range; IRR, incidence rate ratio; IS, infantile spasm; LCM, lacosamide; LEV, levetiracetam; LOS, length of stay; LTG, lamotrigine; MRI, magnetic resonance imaging; mTOR, mammalian target of rapamycin; NA, not applicable; NR, not reported; OOP, out-of-pocket; OXC, oxcarbazepine; PB, phenobarbital; PER, perampanel; PHT, phenytoin; PPPY, per patient per year; PPY, per person year; PRM, primidone; RFM, rufinamide; SD, standard deviation; sEEG, stereoelectroencephalography; SEGA, subependymal giant astrocytoma; TAND, tuberous sclerosis complex-associated neuropsychiatric disorders; TOSCA, TuberOus SClerosis registry to increase disease Awareness; TPM, topiramate; TSC, tuberous sclerosis complex; USD, US dollars; VGB, vigabatrin; VPA, valproate; ZNS, zonisamide

# Online additional table S4. Indirect costs and resource use of TSC and epilepsy (n=15 studies)

| **Study** | **Country** | **Currency and year** | **Study patient population –**  **subgroup population** | **N** | **Indirect costs and resource use** |
| --- | --- | --- | --- | --- | --- |
| Frost 2013 [97] | USA | NA | Adults with TSC and epilepsy (aged 0–70 years for all patients with TSC) – employed patients | 28 | Working week missed = 18.0%  Performance impairment = 40.0% |
|  | USA | NA | Adults with TSC and epilepsy (aged 0–70 years for all patients with TSC) – employed caregivers | 69 | Working week missed = 9.0%  Performance impairment = 30.0% |
| Rentz 2015b [82] | USA | NA | Children and adults with TSC (adults: mean age 42.3 years; 52% had epilepsy; children: mean age 6.9 years; 78% had epilepsy) | 275 | *Caregiver outcomes:*  Highest reached education level:  Elementary/primary school = 1.1%  High school = 29.8%  College = 45.1%  Postgraduate = 20.4%  Technical/vocational/other = 3.6%  Employed full-time = 53.1%  Employed part-time = 11.3%  Homemaker = 18.9%  Student = 2.2%  Unemployed = 7.3%  Retired = 4.0%  Other employment status = 3.3% |
| Skalicky 2018 [84] | USA | NA | Adults (aged >18 years, 18–69 years) with TSC – patient outcomes | 334 | Employed = 70.7%  In school = 16.8%  *Mean WPAI scores:*  Work absenteeism = 15.0%  Work presenteeism = 58.0%  Work productivity loss = 63.0%  School absenteeism = 27.0%  School presenteeism = 60.0%  School productivity loss = 67.0% |
|  | USA | NA | Adults (aged >18 years, 18–69 years) with TSC – caregiver outcomes | 275 | Employed = 62.2%  In school = 4.7%  *Mean WPAI scores:*  Work absenteeism = 11.0%  Work presenteeism = 38.0%  Work productivity loss = 42.0%  School absenteeism = 11.0%  School presenteeism = 37.0%  School productivity loss = 40.0% |
| Skalicky 2015 [83] | USA | NA | Adults (aged >18 years) with TSC and SEGA (aged 19–60 years; 65% had epilepsy) | 69 | Employed = 23.0%  Work week missed = 18.0%  Work productivity = 37.0%  *Median time/costs:*  Time travelling to appointments (over the past 12 months) = 20 hours  OOP office visits cost = $520  OOP A&E visits cost = $2,750  OOP monthly medication cost = $129  OOP surgery cost = $3,000 |
|  | USA | NA | Children (aged ≤18 years) with TSC and SEGA (aged 1–18 years; 65% had epilepsy) | 47 | *Median time/costs:*  Time travelling to appointments (over the past 12 months) = 15 hours  OOP A&E visits cost = $225  OOP cost hospital stays = $1,500  OOP monthly medication cost = $80  OOP alternative treatment = $0 |
| Deverell 2022 [87] | Australia | AUD | Children with TSC (median [range]  7 [1–12] years for 15 children with rare diseases; 10 with TSC) | 13 | Reduced or stopped employment to care for affected child = 61.5%  Families reporting inability to save money or break even = 46.2%  Families reporting spending more money than they earn = 15.4%  Families reporting ability to save some money each week = 38.5%  Annual PHI premium costs for families of affected children = median $3,264  Families reporting that health insurance covered a small proportion of their child’s healthcare costs = 45.5%  Families reporting that health insurance covered some of their child’s healthcare costs = 36.4%  Families reporting that health insurance covered most of their child’s healthcare costs = 18.2%  Families who felt their PHI premiums were poor value for money = 72.7%  Families reporting OOP expense for travel and accommodation when visiting health services = 84.6%  Families reporting OOP expenses for visits to specialist doctors = 76.9%  Families reporting OOP expenses for non-prescription medicines = 76.9%  Families reporting OOP expenses for prescription medicines = 69.2%  Families reporting OOP expenses for visits to allied health professionals = 53.8%  Families reporting OOP expenses for visits to GP = 38.5%  Families reporting OOP expenses for operations/procedures = 23.1%  Families reporting OOP expenses for hospital admissions = 15.4%  Families reporting OOP expenses for other costs = 15.4% |
| Valentim 2010 [98] | Brazil | 2008 BRL | Children and adults with TSC and epilepsy – adult- or childhood-onset epilepsy^†^ | NR | *Epilepsy-related costs per patient:*  Mean discounted annual productivity cost = R$1,970 per patient  Discounted total productivity cost = R$97,882 per patient  Discounted total productivity cost of TSC epilepsy in Brazil = R$1,568,965,961  Productivity loss with adult-onset epilepsy = 30 years  Patient productivity loss with childhood-onset epilepsy = 47 years  Caregiver productivity loss = 65 years |
| Jansen 2017 [49] | Europe | NA | Children and adults with TSC^†^ (adults: 49% had epilepsy; children: 41% had epilepsy) | 45 | Impact on patient career/education = 55.6%  Impact on primary caregiver career/education = 50.0%  Impact on other family members’ career/education = 26.7% |
| Jansen 2020 [89] | Europe | NA | Adults (aged ≥18 years) with TSC self-reported (aged 18–72 years; 47% had epilepsy) | 38 | TSC has an impact on career/education of self = 42.1%  TSC has an impact on career progression/promotion = 25.0%  TSC impacted choice of career = 25.0%  TSC impacted loss of employment = 31.3%  TSC impacted choice of work (part-time instead of full-time) = 31.3%  TSC impacted education level received = 37.5%  Current employment (full- or part-time) = 52.6%  Patients unable to work due to TSC = 10.5%  Patients unable to work not due to TSC = 7.9%  Patients who are currently students = 5.3%  Patients who are currently homemakers = 18.4%  Patients receiving disability living allowance = 21.1%  Patients receiving caregiver allowance = 0%  Patients receiving social worker assistance = 0%  Patients receiving social services support = 0% |
|  | Europe | NA | Children (aged <11 years) and adolescents (aged 11 to <18 years) with TSC  (aged 3–17 years; 47% had epilepsy) | 71 | *Caregiver outcomes:*  TSC has an impact on career/education of self or caregiver = 66.2%  TSC has an impact on career progression/promotion caregivers = 36.2%  TSC impacted choice of career of caregivers = 34%  TSC impacted loss of employment of caregivers = 21.2%  TSC impacted choice of work (part-time instead of full-time) = 53.2%  Current education level received of caregivers = 6.4%  TSC affects employment of caregivers (full- or part-time) = 66.2%  Caregivers unable to work due to TSC = 11.3%  Caregivers unable to work not due to TSC = 11.3%  Caregivers who are currently students = 0%  Caregivers who are currently homemakers = 14.1%  TSC had impact on child: child is in mainstream education = 60.6%  TSC had impact on child: children receiving support in class = 43.7%  TSC had impact on additional support causing child additional problems = 18.3%  Patients receiving disability living allowance = 54.9%  Patients receiving caregiver allowance = 12.7%  Patients receiving social worker assistance = 8.5%  Patients receiving social services support = 4.2% |
| Bar 2019 [64] | France | NA | Adults (aged ≥18 years) with TSC  (aged 18–55 years) who developed epilepsy before age 16 years | 60 | Stable employment = 37.5%  Salary below income threshold = 65.0% |
| Grau 2021 [29] | Germany | Euro, February–July 2019 | Children and adults (aged 1–22 years) with TSC (92% had epilepsy) – total population  Children aged >16 years were considered of working age | 111 | *Mean costs over 3 months:*  Total indirect costs = €3,184 (95% CI 2,533, 3,811)  Total maternal indirect costs €2,813 (95% CI 2,221, 3,394)  Total paternal indirect costs = €372 (95% CI 193, 586)  Mothers who had to quit work = 13.0%  Fathers who had to quit work = 1.1%  Mothers who had to reduce work hours = 26.6%  Fathers who had to reduce work hours = 3.8%  Mothers who missed work days = 22.3%  Fathers who missed work days = 22.3%  Cost of mothers quitting work = €1,466  Cost of fathers quitting work = €122  Cost of mothers’ reduced hours = €1,109  Cost of fathers’ reduced hours = €86  Cost of mothers’ missed work days = €238  Cost of fathers’ missed work days = €163  Costs for adolescents (missing school/work) = €1,002 (95% CI 103, 2,005)  *Mean costs over 1 year:*  Total indirect costs = €12,736  Total maternal indirect costs (mean, over 1 year) = €11,252  Total paternal indirect costs = €1,488  Maternal cost of quitting work (mean, over 1 year) = €5,864  Maternal cost of reducing hours = €4,436  Maternal cost of days off due to TSC = €952  Paternal cost of quitting work = €488  Paternal cost of reducing hours = €344  Paternal cost of days off due to TSC = €652  Total parental indirect costs = €12,736 |
|  | Germany | Euro, February–July 2019 | Children and adults (aged 1–22 years) with TSC (92% had epilepsy) – subpopulations by sex, age, ASM use, epilepsy status and TSC manifestations  Children aged >16 years were considered of working age | 184 | *Mean (SD) total indirect costs over 3 months:*  Male = €3,291 (4,063)  Female = €3,071 (4,612)  Aged 0–3 years = €4,396 (4,771)  Aged 4–10 years = €2,923 (4,635)  Aged 11–<22 years = €2,796 (3,687)  ≥2 ASMs = €4,172 (4,814)  0–1 ASMs = €2,153 (3,488)  mTOR inhibitors = €2,322 (3,795)  No mTOR inhibitors = €3,497 (4,476)  Recurrent seizures = €4,014 (4,768)  Seizure-free >1 year or no seizures = € 2,423 (3,744)  Has epilepsy = €3,351 (4,390)  Does not have epilepsy = €1,308 (3,051)  Has structural brain disorder = €3,369 (4,424)  Does not have structural brain disorder = €2,273 (3,742)  Has psychiatric disorder = €4,387 (4,883)  Does not have psychiatric disorder = €1,929 (3,233)  ≤60% disability = €1,544 (3,235)  70–100% disability = €3,998 (4,574)  1–2 manifestations = €1,593 (3,805)  3 manifestations = €1,637 (2,811)  4 manifestations = €3,475 (5,171)  5 manifestations = €3,087 (4,037)  6–7 manifestations = €4,337 (4,561) |
| Zöllner 2021b [63] | Germany | Euro, February–July 2019 | Adults (aged ≥18 years, range 18–78 years) with TSC (72.9% had epilepsy) – subpopulations by sex, age, ASM usage, epilepsy status, TSC manifestations, and employment status | 192 | *Mean (SD) indirect costs over 3 months:*  Total indirect costs = €3,174 (4,703)  Male = €2,796 (4,687)  Female = €3,529 (4714)  Aged 18–29 years = €2,171 (4,117)  Aged 30–39 years = €3,978 (5,081)  Aged ≥40 years = €4,192 (5,011)  ≥2 ASMs = €4,054 (5,131)  0–1 ASMs = €2,574 (4,308)  mTOR inhibitors = €3,335 (4,729)  No mTOR inhibitors = €3,077 (4,705)  Recurrent seizures = €3,859 (5,061)  Seizure-free >1 year or no seizures = €2,717 (4,412)  Has epilepsy = €3,284 (4,866)  Does not have epilepsy = €2,866 (4,242)  Has structural brain disorder = €3,106 (4,681)  Does not have structural brain disorder = €3,318 (4,784)  Has psychiatric disorder = €3,741 (5,110)  Does not have psychiatric disorder = €2,583 (4,182)  Has other manifestations = €3,711 (4,982)  Does not have other manifestations = €2,823 (4,499)  ≤60% disability = €1,483 (3,085)  70–100% disability = €4,074 (5,158)  1–3 manifestations = €1,982 (3,890)  4 manifestations = €2,676 (4,299)  5 manifestations = €4,816 (5,345)  6 manifestations = €3,169 (4,770)  7–8 manifestations = €3,844 (5,176)  Patients with TSC unable to work = €1,775 (4,110); estimated annual cost = €7,100  Patients with TSC with reduced hours = €514 (1,762); estimated annual cost = €2,056  Patients with TSC with unemployment = €355 (1,971)  Patients with TSC with unemployment = €355 (1,971); estimated annual cost = €1,420  Patients with TSC with early retirement = €296 (1,804); estimated annual cost = €1,184  Patients with TSC with days off work = €234 (1050); estimated annual cost = €936 |
| Marques 2019, incl. supplement [91] | International | NA | Children (aged 0–17 years) with TSC enrolled in TOSCA (patients in TOSCA were  aged 0–71 years; 85% had epilepsy) – patient outcomes | 88 | Not in mainstream school = 31.8%  Mainstream school = 64.8%   - Receiving special education = 64.9% - Receiving special programmes adequate to child’s condition = 45.6%   Disability allowance = 51.1%  Caregiver allowance = 11.4%  Psychological counselling = 13.6%  Social services = 4.5%  Social worker = 8.0%  Help completing benefit applications = 5.7% |
|  | International | NA | Children (aged 0–17 years) with TSC enrolled in TOSCA (patients in TOSCA were  aged 0–71 years; 85% had epilepsy) – caregiver outcomes | 88 | Employed = 65.9%  Unable to work = 9.1%  TSC impacted on professional career = 56.8% |
|  | International | NA | Adults (aged 18–71 years) with TSC enrolled in TOSCA (patients in TOSCA were  aged 0–71 years; 85% had epilepsy) | 55 | Disability allowance = 38.2%  Caregiver allowance = 0%  Psychological counselling = 5.5%  Social services = 3.6%  Social worker = 1.8%  Help completing benefit applications = 3.6%  Daily activity support = 20.0%  Employed = 41.8%  Unable to work due to TSC = 25.5%  TSC impacted on professional career = 50.9% |
| Vergeer 2019 [60] | Netherlands | NA | Adults (mean age 42–46 years) with TSC and epilepsy – refractory epilepsy for 100% of follow-up | 49 | Independent living = 0%  With caregiver = 10.2%  In group home = 63.3%  Group home and caregiver = 14.3% |
|  | Netherlands | NA | Adults (mean age 42–46 years) with TSC and epilepsy – refractory epilepsy for 50–99.9% of follow-up | 81 | Independent living = 14.8%  With caregiver = 14.8%  In group home = 37%  Group home and caregiver = 24.7% |
|  | Netherlands | NA | Adults (mean age 42–46 years) with TSC and epilepsy – refractory epilepsy for 0.1–50% of follow-up | 44 | Independent living = 9.1%  With caregiver = 22.7%  In group home = 43.2%  Group home and caregiver = 11.4% |
|  | Netherlands | NA | Adults (mean age 53 years) with TSC and epilepsy – refractory epilepsy for 0% of follow-up | 63 | Independent living = 23.8%  With caregiver = 19.0%  In group home = 44.4%  Group home and caregiver = 4.8% |
| Skrobanski 2023a [99] | UK | GBP, May–July 2021 | Children and adults (aged <18 and ≥18 years) with TSC (mean age 20 years; 94% had epilepsy) – caregiver respondents | 73 | Households who received professional social care = 40.7%  Mean (SD) number of hours per week who received private funded social care = 5.1 (10.8)  Mean (SD) number of hours per week who received state-funded social care = 20.5 (26.9)  Caregivers who required additional childcare = 12.3%  Mean (SD) yearly OOP costs for additional childcare = £1,357.70 (£1,945.3)  Mean (SD) spend on private funded care per week = £41.60 (£89.70)  Primary caregivers who stopped work completely = 45.0%  Primary caregivers who reduced their working hours = 51.0%  Primary caregivers who reduced working hours or changed job to accommodate caregiving needs = 22.0%  Mean (SD) number of hours per week missed by primary caregivers who worked due to caregiving = 7.8 (7.9) |
|  | UK | GBP, May–July 2021 | Children and adults (aged <18 and ≥18 years) with TSC (mean age 20 years; 94% had epilepsy) – partners of caregivers and other household members of individuals with TSC | NR | Primary caregivers’ partners who reduced working hours = 23.0%  Primary caregivers’ partners who changed jobs to accommodate caring responsibilities = 9.0%  Primary caregivers’ partners who stopped working completely = 9.0%  Other household members of working age who reduced their working hours = 20.0%  Mean (SD) number of hours per week missed by primary caregivers’ partners who worked due to caregiving = 3.9 (7.9)  Mean (SD) number of hours per week missed by other household members who worked due to caregiving = 1.8 (3.3) |
| Skrobanski 2023b [100] | UK | NA, May–July 2021 | Caregivers (aged ≥18 years) of children and adults (mean age 20 years) with TSC (94% had epilepsy) | 73 | Received care allowance = 35.9%  Received carer credit = 3.1%  Received other benefits but not care allowance or carer credit = 4.7%  Received no financial benefits = 56.3%  Used NHS services = 37.5% |
|  | UK | NA, May–July 2021 | Children and adults (mean age 20 years) with TSC (94% had epilepsy) | 71 | Receiving disability allowance = 81.7%  Receiving social services support = 29.6%  Receiving social worker support = 18.3%  Needed help completing benefit applications = 14.1%  Received psychological counselling = 7.0%  Received no financial, psychological or social support = 12.7% |

^†^Population age not defined

A&E, accident and emergency; ASM, antiseizure medication; AUD, Australian Dollars; BRL, Brazilian Real; GBP, Great Britain Pound; mTOR, mammalian target of rapamycin; NA, not applicable; NHS, National Health Service; OOP, out-of-pocket; PHI, private health insurance; SD, standard deviation; SEGA, subependymal giant cell astrocytoma; TOSCA, TuberOus SClerosis registry to increase disease Awareness; TSC, tuberous sclerosis complex; WPAI, Work Productivity and Activity Impairment

# Online additional table S5. Treatment patterns for subpopulations of patients with TSC-associated epilepsy (n=59 studies)

| **Study** | **Country** | **Year** | **Study patient population –**  **subgroup population** | **N** | **Treatment patterns** |
| --- | --- | --- | --- | --- | --- |
| Bauer 2016 [76] | USA | NR | Adults with TSC and epilepsy^†^ – seizure-free at last visit | 10 | Mean number of ASMs taken at last visit = 2.1  Mean number of ASMs taken throughout follow-up: 4.5 |
|  | USA | NR | Adults with TSC and epilepsy (mean age 34.8 years) – refractory epilepsy | 6 | Mean number of ASMs taken at last visit = 3.8  Mean number of ASMs taken throughout follow-up = 11.7 |
| Betts 2020 [77] | USA | 2019 | Children and adults with TSC (mean age at index date 25.3 years) – TSC-associated epilepsy | 2,028 | Mean (SD) number of ASMs = 2.1 (1.4)  Patients with ≥1 claim for an ASM, n (%):  Any ASM = 1,815 (89.5%)  LEV (ER) = 616 (30.4%)  LTG (ER and ODT) = 408 (20.1%)  OXC = 374 (18.4%)  CBZ = 343 (16.9%)  Divalproex sodium (ER) = 321 (15.8%)  CLB = 295 (14.6%)  TPM (ER) = 281 (13.9%)  LCM = 255 (12.6%)  VGB = 239 (11.8%)  CBZ (ER) = 209 (10.3%)  ZNS = 142 (7.0%)  GPN = 122 (6.0%)  VPA = 105 (5.2%)  RFM = 87 (4.3%)  PHT extended = 86 (4.2%)  PB = 74 (3.7%)  FBM = 54 (2.7%)  PER = 43 (2.1%)  PGB = 38 (1.9%)  PHT = 36 (1.8%)  BRV = 34 (1.7%)  ESL = 25 (1.2%)  ESM = 10 (0.5%)  CBD = <10 (<0.5%)  EVE = 203 (10.0%)  Patients with ≥1 claim for rescue medication = 387 (19.1%) |
|  | USA | 2019 | Children and adults with TSC (mean age at index date 25.3 years) – non-TSC-associated epilepsy | 10,140 | Mean (SD) number of ASMs = 1.3 (1.2)  Patients with ≥1 claim for an ASM, n (%):  ASMs = 7,218 (71.2%)  LEV (ER) = 3,112 (30.7%)  LTG (ER and ODT) = 1,330 (13.1%)  OXC = 970 (9.6%)  CZP = 1,145 (11.3%)  Divalproex sodium (ER) = 1,039 (10.3%)  CLB = 504 (5.0%)  TPM (ER) = 961 (9.5%)  LCM = 540 (5.3%)  VGB = 53 (0.5%)  CBZ (ER) = 390 (3.9%)  ZNS = 437 (4.3%)  GPN = 1,233 (12.2%)  VPA = 247 (2.4%)  RFM = 129 (1.3%)  PHT extended = 311 (3.1%)  PB = 283 (2.8%)  FBM = 56 (0.6%)  PER = 84 (0.8%)  PGB = 193 (1.9%)  PHT = 75 (0.7%)  BRV = 58 (0.6%)  ESL = 58 (0.6%)  ESM = 37 (1.4%)  CBD = <10 (<0.1%)  EVE = <10 (<0.1%)  Patients with ≥1 claim for rescue medication = 1,163 (11.5%) |
| Chu-Shore 2010 [7] | USA | NR | Children and adults with TSC and refractory epilepsy^†^ | 155 | Epilepsy surgery = 25.2%  VNS = 0.6% |
| Frost 2013 [97] | USA | NR | Children and adults with TSC and epilepsy (aged 0–70 years) | 176 | ASMs in past 6 months = 77.0%  1 ASM = 27.0%  2 ASMs = 31.0%  3 ASMs = 13.0%  ≥4 ASMs = 6.0% |
| Hsieh 2013 [78] | USA | NR | Children and adults (aged 3–35 years) with TSC and epilepsy – IS persisting past 2 years of age | 6 | Number of ASMs used for ES, range = 1–3  Number of ASMs used for IS, range = 1–8 (range) |
|  | USA | NR | Children and adults (aged 3–35 years) with TSC and epilepsy – IS recurrence after age 2 years | 6 | Number of ASMs used for ES, range = 1–4  Mean number of ASMs used for IS = 1 |
|  | USA | NR | Children and adults (aged 3–35 years) with TSC and epilepsy – ES without history of IS | 4 | Number of ASMs used for ES, range = 1–3 |
| Husain 2000 [79] | USA | NR | Children with TSC and epilepsy^†^ – ≥50% reduction in seizures | 10 | Mean number of ASMs = 5.4  Mean duration of adrenocorticotropin and prednisone treatment = 11.8 weeks |
|  | USA | NR | Children with TSC and epilepsy^†^ – <50% reduction in seizures | 7 | Mean number of ASMs = 6.1  Mean duration of adrenocorticotropin and prednisone treatment = 10.7 weeks |
| Lennert 2013 [80] | USA | NR | Children and adults with TSC and epilepsy (aged 0–50 years) | 95 | *Outcomes in first few years following diagnosis:*  Ketogenic diet = 5.3%  CBZ = 38.9%  CLB = 22.1%  FBM = 27.4%  GPN = 20.0%  LTG = 37.9%  LEV = 46.3%  OXC = 37.9%  PHT = 35.8%  VPA = 54.7%  TPM = 44.2%  VGB = 28.4%  Epilepsy surgery = 30.5%  Resective surgery = 8.4%  VNS = 5.3%  Indwelling electrodes with resection = 6.3%  Indwelling electrodes without resection = 3.2%  Partial CC = 4.2%  Complete CC = 1.1%  Laser treatment = 2.1% |
| Pashos 2012 [11] | USA | NR | Patients with TSC^†^ (46% had epilepsy) | 380 | TSC-related surgery = 52.0% |
|  | USA | NR | Patients with TSC-related surgery^†^ | 198 | Brain surgery = 33.0% |
| Patel 2021 [101] | USA | NR | Patients with TSC and epilepsy^†^ – on CLB with >50% seizure reduction | 13 | Average number of concomitant medications (not CLB) = 1.9 |
|  | USA | NR | Patients with TSC and epilepsy^†^ – on CLB without >50% seizure reduction | 10 | Average number of concomitant medications (not CLB) = 2.5 |
|  | USA | NR | Patients with TSC and epilepsy^†^ – who left the trial for lack of efficacy | 15 | Concomitant CLB = 60% |
|  | USA | NR | Patients with TSC and epilepsy^†^ – who left the trial for lack of efficacy on CLB | 9 | Mean number of concomitant medications (not CLB) = 2 |
| Peters 2018 [102] | USA | NR | Children (aged <3 years) with TSC and epilepsy | 25 | Median number of ASMs taken at time of surgery = 3 |
| Rentz 2015b [82] | USA | NR | Children (aged ≤18 years) with TSC (mean age 6.9 years, 78% had epilepsy) | 176 | Epilepsy surgery = 15.9%  SEGA surgery = 7.4%  ≥2 TSC medications = 64.0%  ASMs = 77.0%  Sleep medication = 28.0% |
|  | USA | NR | Adults with TSC (mean age 42.3 years; 52% had epilepsy) | 99 | Epilepsy surgery = 12.1%  SEGA surgery = 10.1%  ≥2 TSC medications = 75.0%  ASMs = 36.0%  Sleep medication = 23.0%  Antidepressants, antipsychotics or anxiolytics = 41.0% |
| Schoenberger 2019 [53] | USA | NR | Children with TSC (78% had epilepsy by 36 months) – aged 6 months | 111 | >1 ASM = 14.4%  >2 ASMs = 6.3%  Physical therapy = 14.4%  Occupational therapy = 6.3%  Speech/language therapy = 0.9%  Early intervention therapy = 6.3%  Applied behavioural analysis = 0% |
|  | USA | NR | Children with TSC (78% had epilepsy by 36 months) – aged 9 months | 143 | >1 ASM = 26.6%  >2 ASMs = 11.2%  Physical therapy = 17.5%  Occupational therapy = 16.1%  Speech/language therapy = 4.2%  Early intervention therapy = 21.7%  Applied behavioural analysis = 0% |
|  | USA | NR | Children with TSC (78% had epilepsy by 36 months) – aged 12 months | 154 | >1 ASM = 39.6%  >2 ASMs = 21.4%  Physical therapy = 23.4%  Occupational therapy = 22.1%  Speech/language therapy = 10.4%  Early intervention therapy = 24.0%  Applied behavioural analysis = 1.3% |
|  | USA | NR | Children with TSC (78% had epilepsy by 36 months) – aged 18 months | 144 | >1 ASM = 50.0%  >2 ASMs = 29.9%  Physical therapy = 38.2%  Occupational therapy = 41.7%  Speech/language therapy = 30.6%  Early intervention therapy = 36.1%  Applied behavioural analysis = 1.4% |
|  | USA | NR | Children with TSC (78% had epilepsy by 36 months) – aged 24 months | 139 | >1 ASM = 54.0%  >2 ASMs = 23.0%  Physical therapy = 24.5%  Occupational therapy = 23.7%  Speech/language therapy = 34.5%  Early intervention therapy = 18.7%  Applied behavioural analysis = 3.6% |
|  | USA | NR | Children with TSC (78% had epilepsy by 36 months) – aged 36 months | 137 | >1 ASM = 56.2%  >2 ASMs = 30.0%  Physical therapy = 16.8%  Occupational therapy = 25.5%  Speech/language therapy = 38.7%  Early intervention therapy = 8.8%  Applied behavioural analysis = 7.3% |
| Skalicky 2015 [83] | USA | NR | Adults (aged >18 years) with TSC (age range 19–60 years; 48% had epilepsy) | 69 | SEGA surgery = 19.0%  Mean number of SEGA surgeries = 1.2  Epilepsy surgery = 15.0%  Mean number of epilepsy surgeries = 1.6  Surgery on SENs = 10.0%  Mean number of surgeries on SENs = 2.1  Shunt placement surgery = 13.0%  Mean number of shunt placement surgeries = 1.6  Antidepressants, anti-anxiety or antipsychotics = 51.0%  ASMs = 45.0% |
|  | USA | NR | Children (aged ≤18 years) with TSC (age range 1–18 years; 89% had epilepsy) | 47 | SEGA surgery = 28.0%  Mean number of SEGA surgeries = 1.2  Epilepsy surgery = 30.0%  Mean number of epilepsy surgeries = 1.5  Shunt placement surgery = 9.0%  Mean number of shunt placement surgeries = 1  ASMs = 77.0%  Sleep medication = 23.0% |
| van Eeghen 2012 [13] | USA | NR | Children and adults with TSC (aged 0.5–20 years; 94% had epilepsy) | 62 | VPA = 22.6%  LTG = 19.4%  LEV = 14.5%  CBZ = 12.9%  VGB = 8.1%  GPN = 6.5%  TPM = 4.8% |
|  | USA | NR | Children and adults with TSC (aged 0.5–20 years; 98% had epilepsy) | 62 | Not taking ASMs = 21.0%  Taking ASMs = 11.3%  Stopping ASMs = 8.1%  Decreasing ASMs = 9.7%  Increasing ASMs = 19.4%  LTG = 37.1%  LEV = 30.6%  VPA = 12.9%  CBZ = 9.6%  GPN = 6.5%  TPM = 4.8%  VGB = 4.8% |
| Chung 2017 [20] | Australia | NR | Children^†^ with TSC and epilepsy | 61 | VPA = 77.0%  VGB = 75.4%  CBZ = 50.8%  LEV = 45.9%  TPM = 34.4%  LTG = 27.9%  CLB = 24.6%  PB = 18.0%  ZNS = 9.8%  Other ASMs = 27.9%  Steroids = 8.2%  Ketogenic diet = 18.0%  Epilepsy surgery = 9.8%  mTOR inhibitors = 19.7% |
| Deverell 2022 [87] | Australia | 2015–2016 | Children with TSC (median [range] 7 [1–12] years for 15 children with rare diseases; 10 with TSC) | 10 | Number of visits over 6 months:  Specialists = 21  GPs = 5  Allied health professionals = 19 |
| Samueli 2015 [103] | Austria | NR | Children (aged ≤19 years) with TSC and epilepsy | 12 | EVE = 100% |
| Samueli 2016 [104] | Austria | NR | Children (aged ≤18 years) with TSC and refractory epilepsy | 15 | LEV = 33.3%  VGB = 26.7%  OXC = 26.7%  TPM = 20.0%  RFM = 20.0%  PHT = 6.7%  PGB = 6.7%  ZNS = 6.7%  VNS = 26.7%  Ketogenic diet = 6.7%  Incomplete SEGA surgery = 6.7%  Epilepsy surgery = 6.7% |
| Marques 2019 [91] | Belgium | NR | Children and adults with TSC and focal epilepsy (aged 0–71 years) | 64 | mTOR inhibitors = 1.6%  Epilepsy surgery = 12.5%  GABAergics = 82.8%  ACTH = 1.6%  Ketogenic diet = 3.1%  Fructose derivatives = 7.8%  VNS = 10.9%  Other treatments = 56.3% |
| Whitney 2022 [105] | Canada | NR | Paediatric epileptologists (39%), paediatric (42%) and adult (11%) neurologists and other healthcare professionals (9%) | 57 | *Healthcare professional outcomes:*  Reported seeing 0–10 patients with TSC = 69.0%  Reported seeing 10–30 patients with TSC = 13.0%  Reported seeing 30–50 patients with TSC = 2.0%  Reported seeing 50–100 patients with TSC = 7.0%  Reported seeing >100 patients with TSC = 9.0%  Access to paediatric-specific TSC unit = 25.0%  Access to routine genetic testing in TSC = 62.0%  Considered using mTOR inhibitors for intractable seizures = 26.0%  Used EEG screening in newly diagnosed patients = 70.8%  Used ASMs in the absence of seizures = 57.0%  Used VGB as first-line treatment for prevention = 84.6%  Prescribed ASM for prevention for <6 months = 5.0%  Prescribed ASM for prevention for 6–12 months = 50.0%  Prescribed ASM for prevention for >12 months = 45.0% |
| Wilbur 2017 [15] | Canada | NR | Children and adults with TSC (aged 0.2–23.2 years) – with epilepsy | 74 | Epilepsy surgery = 26% |
|  | Canada | NR | Children with TSC and epilepsy (aged 1.1–15.6 years) – had epilepsy surgery | 19 | FR as first surgery = 63.2%  PR as first surgery = 10.5%  TR as first surgery = 5.3%  MR as first surgery = 10.5%  TCC as first surgery = 10.5%  Second surgery = 21.1%  FR as second surgery = 10.5%  MR as second surgery = 5.3%  TCC as second surgery = 5.3% |
| Liang 2017 [66] | China | NR | Children and adults with TSC and epilepsy (aged 5–28 years) – who had surgery | 51 | Mean number of ASMs used after 5 years’ follow-up = 2.17 |
|  | China | NR | Patients with TSC and epilepsy^†^ – without surgery | 15 | Mean number of ASMs used after 5 years’ follow-up ASMs used = 2.87 |
| Zhao 2022 [106] | China | NR | Children with TSC and epilepsy (aged 2–156 months at seizure onset) | 103 | Sirolimus = 87.4%  VPA = 68.9%  VGB = 63.1%  OXC = 46.6%  LEV = 40.8%  LTG = 26.2%  TPM = 24.3%  NZP = 18.5%  CBZ = 6.8%  PB = 5.8%  CZP = 4.9%  ACTH = 2.9%  LCM = 1.9%  Prednisolone = 1.9%  Elazide = 1.0%  Aniracetam = 1.0% |
| Baumgartner 2021 [107] | Europe | 2018–2019 | Children with TSC and epilepsy^†^ |  | Proportion of specialist centres in Europe using the following treatments in TSC:  VGB for infantile spasms as first-line = 96.0%  VGB as first-line for other seizure types = 30.0%  ACTH as second-line therapy for IS = 48.0%  CLB as second-line therapy for other seizures = 22.0%  VPA as third-line therapy for IS = 26.0%  LEV as third-line therapy for other seizures = 26.0%  MDZ as first-line SE treatment = 52.0%  BZD as first-line SE treatment = 100%  PHT as second-line SE treatment = 30.0%  LEV as second-line SE treatment = 30.0%  PHT as third-line SE treatment = 26.0%  Treatment monitored by therapeutic drug monitoring = 61.0%  EVE as third-line treatment = 65.0%  EVE as fourth-line treatment = 35.0%  EVE as fifth-line treatment = 35.0% |
| Fagnani 2022 [90] | France | 2018 | Children and adults (aged 9–≥70 years) with TSC (67% had epilepsy) | 2,936 | Hospitalised at least once = 41.0%  Visited a GP at least once = 66.0%  GP visit and/or hospital specialist = 61.0% |
|  | France | 2018 | Children and adults (aged 9–≥70 years) with TSC and epilepsy | 1,989 | Hospitalised = 46.0%  Visited a hospital specialist = 65.0%  Mean number of nurse visits = 11 (SD 51.6)  Mean number of physiotherapist visits = 4.9 (SD 18.6)  Dispensed ≥1 ASM = 74.0%  Dispensed ≥1 ASM including CBZ = 25.0%  Dispensed ≥3 ASMs including CBZ = 23.0%  Dispensed ≥1 ASM including VPA= 21.0%  Dispensed ≥3 ASMs including VPA = 19.0%  Dispensed ≥1 ASM including LTG = 18.0%  Dispensed ≥3 ASMs including LTG = 16.0%  Dispensed ≥1 ASM including VGB = 15.0%  Dispensed ≥3 ASMs including VGB = 14.0%  Dispensed ≥1 ASM including CLB = 15.0%  Dispensed ≥3 ASMs including CLB = 11.0%  Dispensed ≥1 ASM including LEV = 13.0%  Dispensed ≥3 ASMs including LEV = 11.0%  Dispensed EVE = 9.0% |
|  | France | 2018 | Children and adults (aged 9–≥70 years) with TSC and no epilepsy | 947 | Hospitalised = 32.0%  Visited a hospital specialist = 51.0%  Mean number of nurse visits = 4.4 (SD 23.9)  Mean number of physiotherapist visits = 3.5 (SD 13.7)  Dispensed ≥1 ASM = 94.0%  Dispensed EVE = 4.0% |
| Marques 2019 [91] | France | NR | Children and adults with TSC and focal epilepsy (aged 0–71 years) | 165 | mTOR inhibitors = 10.9%  Epilepsy surgery = 9.7%  GABAergics = 84.2%  ACTH = 1.8%  Ketogenic diet = 7.3%  Fructose derivatives = 6.7%  VNS = 9.1%  Other treatments = 74.5% |
| Nabbout 2021 [30] | France | 1940–1950 | Children and adults with TSC and epilepsy^†^ – focal seizures | 5 | VGB = 20.0%  ACTH = 0%  Ketogenic diet = 0%  Fructose derivatives = 0%  VNS = 0%  mTOR inhibitors = 0%  Surgery = 0%  Other types = 100% |
|  | France | >1950–1960 | Children and adults with TSC and epilepsy^†^ – focal seizures | 4 | VGB = 33.3%  ACTH = 0%  Ketogenic diet = 0%  Fructose derivatives = 0%  VNS = 0%  mTOR inhibitors = 0%  Surgery = 0%  Other treatments = 66.7% |
|  | France | >1950–1960 | Children and adults with TSC and epilepsy^†^ – focal seizures | 8 | VGB = 50.0%  ACTH = 0%  Ketogenic diet = 0%  Fructose derivatives = 0%  VNS = 0%  mTOR inhibitors = 0%  Surgery = 0%  Other treatments = 62.5% |
|  | France | >1960–1970 | Children and adults with TSC and epilepsy^†^ – focal seizures | 17 | VGB = 37.5%  ACTH = 18.8%  Ketogenic diet = 6.3%  Fructose derivatives = 0%  VNS = 0%  mTOR inhibitors = 6.3%  Surgery = 0%  Other treatments = 81.3% |
|  | France | >1960–1970 | Children and adults with TSC and epilepsy^†^ – focal seizures | 27 | VGB = 42.3%  ACTH = 0%  Ketogenic diet = 0%  Fructose derivatives = 15.4%  VNS = 0%  mTOR inhibitors = 3.8%  Surgery = 0%  Other treatments = 88.5% |
|  | France | >1970–1980 | Children and adults with TSC and epilepsy^†^ – focal seizures | 29 | VGB = 40.7%  ACTH = 22.2%  Ketogenic diet = 0%  Fructose derivatives = 0%  VNS = 0%  mTOR inhibitors = 3.7%  Surgery = 0%  Other treatments = 63.0% |
|  | France | >1970–1980 | Children and adults with TSC and epilepsy^†^ – focal seizures | 57 | VGB = 50.9%  ACTH = 1.9%  Ketogenic diet = 0%  Fructose derivatives = 7.5%  VNS = 3.8%  mTOR inhibitors = 17.0%  Surgery = 3.8%  Other treatments = 73.6% |
|  | France | >1980–1990 | Children and adults with TSC and epilepsy^†^ – focal seizures | 70 | VGB = 68.2%  ACTH = 30.3%  Ketogenic diet = 1.5%  Fructose derivatives = 0%  VNS = 1.5%  mTOR inhibitors = 6.1%  Surgery = 1.5%  Other treatments = 59.1% |
|  | France | >1980–1990 | Children and adults with TSC and epilepsy^†^ – focal seizures | 135 | VGB = 61.8%  ACTH = 3.1%  Ketogenic diet = 1.5%  Fructose derivatives = 6.1%  VNS = 6.1%  mTOR inhibitors = 13.0%  Surgery = 6.1%  Other treatments = 73.3% |
|  | France | >1990–1995 | Children and adults with TSC and epilepsy^†^ – focal seizures | 63 | VGB = 62.9%  ACTH = 33.9%  Ketogenic diet = 0%  Fructose derivatives = 3.2%  VNS = 3.2%  mTOR inhibitors = 1.6%  Surgery= 0%  Other treatments = 56.5% |
|  | France | >1990–1995 | Children and adults with TSC and epilepsy^†^ – focal seizures | 93 | VGB = 66.7%  ACTH = 2.2%  Ketogenic diet = 0%  Fructose derivatives = 7.8%  VNS = 11.1%  mTOR inhibitors = 11.1%  Surgery= 6.7%  Other treatments = 67.8% |
|  | France | >1995–2000 | Children and adults with TSC and epilepsy^†^ – infantile spasms | 80 | VGB = 83.3%  ACTH = 16.7%  Ketogenic diet = 2.6%  Fructose derivatives = 2.6%  VNS = 1.3%  mTOR inhibitors = 5.1%  Surgery = 0%  Other treatments = 46.2% |
|  | France | >1995–2000 | Children and adults with TSC and epilepsy^†^ – infantile spasms | 168 | VGB = 77.1%  ACTH = 1.8%  Ketogenic diet = 3.6%  Fructose derivatives = 4.2%  VNS = 4.8%  mTOR inhibitors = 14.5%  Surgery = 6.0%  Other treatments = 69.9% |
|  | France | >2000–2005 | Children and adults with TSC and epilepsy^†^ – infantile spasms | 122 | VGB = 86.2%  ACTH = 13.8%  Ketogenic diet = 8.6%  Fructose derivatives = 2.6%  VNS = 6.0%  mTOR inhibitors = 3.4%  Surgery = 7.8%  Other treatments = 33.6% |
|  | France | >2000–2005 | Children and adults with TSC and epilepsy^†^ – infantile spasms | 246 | VGB = 69.1%  ACTH = 3.7%  Ketogenic diet = 10.7%  Fructose derivatives = 6.2%  VNS = 7.8%  mTOR inhibitors = 16.5%  Surgery = 10.3%  Other treatments = 73.7% |
|  | France | >2005–2010 | Children and adults with TSC and epilepsy^†^ – infantile spasms | 178 | VGB = 88.1%  ACTH = 14.2%  Ketogenic diet = 4.0%  Fructose derivatives = 2.8%  VNS = 4.0%  mTOR inhibitors = 9.1%  Surgery = 8.0%  Other treatments = 47.2% |
|  | France | >2005–2010 | Children and adults with TSC and epilepsy^†^ – infantile spasms | 361 | VGB = 73.3%  ACTH = 3.6%  Ketogenic diet = 4.4%  Fructose derivatives = 5.3%  VNS = 3.6%  mTOR inhibitors = 10.4%  Surgery = 9.7%  Other treatments = 67.8% |
|  | France | >2010–2015 | Children and adults with TSC and epilepsy^†^ – infantile spasms | 172 | VGB = 91.2%  ACTH = 10.0%  Ketogenic diet = 7.6%  Fructose derivatives = 2.4%  VNS = 0%  mTOR inhibitors = 17.1%  Surgery = 7.1%  Other treatments = 45.9% |
|  | France | >2010–2015 | Children and adults with TSC and epilepsy^†^ – infantile spasms | 243 | VGB = 76.9%  ACTH = 3.8%  Ketogenic diet = 11.8%  Fructose derivatives = 8.0%  VNS = 1.3%  mTOR inhibitors = 18.1%  Surgery = 8.8%  Other treatments = 69.7% |
| Baumgartner 2021 [107] | Germany | NR | Children and adults with TSC and epilepsy^†^ – infantile spasms | NR | VGB as first-line = 96.0%  ACTH as second-line = 48.0%  VPA as third-line = 26.0% |
|  | Germany | NR | Children and adults with TSC and epilepsy^†^ – other seizures | NR | VGB as first-line = 30.0%  CBZ as second-line = 22.0%  LEV as third-line = 26.0% |
|  | Germany | NR | Children and adults with TSC and epilepsy^†^ – status epilepticus | NR | MDZ as first-line = 52.0%  BZD as first-line = 100%  PHT as second-line = 30.0%  LEV as second-line = 30.0%  PHT as third-line = 26.0% |
|  | Germany | NR | Patients with TSC and epilepsy^†^ – 23 epilepsy centres | NR | mTOR inhibitors available = 91.0%  Provide epilepsy surgery programme = 87.0%  Conducting drug monitoring in majority of cases = 61.0%  Conducting drug monitoring in select cases = 39.0%  Requiring serum concentrations to adjust doses of drug = 0% (not requested) |
| Ebrahimi-Fakhari 2019 [108] | Germany | 2015–2017 | Children (aged ≤18 years) with TSC and epilepsy | 17 | VGB = 82.4%  LEV = 58.8%  OXC = 29.4%  VPA = 23.5%  EVE = 23.5%  LTG = 17.6%  PB = 17.6%  Ketogenic diet = 17.6%  TPM = 11.8%  CLB = 11.8%  ≥2 ASMs = 76.5% |
| Hamer 2016 [109] | Germany | NR | Adults with TSC and epilepsy (mean age 36 years) | 194 | Epilepsy surgery = 10.0%  1 ASM = 20.0%  2 ASMs = 45.0%  ≥3 ASMs = 32.0% |
| Marques 2019 [91] | Germany | NR | Children and adults with TSC and focal epilepsy (aged 0–71 years) | 103 | mTOR inhibitors = 20.4%  Epilepsy surgery = 10.7%  GABAergics = 83.5%  ACTH = 1.0%  Ketogenic diet = 11.7%  Fructose derivatives = 1.9%  VNS = 1.0%  Other treatments = 58.3% |
| Strzelczyk 2021a [110] | Germany | 2019 | Children and adults with TSC and epilepsy (mean age 19.0 years) | 268 | Mean (median, range) number of ASMs used = 1.8 (2, 1–4)  LTG = 34.7%  VPA = 32.8%  OXC = 28.7%  VGB = 19.0%  LEV = 17.9%  EVE = 32.5%  LCM = 8.6%  TPM = 4.9%  ZNS= 3.7%  CBZ = 3.7%  CLB = 3.7%  BRV = 3.7%  Sulthiame = 3.4%  PHT = 2.6%  ESM = 2.6%  PER = 2.6%  PHT = 2.2%  RFM = 2.2%  PRM = 1.5%  Other ASM = 4.5%  Monotherapy OXC = 11.6%  Polytherapy VPA and LTG = 7.8%  Monotherapy LTG = 7.1%  ASM monotherapy = 37.7%  ASM polytherapy = 62.3% |
|  | Germany | 2019 | Children and adults with TSC and epilepsy (mean age 19.0 years for total population) – on ASM monotherapy | 101 | OXC monotherapy = 30.7%  LTG monotherapy = 18.8%  VGB monotherapy = 15.8%  VPA monotherapy = 11.9%  LEV monotherapy = 9.9%  Patients using EVE = 22.7% |
|  | Germany | 2019 | Children and adults with TSC and epilepsy (mean age 19.0 years for total population) – on ASM polytherapy | 167 | 2 ASMs = 118  3 ASMs = 42  4 ASMs = 7  VPA = 45.5%  LTG = 44.3%  OXC = 27.4%  LEV = 22.8%  VGB = 21.0%  LCM = 12.0%  TPM = 7.8%  ZNS = 6.0%  CLB = 6.0%  Sulthiame = 4.8%  CBZ = 4.8%  PER = 4.2%  ESM = 3.6%  RFM = 3.6%  PHT = 3.0%  VPA and LTG = 12.6%  VPA and OXC = 5.4%  OXC and LEV = 5.4%  VGB and LTG = 3.6%  LEV and LTG = 3.6%  VGB and VPA = 3.0%  OXC and LTG = 3.0%  EVE = 38.3% |
|  | Germany | 2019 | Adults (aged ≥20 years) with TSC and epilepsy | 128 | EVE = 38.2%  VGB = 4.7% |
|  | Germany | 2019 | Children (aged ≤19 years) with TSC and epilepsy | 140 | EVE = 27.1%  VGB = 32.1% |
|  | Germany | 2019 | Children (aged 0–4 years) with TSC and epilepsy | 33 | Total ASMs = 62  Average ASMs per patient = 1.9  VGB = 58.0%  VPA = 39.0%  LTG = 24.0%  LEV = 21.0%  OXC = 18.0% |
|  | Germany | 2019 | Children (aged 5–9) with TSC and epilepsy | 44 | Total ASMs = 69  Average ASMs per patient = 1.6  VGB = 36.0%  OXC = 42.0%  LTG = 27.0%  VPA = 23.0%  LEV = 16.0% |
|  | Germany | 2019 | Children (aged 10–14) with TSC and epilepsy | 38 | Total ASMs = 73  Average ASMs per patient = 1.9  VPA = 32.0%  OXC = 32.0%  LTG = 29.0%  VGB = 24.0%  LEV = 18.0% |
|  | Germany | 2019 | Children and adults (aged 15–19) with TSC and epilepsy | 45 | Total ASMs = 73  Average ASMs per patient = 1.6  OXC = 40.0%  LTG = 33.0%  VPA = 22.0%  LEV = 15.0%  LCM = 13.0% |
|  | Germany | 2019 | Adults (aged 20–29) with TSC and epilepsy | 55 | Total ASMs = 114  Average ASMs per patient = 2.1  VPA = 44.0%  LTG = 40.0%  OXC = 29.0%  LEV = 16.0%  VGB = 9.0% |
|  | Germany | 2019 | Adults (aged 30–39) with TSC and epilepsy | 33 | Total ASMs = 62  Average ASMs per patient = 1.9  LTG = 45.0%  VPA = 45.0%  OXC = 18.0%  LEV = 18.0%  LCM = 12.0% |
|  | Germany | 2019 | Adults (aged ≥40) with TSC and epilepsy | 20 | Total ASMs = 38  Average ASMs per patient = 1.9  LTG = 50.0%  OXC = 25.0%  LEV = 25.0%  VPA = 20.0%  ZNS = 15.0% |
|  | Germany | 2019 | Children and adults with TSC and epilepsy (mean age 19.0 years) – high seizure frequency | 133 | Total ASMs = 294  Average ASMs per patient = 2.2  VPA = 44.0%  LTG = 39.0%  OXC = 26.0%  LEV = 19.0%  VGB = 14.0% |
|  | Germany | 2019 | Children and adults with TSC and epilepsy (mean age 19.0 years) – low seizure frequency | 21 | Total ASMs = 33  Average ASMs per patient = 1.6  OXC = 39.0%  LTG = 29.0%  LEV = 24.0%  LCM = 19.0%  VGB = 19.0% |
|  | Germany | 2019 | Children and adults with TSC and epilepsy (mean age 19.0 years) – seizure-free for >1 year | 114 | Total ASMs = 164  Average ASMs per patient = 1.4  LTG = 31.0%  OXC = 30.0%  VPA = 23.0%  VGB = 19.0%  LEV = 15.0% |
| Strzelczyk 2021b [19] | Germany | 2007–2016 | Children and adults with TSC  (aged 1–86 years; 36% had epilepsy) | 100 | Mean (SD) number of medications prescribed PPY over 10 years = 6.3 (5.1) (median = 5)  Mean (SD) number of medications prescribed over entire observable period = 19.2 (14.4) (median = 15)  Ibuprofen = 24.0%  Levothyroxine = 11.0%  Metamizole = 10.0% |
|  | Germany | 2007–2016 | Children and adults with TSC and epilepsy (aged 2–86 years) | 29 | Mean (SD) number of ASMs prescribed PPY = 2.0 (1.1)  Mean number of medications prescribed PPY over 10 years = 7.7 (5.6) (median = 6)  Mean number of medications prescribed over entire observable period = 21.6 (16.9) (median = 17)  1 ASM = 32.0%  2 ASMs = 18.0%  3 ASMs = 10.0%  Rescue medication on ≥1 occasion = 30.1%  VPA = 23.0%  LTG = 19.0%  CBZ = 16.0%  VGB = 13.0%  LEV = 12.0% |
| Willems 2021a [111] | Germany | 2019 | Children and adults (aged <18 and ≥18 years) with TSC (84% had epilepsy) | 134 | At least 1 dose of EVE during life = 36.7%  Using EVE at study entry = 31.5%  Mean (SD) EVE dose in adults = 6.8 (3.5) mg/day  Mean (SD) EVE dose in children = 5.1 (2.9) mg/day  Mean (SD) EVE dose = 6.1 (3.4) mg/day  Systemic sirolimus = 1.4%  EVE for DRE = 53.0%  EVE for angiomyolipoma = 52.2%  EVE for SEGA = 38.8%  EVE for CRM = 12.7%  EVE for dermal TSC manifestations = 9.0%  EVE for cerebral tubers = 3.7%  EVE for single TSC indication = 43.3%  EVE for multiple TSC indications = 56.0% |
| Kingswood 2017 [112] | International | NR | Children and adults with TSC and epilepsy (aged 0–71 years) – TSC-associated epilepsy | 1,144 | GABAergics = 65.1%  mTOR inhibitors = 7.0%  Surgery = 7.0%  Ketogenic diet = 4.3%  VNS = 3.9%  Fructose derivatives = 2.9%  ACTH = 2.7% |
| Nabbout 2019 [27] | International | NR | Children and adults with TSC and epilepsy (aged 0–71 years) – TSC-associated FS | 1,250 | GABAergics = 65.5%  mTOR inhibitors = 7.7%  Epilepsy surgery = 6.9%  Ketogenic diet = 4.7%  VNS = 3.8%  Fructose derivatives = 3.5%  ACTH = 2.9% |
|  | International | NR | Children and adults with TSC and epilepsy (aged 0–71 years) – early onset FS | 984 | GABAergics = 70.5%  ACTH = 3.3% |
|  | International | NR | Children and adults with TSC and epilepsy (aged 0–71 years) – IS | 720 | GABAergics = 78.7%  ACTH = 17.5%  mTOR inhibitors = 5.5%  Epilepsy surgery = 4.2%  Ketogenic diet = 3.9%  VNS = 2.2%  Fructose derivatives = 1.3% |
|  | International | NA | Children and adults with TSC and epilepsy (aged 0–71 years) – early onset IS | 684 | GABAergics = 79.5%  ACTH = 18.3% |
| Song 2018 [25] | International | NR | Patients with TSC and epilepsy^†^ – receiving treatment | 1,025 | ASMs = 99.5%  mTOR inhibitors = 1.0%  Diet = 7.9%  Epilepsy surgery = 25.3% |
|  | International | NR | Patients with TSC and epilepsy^†^ –  treated with an ASM | 1,020 | 1 ASM = 17.9%  2 ASMs = 17.5%  3 ASMs = 15.9%  4 ASMs = 13.9%  ≥5 ASMs = 34.7%  Surgery following ASMs = 22.5% |
|  | International | NR | Patients with TSC and epilepsy^†^ – had surgery after ASMs | 229 | CC = 10.8%  Hemispherectomy = 2.3%  Resection = 64.9%  VNS-related procedure = 44.4%  1 surgical procedure = 64.9%  2 surgical procedures = 24.7%  ≥3 surgical procedures = 10.4% |
| Marques 2019 [91] | International | NR | Children and adults with TSC and focal epilepsy (aged 0–71 years) | 1,237 | *Baseline (n=1,237):*  GABAergics = 66.2%  ACTH = 2.8%  Ketogenic diet = 4.8%  Fructose derivatives = 4.0%  VNS = 3.9%  mTOR inhibitors = 8.3%  Epilepsy surgery = 6.8%  Other treatments = 66.5%  *Year Five (n=28):*  GABAergics = 46.4%  ACTH = 0%  Ketogenic diet = 3.6%  Fructose derivatives = 0%  VNS = 3.6%  mTOR inhibitors = 14.3%  Epilepsy surgery = 3.6%  Other treatments = 78.6%  *Data for years 1, 2, 3 and 4 are available in the data extraction spreadsheet* |
|  | International | NR | Children and adults with TSC and focal epilepsy (aged 0–71 years) | 1,261 | *Received treatment for focal seizures:*  Baseline = 98.1%  Year 1 = 97.6%  Year 2 = 97.4%  Year 3 = 97.4%  Year 4 = 97.9%  Year 5 = 96.6% |
| Slowinska 2021 [113] | International | NR | Children with TSC and epilepsy^†^ | 60 | Performed at least 1 EEG before seizure onset = 95.0%  Performed repeated EEGs before seizure onset = 73.7%  Repeated EEG once seizures started = 19.3%  Regular EEG every 4–12 weeks = 61.4%  Use of video EEG = 68.8%  Begin ASM treatment after clinical seizures = 33.3%  Introduction of ASM after epileptiform discharges on EEG but before clinical seizures = 51.7%  *First choice treatment of focal seizures in children aged <2 years with TSC:*  VGB = 61.7%  CBZ = 15%  LEV = 15.0%  OXC = 10.0%  CLB = 1.7%  VPA = 1.7%  CZP = 1.7%  Sulthiame = 1.7%  ZNS = 1.7%  TPM = 0%  LCM = 0%  LTG = 0%  PB = 0%  EVE = 0%  *First choice treatment of focal seizures in children aged >2 years with TSC:*  CBZ = 42.6%  LEV = 29.5%  VGB = 21.3%  OXC = 24.6%  CLB = 0%  VPA = 3.3%  CZP = 0%  Sulthiame = 0%  ZNS = 1.6%  TPM = 9.8%  LCM = 6.6%  LTG = 0%  PB = 0%  EVE = 0%  Other ASMs = 1.6%  *First choice treatment of general seizures in children aged <2 years with TSC:*  VGB = 56.7%  CBZ = 3.3%  LEV = 11.7%  OXC = 0%  CLB = 5.0%  VPA = 26.7%  CZP = 0%  Sulthiame = 0%  ZNS = 1.7%  TPM = 3.3%  LCM = 0%  LTG = 1.6%  PB = 1.6%  EVE = 1.6%  Other ASMs = 0% |
| Thiele 2021 [75] | International | NR | Children and adults  (aged 1.2–55.8 years) with TSC and epilepsy – receiving placebo | 76 | Median (range) number of previous ASMs = 4 (0–15)  Median (range) number of current ASMs = 3 (1–5)  1 current ASM = 11.0%  2 current ASMs = 36.0%  ≥3 current ASMs = 54.0%  Median (range) number of previous and current ASMs = 7 (2–18)  *Currently using ASM, %:*  VPA = 46.0%  VGB = 22.0%  LEV = 32.0%  CLB = 33.0%  *Previously used ASM, %:*  VPA = 30.0%  Previous VGB = 55.0%  Previous LEV = 47.0%  Previous CLB = 29.0%  Previous EVE = 9.0% |
|  | International | NR | Children and adults with TSC and epilepsy (aged 1.1–56.8 years) – receiving CBD (25 mg/kg/day) | 75 | Median (range) number of previous ASMs = 4 (0–13)  Median (range) number of current ASMs = 3 (0–4)  1 current ASM = 12.0%  2 current ASMs = 27.0%  ≥3 current ASMs = 60.0%  Median (range) number of previous and current ASMs = 7 (1–15)  Current VPA = 39.0%  Current VGB = 37.0%  Current LEV = 25.0%  Current CLB = 23.0%  Previous VPA = 37.0%  Previous VGB = 35.0%  Previous LEV = 52.0%  Previous CLB = 32.0%  Previous EVE = 9.0% |
|  | International | NR | Children and adults with TSC and epilepsy (aged 1.8–34.9 years) – receiving CBD (50 mg/kg/day) | 73 | Median (range) number of previous ASMs = 4 (0–13)  Median (range) number of current ASMs = 3 (1–5)  1 current ASM = 10.0%  2 current ASMs = 33.0%  ≥3 current ASMs = 58.0%  Median (range) number of previous and current ASMs = 7 (1–15)  Current VPA = 49.0%  Current VGB = 40.0%  Current LEV = 30.0%  Current CLB = 26.0%  Previous VPA = 34.0%  Previous VGB = 40.0%  Previous LEV = 45.0%  Previous CLB = 22.0%  Previous EVE = 10.0% |
| Shlomovitz 2021 [114] | Israel | NR | Children and adults (aged <18 and ≥18 years) with TSC and epilepsy – controlled epilepsy | 42 | Mean number of ASMs used for treatment = 1.2 |
|  | Israel | NR | Children and adults (aged <18 and ≥18 years) with TSC and epilepsy – refractory epilepsy | 18 | Mean number of ASMs used for treatment = 2.5 |
|  | Israel | NR | Patients with TSC and SEGA | 29 | SEGA surgery = 13.8% |
| Marques 2019 [91] | Italy | NR | Children and adults with TSC and focal epilepsy (aged 0–71 years) | 70 | mTOR inhibitors = 0%  Epilepsy surgery = 4.3%  GABAergics = 52.9%  ACTH = 2.9%  Ketogenic diet = 0%  Fructose derivatives = 0%  VNS = 0%  Other treatments = 91.4% |
| Mingarelli 2018 [115] | Italy | NR | Infants with prenatal suspicion of TSC  (67% developed epilepsy)^†^ | 6 | VGB = 33.0% |
| Vignoli 2013 [14] | Italy | NR | Children and adults with TSC  (aged 1–74 years, mean 31.6 years) – without seizures | 37 | SEGA surgery = 2.7% |
|  | Italy | NR | Children and adults with TSC with epilepsy (overall range 1–74 years) – seizure-free | 57 | 0 ASMs = 12.3%  1 ASM = 56.1%  ASM polytherapy = 31.6%  Epilepsy surgery = 3.5%  CBZ monotherapy = 21.1%  VPA monotherapy = 12.3%  PB monotherapy = 7.0%  PHT monotherapy = 3.5%  PRM monotherapy = 3.5%  GPN monotherapy = 1.8%  LEV monotherapy = 1.8%  OXC monotherapy = 1.8%  ZNS monotherapy = 1.8%  SEGA surgery = 3.5% |
|  | Italy | NR | Children and adults with TSC with epilepsy (overall range 1–74 years) –drug-resistant epilepsy | 59 | 0 ASMs = 0  1 ASM = 15.3%  ASM polytherapy = 84.7%  Epilepsy surgery = 6.8%  CBZ monotherapy = 5.1%  VPA monotherapy = 8.5%  OXC monotherapy = 1.7%  SEGA surgery = 8.5% |
| Vignoli 2021 [62] | Italy | NR | Adults (>18 years) with TSC and epilepsy | 183 | No treatment = 8.2%  Monotherapy = 32.8%  Polytherapy = 47%  EVE only = 2.7%  VNS = 2.2%  Epilepsy surgery = 6.0% |
|  | Italy | NR | Adults (>18 years) with TSC and epilepsy – seizure-free on monotherapy | 59 | CBZ = 55.2%  VPA= 20.7%  OXC = 10.3%  Other monotherapy = 13.8% |
|  | Italy | NR | Adults (>18 years) with TSC and epilepsy – refractory epilepsy on monotherapy | 121 | Polytherapy = 64.5%  CBZ = 37.1%  LTG = 11.4%  Other drugs = 11.4% |
| LoPresti 2020 [116] | Japan | NR | Patients with TSC and refractory epilepsy^†^ | NR | Mean (SD) number of ASMs prescribed for confirmed TSC = 3.0 (1.7)  Mean (SD) number of ASMs prescribed for suspected TSC = 2.4 (1.5) |
| Gumbelevičiene 2014 [40] | Lithuania | 1982–2012 | Children^†^ with TSC and epilepsy | 41 | VGB = 41.0% |
| Hulshof 2021 [117] | Netherlands | NR | Children^†^ with TSC and epilepsy | 41 | Mean (range) number of ASMs ever tried at 2 years of age = 3.5 (1–14)  Patients who received preventive treatment = 48.7% |
| Overwater 2015 [118] | Netherlands | 1988–2014 | Children with TSC and epilepsy (aged 0.9–18.0 years) | 71 | Median number of ASMs per child = 4  Median number of simultaneous treatments = 2  Ketogenic diet = 8.0%  Epilepsy surgery = 8.0%  VNS = 1.0%  VPA = 84.5%  VGB = 60.5%  LEV = 46.5%  CBZ = 40.8%  CLB = 40.8%  LTG = 32.4%  OXC = 22.5%  TPM = 18.3%  PB = 14.1%  PHT = 12.7%  Steroids = 11.3%  CZP = 11.3%  Acetazolamide = 4.2%  NZP = 4.2%  FBM = 4.2%  LCM = 2.8%  ESM = 2.8%  ZNS = 1.4%  GPN = 1.4% |
| Overwater 2017 [23] | Netherlands | NR | Children with TSC (median age 8.2 years; 86% had epilepsy) | 102 | Median number of ASMs = 1  Corticosteroids = 5.0%  VGB as first ASM = 32.0%  VGB as second or later ASM = 29.0% |
|  | Netherlands | NR | Children with TSC and epilepsy (median age 8.0 years) | 88 | Median number of ASMs = 2  Corticosteroids = 6.0%  VGB as first ASM = 22.0%  VGB as second or later ASM = 34.0% |
| Van Andel 2020 [119] | Netherlands | 1990–2015 | Children and adults with TSC (aged 8–21 years; 66.7% had epilepsy) | 15 | 1 ASM = 53.3%  2 ASMs = 20.0%  3 ASMs = 6.7% |
| Vergeer 2019 [60] | Netherlands | NR | Adults (median age 42–46 years) with TSC and epilepsy – refractory epilepsy for 100% of follow-up | 49 | Mean (SD) number of ASMs = 5 (2.7) |
|  | Netherlands | NR | Adults (median age 42–46 years) with TSC and epilepsy – refractory epilepsy for 50–99.9% of follow-up | 81 | Mean (SD) number of ASMs = 5.6 (2.9) |
|  | Netherlands | NR | Adults (median age 42–46 years) with TSC and epilepsy – refractory epilepsy for 0.1–50% of follow-up | 44 | Mean (SD) number of ASMs = 3.5 (1.8) |
|  | Netherlands | NR | Adults (median age 42–46 years) with TSC and epilepsy – refractory epilepsy for 0% of follow-up | 63 | Mean (SD) number of ASMs = 1.6 (1.2) |
| Kotulska 2014 [39] | Poland | NR | Children with TSC developing seizures as neonate^†^ | 21 | EVE = 14.3%  Epilepsy surgery = 19.1%  VGB = 76.2%  ACTH = 42.9%  VPA = 61.9%  CBZ = 19.1%  PB = 38.1%  LEV = 33.3%  LTG = 4.8%  TPM = 19.1%  OXC = 4.8%  PHT = 19.1%  NZP = 14.3%  DZP = 9.5%  VNS = 4.8% |
| Sadowski 2022 [120] | Poland | 2016–2019 | Children with TSC and epilepsy (aged 1–14 years) | 32 | Median (range) number of currently prescribed ASMs = 3 (1–4)  VGB = 87.5%  VPA = 71.9%  TPM = 31.3%  LTG = 28.1%  LEV = 25.0%  CLB = 15.6%  OXC = 3.1% |
| Orazem Mrak 2017 [22] | Slovenia | NR | Children with TSC and epilepsy (IS)^†^ | 8 | VGB = 75.0%  Hydrocortisone = 25.0% |
|  | Slovenia | NR | Children with TSC and focal epilepsy^†^ | 13 | ASMs = 54.0%  1 ASM = 38.0%  2 ASMs = 15.0% |
| Gallardo-Tur 2013 [121] | Spain | NR | Adults (aged >14 years) with TSC and epilepsy | 16 | 1 ASM = 38.0%  2 ASMs = 44.0%  3 ASMs = 19.0% |
| Giacaman 2017 [46] | Spain | NR | Children with TSC (aged 7 months to 15 years; 39% had epilepsy) | 31 | ≥1 ASM = 39.0%  Topical rapamycin =13.0%  Oral mTOR inhibitors = 13.0% |
| Marques 2019 [91] | Spain | NR | Children and adults with TSC and focal epilepsy (aged 0–71 years) | 86 | mTOR inhibitors = 9.3%  Epilepsy surgery = 10.5%  GABAergics = 73.3%  ACTH = 2.3%  Ketogenic diet = 7.0%  Fructose derivatives = 0%  VNS = 5.8%  Other treatments = 82.6% |
| Puertas-Martin 2014 [122] | Spain | NR | Children (aged <18 years) with TSC and epilepsy | 30 | 1 ASM = 37.7%  2 ASMs = 33.3%  3 ASMs = 23.3%  4 ASMs = 3.3%  ACTH = 6.7%  VNS = 6.7%  EVE = 13.3%  Epilepsy surgery = 26.7% |
| Marques 2019 [91] | Sweden | NR | Children and adults with TSC and focal epilepsy (aged 0–71 years) | 23 | mTOR inhibitors = 34.8%  Epilepsy surgery = 26.1%  GABAergics = 100%  ACTH = 4.3%  Ketogenic diet = 26.1%  Fructose derivatives = 4.3%  VNS = 8.7%  Other treatments = 95.7% |
| Welin 2017 [24] | Sweden | NR | Children and adults with TSC and refractory epilepsy (aged 0–>65 years) | 386 | ASMs = 97.9%  VPA = 45.1%  LTG = 43.3%  CBZ = 37.6%  LEV = 36.5%  TPM = 24.1%  Anxiolytic treatment = 72.5%  DZP = 64.5%  Neuroleptic treatment = 16.6%  Risperidone = 11.4%  Psychoanaleptic treatment = 23.6%  Methylphenidate = 7.3%  mTOR inhibitors = 15.3%  Sirolimus = 11.4%  SEGA surgery = 8.6%  Epilepsy surgery = 6.5%  Ketogenic diet = 1.6%  VNS = 6.0% |
| Crawford 2015 [16] | UK | 1997–2012 | Patients with TSC treated for epilepsy^†^ | 200 | CBZ = 56%  VPA = 46%  LTG = 34%  VGB = 26% |
| Marques 2019 [91] | UK | NR | Children and adults with TSC and focal epilepsy (aged 0–71 years) | 12 | mTOR inhibitors = 0%  Epilepsy surgery = 0%  GABAergics = 50.0%  ACTH = 8.3%  Ketogenic diet = 0%  Fructose derivatives = 41.7%  VNS = 0%  Other treatments = 58.3% |
| Shepherd 2017 [51] | UK | 1987–2013 | Children and adults with TSC with epilepsy (mean age 26.8 years) | 209 | 0 ASMs = 12.0%  1 ASM = 28.2%  2 ASMs = 31.6%  3 ASMs = 22.0%  ≥4 ASMs = 6.2% |
|  |  |  | Children (aged <18 years) with TSC with epilepsy | 81 | 0 ASMs = 12.4%  1 ASM = 29.6%  2 ASMs = 35.8%  3 ASMs = 18.5%  ≥4 ASMs = 3.7% |
|  |  |  | Adults (aged ≥18 years) with TSC and epilepsy | 128 | 0 ASMs = 11.7%  1 ASM = 27.3%  2 ASMs = 28.9%  3 ASMs = 24.2%  ≥4 ASMs = 7.8% |
| Tierney 2011 [59] | UK | NR | Adults (aged 22–56 years) with TSC with controlled epilepsy | 11 | 1 ASM = 63.6%   - CBZ = 36.4% - VPA = 9.1% - TPM = 9.1% - PHT = 9.1%   2 ASMs = 36.4% |
| Tye 2018 [123] | UK | NR | Children and adults with TSC  (aged 6–70 years; 44% had a history of epileptic spasms) – Cardiff cohort | 98 | VGB = 20.0% |
|  |  |  | Children with TSC (aged 0–16 years; 49% had a history of epileptic spasms) – TS 2000 cohort | 125 | VGB = 51.0% |
| Carpio 2014 [124] | Venezuela | NR | Children (aged <18 years) with TSC and epilepsy | 23 | EVE = 13.0% |

^†^Population age not defined

ACTH: adrenocorticotropic hormone; ASM: antiseizure medication; BRV: brivaracetam; BZD: benzodiazepine; CBD: cannabidiol; CBZ: carbamazepine; CC: corpus callosotomy; CLB: clobazam; CRM: cardiac rhabdomyoma; CZP: clonazepam; DRE: drug-resistant epilepsy; DZP: diazepam; EEG: electroencephalography; ER: extended release; ES: epileptic spasm; ESL: eslicarbazepine; ESM: ethosuximide; EVE: everolimus; FBM: felbamate; FR: frontal resection; FS: focal seizure; GABA: gamma-aminobutyric acid; GP: general practitioner; GPN: gabapentin; IS: infantile spasm; LCM: lacosamide; LEV: levetiracetam; LTG: lamotrigine; MDZ: midazolam; MR: multilobar resection; mTOR: mammalian target of rapamycin; NA: not applicable; NR: not reported; NZP: nitrazepam; ODT: orally disintegrating tablet; OXC: oxcarbazepine; PB: phenobarbital; PER: perampanel; PGB: pregabalin; PHT: phenytoin; PPY: per patient year; PR: parietal resection; PRM: primidone; RFM: rufinamide; SD: standard deviation; SE: status epilepticus; SEGA: subependymal giant cell astrocytoma; SEN: subependymal nodule; TCC: total corpus callosotomy; TPM: topiramate; TR: temporal resection; TSC: tuberous sclerosis complex; VGB: vigabatrin; VNS: vagus nerve stimulation; VPA: valproic acid; ZNS: zonisamide

# References

1. Fleury P, van Schooneveld M, Delleman J, van Baal J. Neurological, ophthalmological and nephrological aspects of tuberous sclerosis. Tuberous sclerosis and neurofibromatosis: epidemiology, pathophysiology, biology and management. Amsterdam: Elsevier; 1990. p. 221–6.

2. Hunt A. Development, behaviour and seizures in 300 cases of tuberous sclerosis. J Intellect Disabil Res. 1993;37(Pt 1):41–51.

3. Webb DW, Fryer AE, Osborne JP. Morbidity associated with tuberous sclerosis: a population study. Dev Med Child Neurol. 1996;38:146–55.

4. Joinson C, O’Callaghan FJ, Osborne JP, Martyn C, Harris T, Bolton PF. Learning disability and epilepsy in an epidemiological sample of individuals with tuberous sclerosis complex. Psychol Med. 2003;33:335–44.

5. Winterkorn EB, Pulsifer MB, Thiele EA. Cognitive prognosis of patients with tuberous sclerosis complex. Neurology. 2007;68:62–4.

6. Muzykewicz DA, Newberry P, Danforth N, Halpern EF, Thiele EA. Psychiatric comorbid conditions in a clinic population of 241 patients with tuberous sclerosis complex. Epilepsy Behav. 2007;11:506–13.

7. Chu-Shore CJ, Major P, Camposano S, Muzykewicz D, Thiele EA. The natural history of epilepsy in tuberous sclerosis complex. Epilepsia. 2010;51:1236–41.

8. Numis AL, Major P, Montenegro MA, Muzykewicz DA, Pulsifer MB, Thiele EA. Identification of risk factors for autism spectrum disorders in tuberous sclerosis complex. Neurology. 2011;76:981–7.

9. Hallett L, Foster T, Liu Z, Blieden M, Valentim J. Burden of disease and unmet needs in tuberous sclerosis complex with neurological manifestations: systematic review. Curr Med Res Opin. 2011;27:1571–83.

10. Wataya-Kaneda M, Tanaka M, Hamasaki T, Katayama I. Trends in the prevalence of tuberous sclerosis complex manifestations: an epidemiological study of 166 Japanese patients. PLoS One. 2013;8:e63910.

11. Pashos CL, Rentz A, Liu J, Pelletier C, Prestifilippo J, Nakagawa J, et al. PSU33 Characteristics and burden of tuberous sclerosis complex: results of a patient and caregiver survey in the United States. Value Health. 2012;15:A408.

12. Sun P, Liu Z, Kohrman M. Prevalence and incidence rates of most common comorbid conditions among patients with tuberous sclerosis complex: a national claim database analysis. Value Health. 2012;15(4):A193 (Abstract PIH11).

13. van Eeghen AM, Chu-Shore CJ, Pulsifer MB, Camposano SE, Thiele EA. Cognitive and adaptive development of patients with tuberous sclerosis complex: a retrospective, longitudinal investigation. Epilepsy Behav. 2012;23:10–5.

14. Vignoli A, La Briola F, Turner K, Scornavacca G, Chiesa V, Zambrelli E, et al. Epilepsy in TSC: certain etiology does not mean certain prognosis. Epilepsia. 2013;54:2134–42.

15. Wilbur C, Sanguansermsri C, Chable H, Anghelina M, Peinhof S, Anderson K, et al. Manifestations of tuberous sclerosis complex: the experience of a provincial clinic. Can J Neurol Sci. 2017;44:35–43.

16. Crawford P, Shepherd C, Demuth D, Nasuti P, Lucchese L, Haider Z, et al. Real-world assessment of the burden of epilepsy in tuberous sclerosis complex (TSC) patients in the United Kingdom (UK). Epilepsy Curr. 2015;15(1 Suppl 1):446 (Abstract 3.126).

17. Vignoli A, La Briola F, Peron A, Turner K, Vannicola C, Saccani M, et al. Autism spectrum disorder in tuberous sclerosis complex: searching for risk markers. Orphanet J Rare Dis. 2015;10:154.

18. Wilson TA, Rodgers S, Tanweer O, Agarwal P, Lieber BA, Agarwal N, et al. Tuberous sclerosis health care utilization based on the national inpatient sample database: a review of 5655 hospitalizations. World Neurosurg. 2016;91:97–105.

19. Strzelczyk A, Rosenow F, Zöllner JP, Simon A, Wyatt G, Holland R, et al. Epidemiology, healthcare resource use, and mortality in patients with tuberous sclerosis complex: a population-based study on German health insurance data. Seizure. 2021;91:287–95.

20. Chung CW, Lawson JA, Sarkozy V, Riney K, Wargon O, Shand AW, et al. Early detection of tuberous sclerosis complex: an opportunity for improved neurodevelopmental outcome. Pediatr Neurol. 2017;76:20–6.

21. Jeong A, Nakagawa JA, Wong M. Predictors of drug-resistant epilepsy in tuberous sclerosis complex. J Child Neurol. 2017;32:1092–8.

22. Orazem Mrak J, Neubauer D, Perkovic Benedik M. Epilepsy in children with tuberous sclerosis complex: Ljubljana University Children’s Hospital experience. Epilepsia. 2017;58:S190-S191 (Abstract p1069).

23. Overwater IE, Verhaar BJ, Lingsma HF, Bindels-de Heus GC, van den Ouweland AM, Nellist M, et al. Interdependence of clinical factors predicting cognition in children with tuberous sclerosis complex. J Neurol. 2017;264:161–7.

24. Welin K-O, Carlqvist P, Svensson A, Althin R, Eklund E, Rask O. Epilepsy in tuberous sclerosis patients in Sweden – healthcare utilization, treatment, morbidity, and mortality using national register data. Seizure. 2017;53:4–9.

25. Song J, Swallow E, Said Q, Peeples M, Meiselbach M, Signorovitch J, et al. Epilepsy treatment patterns among patients with tuberous sclerosis complex. J Neurol Sci. 2018;391:104–8.

26. Farach LS, Richard MA, Lupo PJ, Sahin M, Krueger DA, Wu JY, et al. Epilepsy risk prediction model for patients with tuberous sclerosis complex. Pediatr Neurol. 2020;113:46–50.

27. Nabbout R, Belousova E, Benedik MP, Carter T, Cottin V, Curatolo P, et al. Epilepsy in tuberous sclerosis complex: findings from the TOSCA study. Epilepsia Open. 2019;4:73–84.

28. Erdal Y, Alnak A, Topaloglu Tekturk P, Yapici Z. EPO1091 Clinical characteristics of tuberoussclerosis patients with refractory epilepsy. Eur J Neurol. 2020;27:577.

29. Grau J, Zöllner JP, Schubert-Bast S, Kurlemann G, Hertzberg C, Wiemer-Kruel A, et al. Direct and indirect costs and cost-driving factors of tuberous sclerosis complex in children, adolescents, and caregivers: a multicenter cohort study. Orphanet J Rare Dis. 2021;16:282.

30. Nabbout R, Belousova E, Benedik MP, Carter T, Cottin V, Curatolo P, et al. Historical patterns of diagnosis, treatments, and outcome of epilepsy associated with tuberous sclerosis complex: results from TOSCA registry. Front Neurol. 2021;12:697467.

31. Peng J-H, Tu H-P, Hong C-H. A population-based study to estimate survival and standardized mortality of tuberous sclerosis complex (TSC) in Taiwan. Orphanet J Rare Dis. 2021;16:335.

32. Rok P, Kasprzyk-Obara J, Domańska-Pakieła D, Jóźwiak S. Clinical symptoms of tuberous sclerosis complex in patients with an identical TSC2 mutation. Med Sci Monit. 2005;11:CR230-234.

33. de Vries PJ, Hunt A, Bolton PF. The psychopathologies of children and adolescents with tuberous sclerosis complex (TSC): a postal survey of UK families. Eur Child Adolesc Psychiatry. 2007;16:16–24.

34. Hong C-H, Tu H-P, Lin J-R, Lee C-H. An estimation of the incidence of tuberous sclerosis complex in a nationwide retrospective cohort study (1997–2010). Br J Dermatol. 2016;174:1282–9.

35. Demuth D, Nasuti P, Richards C, Gray L, Price L, Magestro M. PRM40 Use of the clinical practice research datalink (CPRD) to assess “real-world” management of tuberous sclerosis complex (TSC) in the United Kingdom. Value Health. 2013;16:A581–2.

36. O’Connor A, Chopra M, Kennedy S, Mohammed S, Wilson M, Mowat D, et al. Epidemiology of tuberous complex: a study of new diagnoses in children 2004-2009 (Abstract C2-0005 presented at the 12th International Child Neurology Congress and the 11th Asian and Oceanian Congress of Child Neurology, 27 May - 1 June, Brisbane, Australia). Dev Med Child Neurol. 2012;54:74–5.

37. Graffigna G, Bosio C, Cecchini I. Assisting a child with tuberous sclerosis complex (TSC): a qualitative deep analysis of parents’ experience and caring needs. BMJ Open. 2013;3:e003707.

38. Amin S, Mallick AA, Lux A, O’Callaghan F. Quality of life in patients with tuberous sclerosis complex (TSC). Eur J Paediatr Neurol. 2019;23:801–7.

39. Kotulska K, Jurkiewicz E, Domańska-Pakieła D, Grajkowska W, Mandera M, Borkowska J, et al. Epilepsy in newborns with tuberous sclerosis complex. Eur J Paediatr Neurol. 2014;18:714–21.

40. Gumbelevičiene L, Endziniene M, Vaičiene-Magistris N. Epilepsy in a cohort of children with tuberous sclerosis complex. Epilepsia. 2014;55:142.

41. Rentz AM, Skalicky AM, Liu Z, Wheless JW, Dunn DW, Frost MD, et al. Tuberous sclerosis complex: a survey of health care resource use and health burden. Pediatr Neurol. 2015;52:435–41.

42. Wheless JW, Engel-Nitz NM, Johnson JC, Said Q, Gibson TT. Seizures and other clinical manifestations prior to diagnosis of tuberous sclerosis complex. Abstract presented at the American Epilepsy Society (AES) Annual Meeting, Houston, TX, USA, 2–6 December 2016 (Abstract 3.342) [Internet]. 2016. Available from: https://aesnet.org/abstractslisting/seizures-and-other-clinical-manifestations-prior-to-diagnosis-of-tuberous-sclerosis-complex

43. Vorgia P, Karahaliou M, Dulios G, Niotakis G, Galanakis E. Frequency of tuberous sclerosis complex diagnosis in pediatric patients the last 15 years in the island of Crete. Epilepsia. 2016;57:103–4.

44. da Câmara RP, Costa MB, Rodrigues M, Jacinto S, Vieira JP, Amorim M, et al. Tuberous sclerosis – the need for a multidisciplinary management program. Cogent Med. 2016;3(1):1265203 (Abstract 207).

45. Kingswood C, Bolton P, Crawford P, Harland C, Johnson SR, Sampson JR, et al. The clinical profile of tuberous sclerosis complex (TSC) in the United Kingdom: a retrospective cohort study in the Clinical Practice Research Datalink (CPRD). Eur J Paediatr Neurol. 2016;20:296–308.

46. Giacaman A, Escudero-Gongora M, Bauza A, Salinas J, Rosell J, Roldan J, et al. Clinical and genetic findings in 31 patients with tuberous sclerosis complex. Pediatr Dermatol. 2017;34(Suppl 1):S130–1.

47. Capal JK, Bernardino-Cuesta B, Horn PS, Murray D, Byars AW, Bing NM, et al. Influence of seizures on early development in tuberous sclerosis complex. Epilepsy Behav. 2017;70:245–52.

48. Davis PE, Filip-Dhima R, Sideridis G, Peters JM, Au KS, Northrup H, et al. Presentation and diagnosis of tuberous sclerosis complex in infants. Pediatrics. 2017;140:e20164040.

49. Jansen AC, Belousova E, Benedik MP, Carter T, Cottin V, Curatolo P, et al. Quality of life and burden of disease in tuberous sclerosis complex (TSC): findings from TOSCA research project. J Intellect Disabil Res. 2017;61:829 (Abstract).

50. Patel A, Watchko S, Nellesen D, Herbst F, Neary M. The natural history and burden of illness of epilepsy in tuberous sclerosis complex (TSC): a systematic literature review. Eur J Paediatr Neurol. 2017;21:e186–7.

51. Shepherd C, Koepp M, Myland M, Patel K, Miglio C, Siva V, et al. Understanding the health economic burden of patients with tuberous sclerosis complex (TSC) with epilepsy: a retrospective cohort study in the UK Clinical Practice Research Datalink (CPRD). BMJ Open. 2017;7:e015236.

52. de Groen A-EC, Bolton J, Bergin AM, Sahin M, Peters JM. The evolution of subclinical seizures in children with tuberous sclerosis complex. J Child Neurol. 2019;34:770–7.

53. Schoenberger A, Capal JK, Ondracek A, Horn PS, Murray D, Byars AW, et al. Language predictors of autism spectrum disorder in young children with tuberous sclerosis complex. Epilepsy Behav. 2020;103:106844.

54. Wu JY, Goyal M, Peters JM, Krueger D, Sahin M, Northrup H, et al. Scalp EEG spikes predict impending epilepsy in TSC infants: a longitudinal observational study. Epilepsia. 2019;60:2428–36.

55. Hulshof HM, Brenner J, Overwater IE, de Wit M-C, Braun KP, Jansen FE. Counselling in tuberous sclerosis complex: a survey on content and satisfaction in the Netherlands. Eur J Paediatr Neurol. 2020;25:113–9.

56. Ding Y, Zhou Y, Yu L, Zhang L, Zhou S, Wang Y, et al. Correlation between epilepsy and genotype: a large retrospective tuberous sclerosis complex cohort. Seizure. 2021;91:273–7.

57. Ihnen SK, Capal JK, Horn PS, Griffith M, Sahin M, Bebin EM, et al. Epilepsy is heterogeneous in early-life tuberous sclerosis complex. Pediatr Neurol. 2021;123:1–9.

58. Staley BA, Vail EA, Thiele EA. Tuberous sclerosis complex: diagnostic challenges, presenting symptoms, and commonly missed signs. Pediatrics. 2011;127:e117–25.

59. Tierney KM, McCartney DL, Serfontein JR, de Vries PJ. Neuropsychological attention skills and related behaviours in adults with tuberous sclerosis complex. Behav Genet. 2011;41:437–44.

60. Vergeer M, de Ranitz-Greven WL, Neary MP, Ionescu-Ittu R, Emond B, Sheng Duh M, et al. Epilepsy, impaired functioning, and quality of life in patients with tuberous sclerosis complex. Epilepsia Open. 2019;4:581–92.

61. Li W, Peng A, Qiu X, Zhang L, Lai W, Chen L. Risk factors for drug-resistant epilepsy in adult patients with tuberous sclerosis. Epilepsia. 2019;60:209–10.

62. Vignoli A, La Briola F, Turner K, Peron A, Vannicola C, Chiesa V, et al. Epilepsy in adult patients with tuberous sclerosis complex. Acta Neurol Scand. 2021;144:29–40.

63. Zöllner JP, Grau J, Rosenow F, Sauter M, Knuf M, Kurlemann G, et al. Direct and indirect costs and cost-driving factors in adults with tuberous sclerosis complex: a multicenter cohort study and a review of the literature. Orphanet J Rare Dis. 2021;16:250.

64. Bar C, Ghobeira R, Azzi R, Ville D, Riquet A, Touraine R, et al. Experience of follow-up, quality of life, and transition from pediatric to adult healthcare of patients with tuberous sclerosis complex. Epilepsy Behav. 2019;96:23–7.

65. Liang S, Li A, Zhao M, Jiang H, Yu S, Meng X, et al. Epilepsy surgery in tuberous sclerosis complex: emphasis on surgical candidate and neuropsychology. Epilepsia. 2010;51:2316–21.

66. Liang S, Zhang J, Yang Z, Zhang S, Cui Z, Cui J, et al. Long-term outcomes of epilepsy surgery in tuberous sclerosis complex. J Neurol. 2017;264:1146–54.

67. Pearsson K, Compagno-Strandberg M, Eklund EA, Rask O, Källén K. Satisfaction and seizure outcomes of epilepsy surgery in tuberous sclerosis: a Swedish population-based long-term follow-up study. Seizure. 2022;103:39–45.

68. Zaroff CM, Morrison C, Ferraris N, Weiner HL, Miles DK, Devinsky O. Developmental outcome of epilepsy surgery in tuberous sclerosis complex. Epileptic Disord. 2005;7:321–6.

69. Liu S, Yu T, Guan Y, Zhang K, Ding P, Chen L, et al. Resective epilepsy surgery in tuberous sclerosis complex: a nationwide multicentre retrospective study from China. Brain. 2020;143:570–81.

70. de Vries PJ, Franz DN, Curatolo P, Nabbout R, Neary M, Herbst F, et al. Measuring health-related quality of life in tuberous sclerosis complex – psychometric evaluation of three instruments in individuals with refractory epilepsy. Front Pharmacol. 2018;9:964.

71. Zöllner JP, Conradi N, Sauter M, Knuf M, Knake S, Kurlemann G, et al. Quality of life and its predictors in adults with tuberous sclerosis complex (TSC): a multicentre cohort study from Germany. Neurol Res Pract. 2021;3:35.

72. Moavero R, Voci A, Romigi A, Bisulli F, Luisi C, Matricardi S, et al. Questionnaire-based assessment of sleep disorders in an adult population of tuberous sclerosis complex. Sleep Med. 2022;92:81–7.

73. Zamponi N, Petrelli C, Passamonti C, Moavero R, Curatolo P. Vagus nerve stimulation for refractory epilepsy in tuberous sclerosis. Pediatr Neurol. 2010;43:29–34.

74. Kadish NE, Riedel C, Stephani U, Wiegand G. Developmental outcomes in children/adolescents and one adult with tuberous sclerosis complex (TSC) and refractory epilepsy treated with everolimus. Epilepsy Behav. 2020;111:107182.

75. Thiele EA, Bebin EM, Bhathal H, Jansen FE, Kotulska K, Lawson JA, et al. Add-on cannabidiol treatment for drug-resistant seizures in tuberous sclerosis complex: a placebo-controlled randomized clinical trial. JAMA Neurol. 2021;78:285–92.

76. Bauer D, Fountain N. Seizure characteristics in adult tuberous sclerosis. Abstract presented at the American Epilepsy Society (AES) Annual Meeting, Houston, TX, USA, 2–6 December 2016 (Abstract 2.067) [Internet]. 2016 [cited 2024 Jan 31]. Available from: https://aesnet.org/abstractslisting/seizure-characteristics-in-adult-tuberous-sclerosis

77. Betts KA, Stockl KM, Yin L, Hollenack K, Wang M-J, Yang X. Economic burden associated with tuberous sclerosis complex in patients with epilepsy. Epilepsy Behav. 2020;112:107494.

78. Hsieh DT, Jennesson MM, Thiele EA. Epileptic spasms in tuberous sclerosis complex. Epilepsy Res. 2013;106:200–10.

79. Husain AM, Foley CM, Legido A, Chandler DA, Miles DK, Grover WD. West syndrome in tuberous sclerosis complex. Pediatr Neurol. 2000;23:233–5.

80. Lennert B, Farrelly E, Sacco P, Pira G, Frost M. Resource utilization in children with tuberous sclerosis complex and associated seizures: a retrospective chart review study. J Child Neurol. 2013;28:461–9.

81. Reaven NL, Funk SE, Lyons PD, Story TJ. The direct cost of seizure events in severe childhood-onset epilepsies: a retrospective claims-based analysis. Epilepsy Behav. 2019;93:65–72.

82. Rentz AM, Skalicky AM, Pashos CL, Liu Z, Magestro M, Pelletier CL, et al. Caring for children with tuberous sclerosis complex: what is the physical and mental health impact on caregivers? J Child Neurol. 2015;30:1574–81.

83. Skalicky AM, Rentz AM, Liu Z, Wheless JW, Pelletier CL, Dunn DW, et al. The burden of subependymal giant cell astrocytomas associated with tuberous sclerosis complex: results of a patient and caregiver survey. J Child Neurol. 2015;30:563–9.

84. Skalicky AM, Rentz AM, Liu Z, Said Q, Nakagawa JA, Frost MD, et al. Economic burden, work, and school productivity in individuals with tuberous sclerosis and their families. J Med Econ. 2018;21:953–9.

85. Stockl K, Funk S, Reaven N, Hollenack K. Inpatient hospitalizations and readmissions among patients with probable Lannox-Gastaut syndrome, Dravet syndrome, tuberous sclerosis complex, and other refractory epilepsies. J Manag Care Spec Pharm. 2019;25:S57-8 (Abstract G20).

86. Sun P, Liu Z, Krueger D, Kohrman M. Direct medical costs for patients with tuberous sclerosis complex and surgical resection of subependymal giant cell astrocytoma: a US national cohort study. J Med Econ. 2015;18:349–56.

87. Deverell M, Phu A, Elliott EJ, Teutsch SM, Eslick GD, Stuart C, et al. Health-related out-of-pocket expenses for children living with rare diseases - tuberous sclerosis and mitochondrial disorders: a prospective pilot study in Australian families. J Paediatr Child Health. 2022;58:611–7.

88. Chu WC-Y, Chiang LL-W, Chan DC-C, Wong WH-S, Chan GC-F. Prevalence, mortality and healthcare economic burden of tuberous sclerosis in Hong Kong: a population-based retrospective cohort study (1995-2018). Orphanet J Rare Dis. 2020;15:264.

89. Jansen AC, Vanclooster S, de Vries PJ, Fladrowski C, Beaure d’Augères G, Carter T, et al. Burden of illness and quality of life in tuberous sclerosis complex: findings from the TOSCA study. Front Neurol. 2020;11:904.

90. Fagnani F, Laurendeau C, de Zelicourt M, Marshall J. Epidemiology and disease burden of tuberous sclerosis complex in France: a population-based study based on national health insurance data. Epilepsia Open. 2022;7:633–44.

91. Marques R, Belousova E, Benedik MP, Carter T, Cottin V, Curatolo P, et al. Treatment patterns and use of resources in patients with tuberous sclerosis complex: insights from the TOSCA registry. Front Neurol. 2019;10:1144.

92. Waltereit R, Beaure d’Augères G, Jancic J, Kingswood JC, Koleva M, Marques R, et al. Involvement of mental health professionals in the treatment of tuberous sclerosis complex-associated neuropsychiatric disorders (TAND): results of a multinational European electronic survey. Orphanet J Rare Dis. 2021;16:216.

93. Lesniowska J, Kotulska K, Jóźwiak S. PRO31 Medical innovation assessment and economic burden of diseases on the example of rare and common diseases in Poland. Value Health. 2019;22:S846.

94. Demuth D, Nasuti P, Lucchese L, Gray L, Pinnegar A, Magestro M. Economic impact of patients with tuberous sclerosis complex (TSC) in the UK: a retrospective database analysis in the clinical practice research datalink (CPRD). Value Health. 2014;17(3):A137 (Abstract PHS69).

95. Kingswood JC, Nasuti P, Patel K, Myland M, Siva V, Gray E. The economic burden of tuberous sclerosis complex in UK patients with renal manifestations: a retrospective cohort study in the clinical practice research datalink (CPRD). J Med Econ. 2016;19:1116–26.

96. Karakas C, Coorg R, Curry D. Ictal onset of seizures recorded on stereo-EEG in children with tuberous sclerosis complex. J Clin Neurophysiol. 2020;37:323–4.

97. Frost MD, Rentz AM, Pashos CL, Liu J, Pelletier C, Prestifilippo J, et al. Characteristics and burden of seizures in patients with tuberous sclerosis complex: results of a patient and caregiver survey in the US. Epilepsy Curr. 2013;13(Suppl. 1):S318 (Abstract 2.337).

98. Valentim J. Long-term disabiliy cost in tuberous sclerosis complex (TSC) in Brazil. Value Health. 2010;13(7):A389 (Abstract PND10).

99. Skrobanski H, Vyas K, Bowditch S, Hubig L, Dziadulewicz E, Fish L, et al. The burden of caring for individuals with tuberous sclerosis complex (TSC) who experience epileptic seizures: a descriptive UK survey. Pharmacoecon Open. 2023;7:299–312.

100. Skrobanski H, Vyas K, Bowditch S, Hubig L, Dziadulewicz E, Fish L, et al. Shared decision-making and the caregiver experience in tuberous sclerosis complex: results from a UK survey. Orphanet J Rare Dis. 2023;18:78.

101. Patel S, Grinspoon R, Fleming B, Skirvin LA, Wade C, Wolper E, et al. The long-term efficacy of cannabidiol in the treatment of refractory epilepsy. Epilepsia. 2021;62:1594–603.

102. Peters JM, Grayson L, Krueger D, Sahin M, Wu J. Early epilepsy surgery in tuberous sclerosis complex: preliminary results on safety, efficacy and neurodevelopmental outcomes. Abstract presented at the American Epilepsy Society (AES) Aunnual Meeting, New Orleans, LA, USA, 30 November–4 December 2018 [Internet]. 2018 p. (Abstract 3.456). Available from: https://aesnet.org/abstractslisting/early-epilepsy-surgery-in-tuberous-sclerosis-complex--preliminary-results-on-safety--efficacy-and-neurodevelopmental-outcomes

103. Samueli S, Abraham K, Dressler A, Gröppel G, Mühlebner-Fahrngruber A, Feucht M. The role of mTOR inhibitors in TSC associated epilepsy. Eur J Paediatr Neurol. 2015;19:S17 (Abstract OP53-2462).

104. Samueli S, Abraham K, Dressler A, Gröppel G, Mühlebner-Fahrngruber A, Scholl T, et al. Efficacy and safety of everolimus in children with TSC - associated epilepsy - pilot data from an open single-center prospective study. Orphanet J Rare Dis. 2016;11:145.

105. Whitney R, Zak M, Haile D, Nabavi Nouri M. The state of pediatric tuberous sclerosis complex epilepsy care: results from a national survey. Epilepsia Open. 2022;7:718–28.

106. Zhao X, Jiang D, Hu Z, Yang J, Liang D, Yuan B, et al. Machine learning and statistic analysis to predict drug treatment outcome in pediatric epilepsy patients with tuberous sclerosis complex. Epilepsy Res. 2022;188:107040.

107. Baumgartner T, Carreño M, Rocamora R, Bisulli F, Boni A, Brázdil M, et al. A survey of the European Reference Network EpiCARE on clinical practice for selected rare epilepsies. Epilepsia Open. 2021;6:160–70.

108. Ebrahimi-Fakhari D, Hussong J, Flotats-Bastardas M, Ebrahimi-Fakhari D, Zemlin M, von Gontard A, et al. Tuberous sclerosis complex associated neuropsychiatric disorders and parental stress: findings from a national, prospective TSC surveillance study. Neuropediatrics. 2019;50:294–9.

109. Hamer H, Kasper B, May T, Mayer T, Straub H-B, Steinhoff BJ, et al. Characteristics and health care of adult tuberous sclerosis vomplex (TSC) patients in German epilepsy centers. Epilepsia. 2016;57(Suppl. 2):70 (Abstract P198).

110. Strzelczyk A, Grau J, Bast T, Bertsche A, Bettendorf U, Hahn A, et al. Prescription patterns of antiseizure drugs in tuberous sclerosis complex (TSC)-associated epilepsy: a multicenter cohort study from Germany and review of the literature. Expert Rev Clin Pharmacol. 2021;14:749–60.

111. Willems LM, Rosenow F, Schubert-Bast S, Kurlemann G, Zöllner JP, Bast T, et al. Efficacy, retention and tolerability of everolimus in patients with tuberous sclerosis complex: a survey-based study on patients’ perspectives. CNS Drugs. 2021;35:1107–22.

112. Kingswood JC, d’Augères GB, Belousova E, Ferreira JC, Carter T, Castellana R, et al. TuberOus SClerosis registry to increase disease Awareness (TOSCA) – baseline data on 2093 patients. Orphanet J Rare Dis. 2017;12:2.

113. Słowińska M, Kotulska K, Szymańska S, Roberds SL, Fladrowski C, Jóźwiak S. Approach to preventive epilepsy treatment in tuberous sclerosis complex and current clinical practice in 23 countries. Pediatr Neurol. 2021;115:21–7.

114. Shlomovitz O, Ben-Zeev B, Pleniceanu O, Greenberger S, Lahav E, Mini S, et al. An Israeli tuberous sclerosis cohort: the efficacy of different anti-epileptic strategies. Childs Nerv Syst. 2021;37:3827–33.

115. Mingarelli A, Caputo D, La Briola F, Savini MN, Cervi F, Peron A, et al. Early seizure onset in TSC: probing for prognostic markers. Epilepsia. 2018;59:S298 (Abstract p694).

116. LoPresti M, Murofushi T, Claxton L, Marshall J. PND14 Identifying patients with RARE refractory epilepsies in Japanese health databases: feasibility for a burden of illness study. Value Health Reg Issues. 2020;22(Suppl):S77.

117. Hulshof HM, Slot EMH, Lequin M, Breuillard D, Boddaert N, Jozwiak S, et al. Fetal brain magnetic resonance imaging findings predict neurodevelopment in children with tuberous sclerosis complex. J Pediatr. 2021;233:156-162.e2.

118. Overwater IE, Heus KB, Rietman AB, Hoopen LW ten, Vergouwe Y, Moll HA, et al. Epilepsy in children with tuberous sclerosis complex: chance of remission and response to antiepileptic drugs. Epilepsia. 2015;56:1239–45.

119. van Andel DM, Sprengers JJ, Oranje B, Scheepers FE, Jansen FE, Bruining H. Effects of bumetanide on neurodevelopmental impairments in patients with tuberous sclerosis complex: an open-label pilot study. Mol Autism. 2020;11:30.

120. Sadowski K, Sijko K, Domańska-Pakieła D, Borkowska J, Chmielewski D, Ulatowska A, et al. Antiepileptic effect and safety profile of rapamycin in pediatric patients with tuberous sclerosis complex. Front Neurol. 2022;13:704978.

121. Gallardo-Tur A, García-Martín G, Chamorro-Muñoz MI, Romero-Godoy J, Romero-Acebal M. Control of epilepsy in adult patients with tuberous sclerosis. Rev Neurol. 2013;56:562–6.

122. Puertas-Martin V, Carreras-Saez I, Marana A, Ruiz-Falco Rojas ML, Cantarin-Extremera V, Calleja-Gero ML. Therapeutic possibilities in refractory epilepsy in tuberous sclerosis complex. Rev Neurol. 2014;58:529–35.

123. Tye C, Thomas LE, Sampson JR, Lewis J, O’Callaghan F, Yates JR, et al. Secular changes in severity of intellectual disability in tuberous sclerosis complex: a reflection of improved identification and treatment of epileptic spasms? Epilepsia Open. 2018;3:276–80.

124. Carpio A, Ravelo ME, Rodriguez N, Sanchez A. Characterization of patients with tuberous sclerosis and epilepsy. Experience Children’s Hospital ̈Jose Manuel de los Río ̈ s. Epileptic Disord. 2014;16:145.
